# Enhanced mammalian genome editing by new Cas12a orthologs with optimized crRNA scaffolds

**DOI:** 10.1186/s13059-019-1620-8

**Published:** 2019-02-05

**Authors:** Fei Teng, Jing Li, Tongtong Cui, Kai Xu, Lu Guo, Qingqin Gao, Guihai Feng, Chuanyuan Chen, Dali Han, Qi Zhou, Wei Li

**Affiliations:** 1 State Key Laboratory of Stem Cell and Reproductive Biology, Institute of Zoology, Chinese Academy of Sciences, Beijing, 100101 China; 2 Institute for Stem Cell and Regeneration, Chinese Academy of Sciences, Beijing, 100101 China; 3 University of Chinese Academy of Sciences, Beijing, 100049 China; 4 School of Life Sciences, University of Science and Technology of China, Hefei, 230026 China; 5 Key Laboratory of Genomic and Precision Medicine, Beijing Institute of Genomics, Chinese Academy of Sciences, Beijing, 100101 China

**Keywords:** CRISPR-Cas12a/Cpf1, crRNA, Genome editing, Human cells, Mouse cells

## Abstract

CRISPR-Cas12a/Cpf1, a single RNA-guided endonuclease system, provides a promising tool for genome engineering. However, only three Cas12a orthologs have been employed for mammalian genome editing, and the editing efficiency as well as targeting coverage still requires improvements. Here, we harness six novel Cas12a orthologs for genome editing in human and mouse cells, some of which utilize simple protospacer adjacent motifs (PAMs) that remarkably increase the targeting range in the genomes. Moreover, we identify optimized CRISPR RNA (crRNA) scaffolds that can increase the genome editing efficiency of Cas12a.

**Electronic supplementary material:**

The online version of this article (10.1186/s13059-019-1620-8) contains supplementary material, which is available to authorized users.

## Background

Clustered regularly interspaced short palindromic repeats (CRISPR)-Cas12a/Cpf1 is the type V A CRISPR-Cas (CRISPR-associated proteins) system that has been recently harnessed for genome editing [1]. Several unique features make Cas12a distinguished from Cas9, providing a substantial expansion of CRISPR-based genome-editing tools. First, Cas12a is a single crRNA-guided endonuclease [1], while Cas9 is guided by a dual-RNA system consisting of a crRNA and a trans-activating crRNA (tracrRNA) [2]. Second, Cas12a recognizes a 5′ T-rich protospacer adjacent motif (PAM) [1], different from the 3′ G-rich PAM utilized by Cas9 [3, 4]. Third, after cleavage of double-stranded DNAs (dsDNAs), Cas12a generates staggered ends distal to the PAM site [1], whereas Cas9 introduces blunt ends within the PAM-proximal target site [5]. Moreover, RuvC and Nuc domains of Cas12a are responsible for target DNA cleavage [6], whereas Cas9 uses the RuvC and HNH endonuclease domains to cleave the target DNAs [3]. While these diverse properties of the CRISPR-Cas12a system provide potential for the development of versatile tools for genome engineering [1, 7–11], there are still challenges, including few currently identified orthologs, limited genomic targeting coverage, and relatively low editing efficiency [1, 12–15]. To address these limitations, we aimed to identify novel Cas12a nucleases with simpler PAM requirements which can increase its targeting range and engineer the crRNA scaffold to achieve higher efficiencies of genome editing.

## Results and discussion

**Fig. 1:**
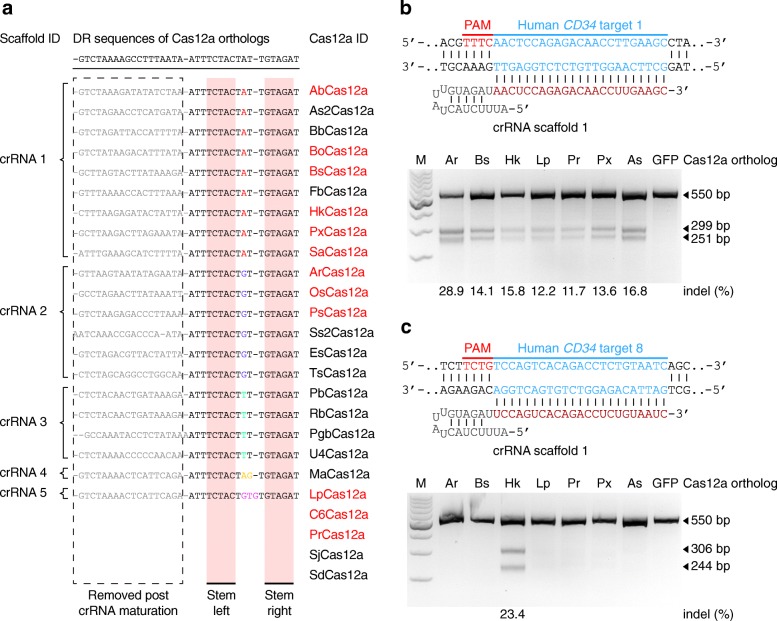
New Cas12a orthologs harnessed to robustly edit mammalian genomes. **a** Alignment of direct repeats (DRs) from the 25 Cas12a family proteins. Note that 4 Cas12a bacterial loci contain no DR array. Non-conserved bases in the loop are colored, and the conserved stem duplex is highlighted in pink. **b** (Upper) schematic showing the sequence of crRNA targeting the human *CD34* gene site 1. (Lower) T7EI analysis of targeted indel frequencies induced by the 6 Cas12a candidates (ArCas12a, BsCas12a, HkCas12a, LpCas12a, PrCas12a, and PxCas12a) as indicated. AsCas12a was used as positive control. GFP, an empty backbone vector without Cas12a protein expression. M, DNA marker. **c** (Upper) schematic showing the sequence of crRNA targeting the human *CD34* gene site 8. (Lower) T7EI assay result indicated that HkCas12a facilitated targeted indels directed by 5′-TCTN PAM. GFP, an empty backbone vector without Cas12a protein expression. M, DNA marker

To search for new Cas12a proteins for genome editing, we first used PSI-BLAST program [16] and identified 21 non-redundant CRISPR-Cas12a loci which have not previously been employed for genome editing and 4 Cas12a proteins (BoCas12a, BsCas12a, PbCas12a, and TsCas12a) recently being characterized during our paper submission [17] (Additional file 1: Supplementary Methods and Additional file 2: Figure S1a). The alignment of their mature crRNA sequences showed that they used 5 highly conserved crRNA scaffolds in total (scaffolds 1~5, loop region: UAUU, UGUU, UUUU, UAGU, and UGUGU, respectively) that only had sequence variations in the loop region (Fig. 1a and Additional file 2: Figure S1b), which is consistent with the previous report [1]. We synthesized 12 Cas12a genes coding for approximately 1200~1300 amino acids or less and 3 corresponding crRNA sequences (scaffolds 1, 2, and 5) (Additional file 1: Supplementary Methods, Additional file 2: Figure S1b, c, Additional file 3: Table S1, Additional file 4: Table S2, and Additional file 5: Supplementary Sequences). Then, we purified the Cas12a proteins expressed in *E*. *coli* cells (Additional file 2: Figure S2a) and incubated them with in vitro-transcribed crRNAs and dsDNA substrates for in vitro DNA cleavage assay (Additional file 2: Figure S2b and Additional file 4: Table S3). The conservation of the crRNA scaffolds suggested that these Cas12a proteins might also recognize the 5′ T-rich PAM as the previous report indicated [1]. Indeed, we found that crRNA scaffold 1, 2, or 5 enables Cas12a proteins to cleave the target DNAs with a 5′-TTTN or even 5′-TTN PAM in vitro (Additional file 2: Figure S2c). To further characterize the PAM requirements, we analyzed the Cas12a cleavage activity on dsDNA substrates bearing 5′-TTN, 5′-TNN, and 5′-NTN PAMs (Additional file 4: Table S3). We found that ArCas12a, BsCas12a, and PrCas12a recognized 5′-TTN PAM for dsDNA cleavage, and HkCas12a recognized a simple 5′-YYN PAM (Y:T or C) (Additional file 2: Figure S2d).

Next, we explored the capability of these Cas12a orthologs to cleave the target genomic sequences in mammalian cells. The 12 synthesized Cas12a genes fused with 2 nuclear localization signals (NLSs) at each end were constructed into mammalian expression vectors for Cas12a expression in human and mouse cells (Additional file 2: Figure S3a and Additional file 5: Supplementary Sequences). After transfection, the immunofluorescence staining results showed clear nuclear compartmentalization of the Cas12a proteins in mammalian cells (Additional file 2: Figure S3a). Then, we co-transfected human embryonic kidney 293FT cells or mouse embryonic stem cells (ESCs) with individual Cas12a orthologs and crRNAs (scaffold 1) to target endogenous loci containing the 5′ T-rich PAMs. Results of T7 endonuclease I (T7EI) assay showed that 6 Cas12a nucleases (ArCas12a, BsCas12a, HkCas12a, LpCas12a, PrCas12a, and PxCas12a) could all facilitate genome editing in both human and mouse genomes with the 5′-TTTN PAM (Fig. 1b and Additional file 2: Figure S3b) or 5′-TTN PAM (Additional file 2: Figure S3c). Sanger sequencing results further confirmed the capacity of these 6 Cas12a nucleases to introduce insertions or deletions (indels) at target sites in the mammalian genomes (Additional file 2: Figure S3d-f). We next focused on exploring the in vivo PAM requirement of HkCas12a, which owned the simplest PAM (5′-YYN) in vitro (Additional file 2: Figure S2d). By targeting the human *AAVS1*, *CD34*, and *RNF2* loci in 293FT cells (Additional file 4: Table S4), we showed that HkCas12a induced indels at target sites with the 5′-YTN and 5′-TYYN PAMs (Additional file 2: Figure S4a, b). Then, we compared the genomic coverage ability of HkCas12a with the previously reported AsCas12a [1], by targeting the endogenous loci containing requisite PAMs in mammalian genomes. Notably, HkCas12a possessed an expanded genomic coverage capacity than did AsCas12a (Fig. 1c and Additional file 2: Figure S4c). These data demonstrated that we harnessed new Cas12a nucleases for mammalian genome editing with their PAMs determined as 5′-TTN, 5′-YTN, or 5′-TYYN in vivo, which markedly increases the targeting range of Cas12a nucleases in mammalian genomes.

**Fig. 2:**
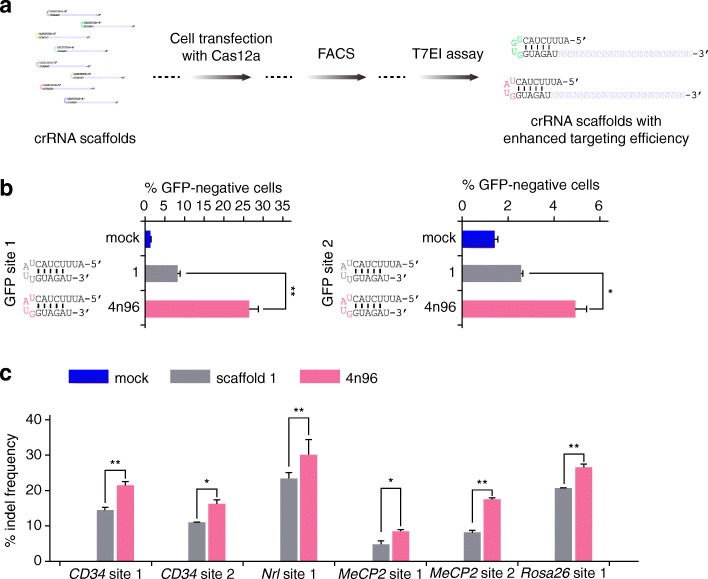
Enhanced targeted indel frequencies by optimized crRNA scaffold. **a** Schematic showing the workflow of crRNA scaffold optimization and screening. **b** Efficiencies of GFP disruption in human 293FT cells generated by PrCas12a/scaffold 1 and PrCas12a/scaffold 4n96, respectively. Error bars indicate standard errors of the mean (SEM), *n* = 3. *p value < 0.05 and **p value < 0.01. **c** PrCas12a-induced targeted indel frequencies at six different endogenous gene target sites with scaffold 1 and scaffold 4n96. Error bars indicate SEM, *n* = 3. *p value < 0.05 and **p value < 0.01

Interestingly, our in vitro DNA cleavage results showed that the crRNA scaffolds carrying nucleotide (nt) substitutions had variable effects on the cleavage activity of Cas12a nucleases (Additional file 2: Figure S2c), which is consistent with the previous report [1]. To test such effects in mammalian cells, three naturally existed crRNA scaffolds (scaffold 1, 2, or 5) and two artificial scaffolds (scaffold 6 or 7) were individually transfected into mouse ESCs with each of the six functional Cas12a nucleases to target the mouse *MeCP2* gene (Additional file 2: Figure S5a, Additional file 4: Table S5, and Additional file 5: Supplementary Sequences). Indel frequency analysis by T7EI assay showed that the highest cleavage efficiency was achieved by the Cas12a nuclease with its cognate crRNA scaffold in most cases (Additional file 2: Figure S5b), consistent with the previous report [13, 18]. Surprisingly, the Cas12a nuclease with artificial crRNA scaffolds could also induce high indel frequency at target sites, such as LpCas12a/crRNA 6 and PrCas12a/crRNA 7 (Additional file 2: Figure S5b), suggesting the potential of increasing Cas12a cleavage efficiency by optimizing the loop sequences of crRNA scaffolds. To test this hypothesis, we synthesized 256 crRNA scaffolds bearing all the possible nucleotide substitutions at the 4-nt loop (Fig. 2a and Additional file 4: Table S5). Next, we used these crRNA variants with PrCas12a and BsCas12a, respectively, to target the mouse *Nrl* locus, and compared the targeting efficiencies of these crRNA variants with that of the crRNA scaffold 1. The results showed that the crRNA scaffold 4n96 (loop region: UAUG) exhibited an enhanced targeting efficiency compared with the original scaffold 1 when working together with both PrCas12a and BsCas12a (Additional file 2: Figure S6a, b). To confirm these findings, we used 10 crRNA scaffolds with nucleotide substitutions in a 3-nt loop, 25 crRNA scaffolds with nucleotide substitutions in a 5-nt loop, and 25 crRNA scaffolds with a 4-nt loop including the 4n96, to target another site within the mouse *MeCP2* gene locus (Additional file 4: Table S5). Consistent with the above results, the scaffold 4n96 was identified to exhibit a higher targeting efficiency compared with other crRNA scaffolds (Additional file 2: Figure S6c, d). Moreover, we further employed the GFP disruption assay to analyze the effect of crRNA scaffold 4n96 on the genome cleavage efficiency of Cas12a nucleases. The crRNA scaffolds 1 and 4n96 were transfected with PrCas12a and BsCas12a, respectively, to target the integrated GFP reporter. Compared with crRNA scaffold 1, the scaffold 4n96 substantially increased the Cas12a targeting efficiency at two sites of the GFP gene (Fig. 2b and Additional file 2: Figure S7a). Next, we tested the generality of this enhanced genomic cleavage activity mediated by scaffold 4n96 by examining more target sites and Cas12a nucleases. We targeted six independent sites from both human and mouse genomes by PrCas12a and BsCas12a individually. The results showed that compared with the crRNA scaffold 1, the scaffold 4n96 significantly increased the cleavage activity of both PrCas12a and BsCas12a at all these six sites (Fig. 2c and Additional file 2: Figure S7b). Moreover, the genome cleavage activity of the other three Cas12a nucleases (ArCas12a, HkCas12a, and PxCas12a) was increased using the crRNA scaffold 4n96 compared with using scaffold 1 (Additional file 2: Figure S7c). These results strongly suggested that the effect of enhanced Cas12a-mediated genome editing by the scaffold 4n96 was universal. Taken together, our results demonstrated that the crRNA scaffold 4n96 carrying nucleotide substitution at the loop region can markedly increase the Cas12a-mediated genome editing efficiencies.

We further characterized the targeting efficiency and specificity of these newly identified Cas12a nucleases. First, we directly compared the relative targeting activities of Cas12a and SpCas9, the efficiency of which is considered as the current gold standard for genome editing. By performing T7EI analyses of targeted indels of endogenous genomic sites, we found that the average targeting efficiencies of these Cas12a proteins (ArCas12a, BsCas12a, HkCas12a, and PrCas12a) are lower than SpCas9, although these Cas12a proteins could achieve higher targeting efficiencies when directed by crRNA 4n96 than crRNA 1 (Additional file 4: Table S7). Meanwhile, to address the off-targeting risks of Cas12a, we performed off-target predictions using Cas-OFFinder [19] followed by targeted deep sequencing. The results showed that both Cas12a (BsCas12a and PrCas12a) and SpCas9 exhibited a low incidence of off-target mutations at the endogenous *DNMT1* site 1 in targeted human 293FT cells (Additional file 4: Table S8). Moreover, the genome-wide off-target analysis by whole genome sequencing (WGS) also showed a low incidence of off-target mutations for both Cas12a and SpCas9 (Additional file 4: Table S9). All these data indicated the minimal off-target risks of Cas12a, which is consistent with previous reports [13, 14].

In this work, we report the identification of six new Cas12a nucleases for genome editing in mammalian cells, including one Cas12a ortholog (HkCas12a) recognizing more flexible 5′-YTN and 5′-TYYN PAMs that can provide broader genome coverage. However, the precise PAM sequences of these orthologs still need to be determined in the future using high-throughput approaches. The non-canonical PAM recognition by HkCas12a was possibly due to the variation of L642 residue, which was equivalent to K592 of LbCas12a and was responsible for the non-canonical PAM recognition (Additional file 2: Figure S8) [20, 21]. As previous studies indicated, crRNA scaffolds could affect or even enhance the targeting activities of CRISPR-Cas systems [1, 12, 13, 18]. Through engineering the nucleotide substitutions at the loop region, we identify a crRNA scaffold that markedly improves the Cas12a-mediated genome editing efficiency. The crystal structures of Cas12a-RNA-DNA complex have shown that the nucleotides in the loop region of crRNA scaffold interact with Cas12a residues [6, 22], indicating nucleotide substitutions in the loop region of crRNA scaffold would affect the activities of Cas12a-crRNA complex [18]. Further structural characterization of Cas12a-crRNA-DNA complexes with different crRNA scaffolds will help the elucidation of the exact mechanisms of this improvement in the future. Collectively, our findings expand the CRISPR-Cas12a genome editing toolbox and may enhance their application in mammalian genome engineering and human gene therapy.

## Methods

### Identify new CRISPR-Cas12a loci

PSI-BLAST program [16] was applied to identify Cas12a homologs in the NCBI non-redundant protein sequence database using AsCas12a and LbCas12a protein sequences [1]. Cas12a loci not yet harnessed for mammalian genome editing were chosen as candidates for analysis. CRISPR repeats were identified using CRISPRFinder [23].

### crRNA scaffold library construction

Paired degenerate primers were synthesized and annealed to form a duplex with 5′ overhangs (Additional file 4: Table S5). Then, they were constructed into an U6 promoter-driven expression vector (Additional file 5: Supplementary Sequences). The scaffold variants were randomly picked out from cultured plates and then sequenced.

### Cell culture, transfection, and fluorescence-activated cell sorting

Human embryonic kidney cell line 293FT and human cervical cancer cell line HeLa were cultured in Dulbecco’s modified Eagle’s medium (DMEM, Gibco) supplemented with 10% fetal bovine serum (FBS, Gibco) and 1% Antibiotic-Antimycotic (Gibco). Mouse embryonic stem (mES) cell line was maintained in N2B27 medium plus 2i (Stemgent) and mLIF (Millipore). The N2B27 medium consists of DMEM/F12 (Gibco) and Neurobasal (Gibco) at a ratio of 1:1 and was supplemented with 1% N-2 supplement (Gibco), 0.5% B-27 supplement (Gibco), 20 ng/ml BSA (Sigma), 10 μg/ml insulin (Roche), 1% GlutaMAX (Gibco), 5% knockout serum replacement (KOSR, Gibco), 0.1% β-mercaptoethanol (Gibco), and 1% Antibiotic-Antimycotic (Gibco). 293FT cells were transfected using Lipofectamine LTX (Invitrogen) following the manufacturer’s recommended protocol. mES cells were transfected via electroporation using Neon™ transfection system (Invitrogen) following the manufacturer’s recommended protocol. For each well of a 24-well plate, a total of 750 ng plasmids (Cas12a-2AeGFP: crRNA = 2: 1) was used. Then, 48 h following transfection, GFP-positive cells were sorted using the MoFlo XDP (Beckman Coulter).

### T7 endonuclease I assay for genome modification

Cells were collected after 48 h post-transfection for genomic DNA extraction. GFP-positive cells sorted by FACS were lysed directly using Buffer L (Bimake). The genomic region flanking the Cas12a targeting site of each gene was PCR-amplified (Additional file 4: Table S6), and products were purified using DNA Clean & Concentrator (ZYMO Research) following the manufacturer’s protocol. A total of ~ 200 ng purified PCR amplicons was mixed with 1 μl NEBuffer 2 (NEB) and diluted in ddH_2_O to 10 μl, then subjected to a re-annealing process to form a heteroduplex according to our previously reported procedure [24]. After re-annealing, the products were treated with T7EI (NEB) following recommending protocol, and 2.5% agarose gels (Takara) were used for further analysis. Indels were calculated via band intensities based on previously reported method [25].

### GFP disruption assay

Human 293FT.eGFP cells harboring a single-copy, integrated *AAVS1*-eGFP gene were generated by our lab. These cells were transfected with Cas12a expression plasmid and crRNA expression plasmid, or Cas12a expression plasmid and an U6 promoter-driven empty plasmid as a negative control using Lipofectamine LTX (Invitrogen). Three days post-transfection, cells were analyzed on the MoFlo XDP (Beckman Coulter). For each sample, transfections and flow cytometry measurements were performed in triplicate.

## Additional files

Additional file 1: Supplementary methods. (PDF 124 kb)

Additional file 2: **Figure S1.** Phylogeny tree of non-redundant Cas12a orthologs and selected Cas12a loci for genome editing. **Figure S2.** In vitro DNA cleavage assay for Cas12a PAM sequences. **Figure S3.** Six new Cas12a proteins mediated robust genome editing in mammalian cells. **Figure S4.** Increased genome-wide coverage of HkCas12a with altered PAMs. **Figure S5. **crRNA scaffold alters targeted indel efficiencies of Cas12a proteins. **Figure S6.** crRNA scaffold optimization and screening. **Figure S7.** Enhanced targeted efficiency with optimized crRNA scaffold. **Figure S8.** Conserved residues in PAM-interacting (PI) domain of Cas12a proteins. (PDF 18496 kb)

Additional file 3: **Table S1.** Oligonucleotides (oligos) for Cas12a gene synthesis. (XLSX 81 kb)

Additional file 4: **Table S2.** Sequences of targeting crRNAs used for in vitro RNA transcription. **Table S3.** Target sequences harboring various 5′ PAM sequences used for in vitro DNA cleavage assay. **Table S4.** Protospacer sequences used for genome editing. **Table S5.** crRNA scaffold optimization and screening. **Table S6.** Primer sequences used for PCR amplification. **Table S7.** Frequency of Cas12a- and SpCas9-mediated targeted indel mutations at on-target sites in human 293FT cells. **Table S8.** Off-target analysis by targeted deep sequencing in the human 293FT cells. **Table S9.** Off-target analysis by whole genome sequencing (WGS) in human 293FT cells. (PDF 395 kb)

Additional file 5: Supplementary Sequences. The humanized Cas12a coding sequences, protein sequences, U6-crRNA backbone sequences, mammalian and prokaryotic expression vector sequences used in this study. (PDF 183 kb)

## Abbreviations

Cas: CRISPR-associated protein
CRISPR: Clustered regularly interspaced short palindromic repeats
crRNA: CRISPR RNA
dsDNA: Double-stranded DNA
indel: Insertion or deletion
NLS: Nuclear localization signal
nt: Nucleotide
PAM: Protospacer adjacent motif
T7EI: T7 endonuclease I
tracrRNA: Trans-activating crRNA
