# Supplementary material for: Enhanced mammalian genome editing by new Cas12a orthologs with optimized crRNA scaffolds
Source: Genome Biol. 2019 Feb 5;20:15. doi: 10.1186/s13059-019-1620-8 (PMC6362571; doi:10.1186/s13059-019-1620-8)
Supplement: Supplementary file 1 — Supplementary methods. (PDF 124 kb) [file 13059_2019_1620_MOESM1_ESM.pdf]

Supplementary Methods

***De novo* gene synthesis and heterogeneous plasmid construction.** Coding sequences for CRISPR-Cas12a proteins were humanized [1] and oligonucleotides (oligos) for gene synthesis were designed using GeneDesign program [2]. For Cas12a coding genes which > 3 kb were split into 4 to 5 "chunks" of ~800 bp (Additional file 3: Table S1). All commercial oligos for gene synthesis were purchased from *Taihe Biotechnology Co.*. DNA fragments were synthesized using overlap extension PCR method according to our previous study [3]. Gel purified DNA fragments were seamlessly assembled into mammalian or prokaryotic expression vectors using HiFi DNA Assembly Master Mix (NEB). Detailed Cas12a coding sequences and plasmid sequences are available in Additional file 5: Supplementary Sequences.

Purification of Cas12a proteins. Cas12a proteins were purified according to our previous report [3]. Briefly, BPK2014-Cas12a-His_{10} protein was expression in *E. coli* strain BL21 (λDE3) and induced expression with 0.5 mM IPTG at 16 °C for 16 h. Cell pellets were harvested and lysed, following washing and elution using His60 Ni Superflow Resin (Takara). Purified Cas12 proteins were dialyzed, concentrated and finally quantitated using BCA Protein Assay Kit (Thermo Fisher).

***In vitro* transcription of crRNAs.** All crRNAs used in biochemical reactions were *in vitro*-transcribed using the HiScribe T7 High Yield RNA Syntheis Kit (NEB). Synthetic ssDNA oligos containing a T7 promoter sequence were annealed to dsDNA templates (Additional file 4: Table S2). T7 transcription was performed at 37 °C for 4 h, and transcribed RNA was purified using Oligo Clean & Concentrator (ZYMO Research).

***In vitro* DNA cleavage assay.** *In vitro* DNA cleavage was performed using purified Cas12a proteins (50 nM), target dsDNAs (200 ng) and synthesized crRNAs (400 ng) at 37 °C in NEBuffer 3 (NEB) in a volume of 10 μL for 1 h. Target DNAs with protospacers containing various PAM sequences were PCR amplified (Additional file 4: Tables S3 and S6). Reaction was stopped by incubation at 75 °C for 5 min, then run on 2.5% agarose gels (Takara).

Immunofluorescence staining. Cells were fixed with 4% paraformaldehyde (PFA) for 15 min and were then permeabilized with 0.5% Triton X-100 (Sigma-Aldrich) for 30 min, followed by blocking with 2% BSA. The cells were then incubated in the primary antibodies anti-HA High Affinity (1:500, Roche) overnight at 4°C and secondary antibody at room temperature for 1 h. Nuclei were stained with DAPI (Sigma-Aldrich). The images were captured under a laser-scanning confocal microscope (ZEISS, LSM 780).

Sanger Sequencing of mutated sites. The target sites were amplified by PCR with specific primers (Additional file 4: Table S6) from genomic DNA of the mutated cells identified by T7EI assay. The PCR products were cloned into the pEASY-Blunt vector (Transgen) and transformed to competent Trans1-T1 (Transgen) *E. coli* cells. After overnight culture at 37 ºC, emerged colonies were randomly picked out and sequenced. Mutations were identified by alignment of sequenced alleles to wild type allele.

Off-target prediction and detection. The potential off-target sites for CRISPR-Cas12b system in the human or mouse genome with individual on-target sequences were predicted using Cas-OFFinder [4]. The complementarity region bearing one, two or three mismatches with requisite PAMs were assessed as potential off-targets by T7EI assay or targeted deep sequencing.

Targeted deep sequencing. Target sites and potential off-target sites were amplified by barcoded PCR and pooled libraries were subjected to paired-end sequencing using MiSeq (Illumina). A reference genome was built using Picard Tools (http://broadinstitute.github.io/picard) and samtools [5] from DNA sequences of the considered on-/off-target regions. Raw sequencing data (FASTQ files) were mapped against the created reference genome using BWA [6] with standard parameters and resulting alignment files were sorted using smatools. Samples with fewer than 20 reads were excluded.

Whole genome sequencing (WGS). Genomic DNAs from cultured cells were extracted using MicroElute Genomic DNA Kit (OMEGA) and subjected to quality assessment. The extracted

DNA was sequenced using an Illumina NovaSeq sequencer at a sequencing depth of 30x diploid coverage. The pair-ends reads were aligned onto the *hg19* (GRCh38) human reference genome using Bowtie 2 [7] and BWA [6], following manipulated using Picard Tools (http://broadinstitute.github.io/picard), respectively.

The WGS analysis was performed according to previous report [8]. Briefly, the Genome Analysis ToolKit (GATK4) [9] or pysamstats (https://github.com/alimanfoo/pysamstats) were used for local realignment around indels, base score recalibration, variant calling across the human samples and variant score recalibration. Candidate indels were filtered on several criteria using Python, PyVCF and PyFasta packages. First, we removed indels near low-complexity regions as defined by RepeatMasker (http://repeatmasker.org) and annotated by softmasking in *hg19*. Second, we removed indels that caused expansions or compressions of long (one with> 6 bp or two with ≥5 bp) homopolymers.

Supplementary References

1. Grote A, Hiller K, Scheer M, Munch R, Nortemann B, Hempel DC, Jahn D: **JCat: a novel tool to adapt codon usage of a target gene to its potential expression host.** *Nucleic Acids Res* 2005, **33:**W526-531.
2. Richardson SM, Wheelan SJ, Yarrington RM, Boeke JD: **GeneDesign: rapid, automated design of multikilobase synthetic genes.** *Genome Res* 2006, **16:**550-556.
3. Teng F, Cui T, Feng G, Guo L, Xu K, Gao Q, Li T, Li J, Zhou Q, Li W: **Repurposing CRISPR-Cas12b for mammalian genome engineering.** *Cell Discov* 2018, **4:**63.
4. Bae S, Park J, Kim JS: **Cas-OFFinder: a fast and versatile algorithm that searches for potential off-target sites of Cas9 RNA-guided endonucleases.** *Bioinformatics* 2014, **30:**1473-1475.
5. Li H, Handsaker B, Wysoker A, Fennell T, Ruan J, Homer N, Marth G, Abecasis G, Durbin R, Genome Project Data Processing S: **The Sequence Alignment/Map format and SAMtools.** *Bioinformatics* 2009, **25:**2078-2079.
6. Li H, Durbin R: **Fast and accurate long-read alignment with Burrows-Wheeler transform.** *Bioinformatics* 2010, **26:**589-595.
7. Langmead B, Salzberg SL: **Fast gapped-read alignment with Bowtie 2.** *Nat Methods* 2012, **9:**357-359.
8. Veres A, Gosis BS, Ding Q, Collins R, Ragavendran A, Brand H, Erdin S, Cowan CA, Talkowski ME, Musunuru K: **Low incidence of off-target mutations in individual CRISPR-Cas9 and TALEN targeted human stem cell clones detected by whole-genome sequencing.** *Cell Stem Cell* 2014, **15:**27-30.
9. McKenna A, Hanna M, Banks E, Sivachenko A, Cibulskis K, Kernytsky A, Garimella K, Altshuler D, Gabriel S, Daly M, DePristo MA: **The Genome Analysis Toolkit: a MapReduce framework for analyzing next-generation DNA sequencing data.**

Genome Res 2010, **20:**1297-1303.
