# Supplementary material for: Enhanced mammalian genome editing by new Cas12a orthologs with optimized crRNA scaffolds
Source: Genome Biol. 2019 Feb 5;20:15. doi: 10.1186/s13059-019-1620-8 (PMC6362571; doi:10.1186/s13059-019-1620-8)
Supplement: Supplementary file 2 — Figure S1. Phylogeny tree of non-redundant Cas12a orthologs and selected Cas12a loci for genome editing. Figure S2. In vitro DNA cleavage assay for Cas12a PAM sequences. Figure S3. Six new Cas12a proteins mediated robust genome editing in mammalian cells. Figure S4. Increased genome-wide coverage of HkCas12a with altered PAMs. Figure S5. crRNA scaffold alters targeted indel efficiencies of Cas12a proteins. Figure S6. crRNA scaffold optimization and screening. Figure S7. Enhanced targeted efficiency with optimized crRNA scaffold. Figure S8. Conserved residues in PAM-interacting (PI) domain of Cas12a proteins. (PDF 18496 kb) [file 13059_2019_1620_MOESM2_ESM.pdf]

a

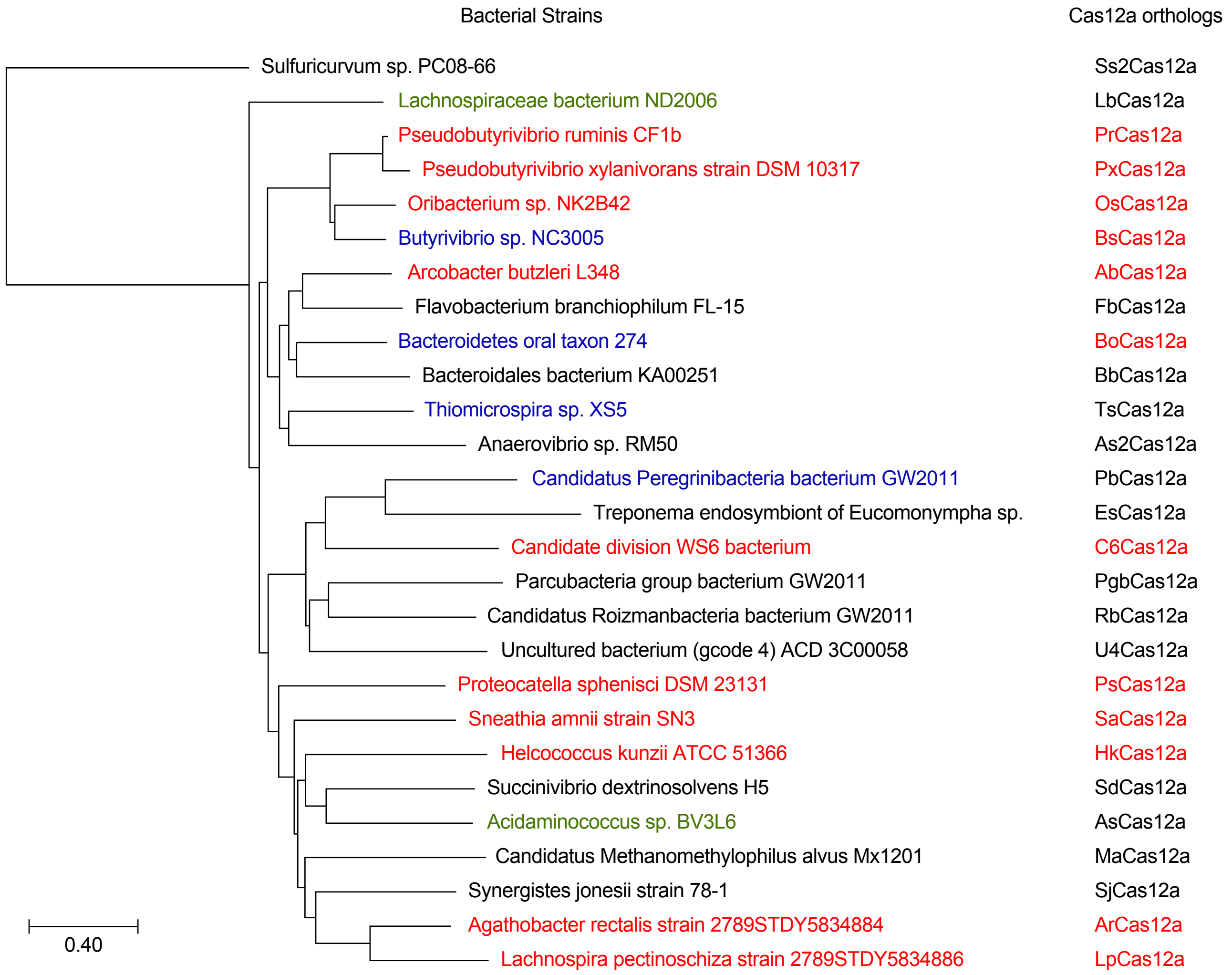

Bacterial Strains
Cas12a orthologs
Sulfuricurvum sp. PC08-66
Ss2Cas12a
Lachnospiraceae bacterium ND2006
LbCas12a
Pseudobutyrivibrio ruminis CF1b
PrCas12a
Pseudobutyrivibrio xylanivorans strain DSM 10317
PxCas12a
Oribacterium sp. NK2B42
OsCas12a
Butyrivibrio sp. NC3005
BsCas12a
Arcobacter butzleri L348
AbCas12a
Flavobacterium branchiophilum FL-15
FbCas12a
Bacteroidetes oral taxon 274
BoCas12a
Bacteroidales bacterium KA00251
BbCas12a
Thiomicrospira sp. XS5
TsCas12a
Anaerovibrio sp. RM50
As2Cas12a
Candidatus Peregrinibacteria bacterium GW2011
PbCas12a
Treponema endosymbiont of Eucomonympha sp.
EsCas12a
Candidate division WS6 bacterium
C6Cas12a
Parcubacteria group bacterium GW2011
PgbCas12a
Candidatus Roizmanbacteria bacterium GW2011
RbCas12a
Uncultured bacterium (gcode 4) ACD 3C00058
U4Cas12a
Proteocatella sphenisci DSM 23131
PsCas12a
Sneathia amnii strain SN3
SaCas12a
Helcococcus kunzii ATCC 51366
HkCas12a
Succinivibrio dextrinosolvens H5
SdCas12a
Acidaminococcus sp. BV3L6
AsCas12a
Candidatus Methanomethylophilus alvus Mx1201
MaCas12a
Synergistes jonesii strain 78-1
SjCas12a
Agathobacter rectalis strain 2789STDY5834884
ArCas12a
Lachnospira pectinoschiza strain 2789STDY5834886
LpCas12a
0.40

b

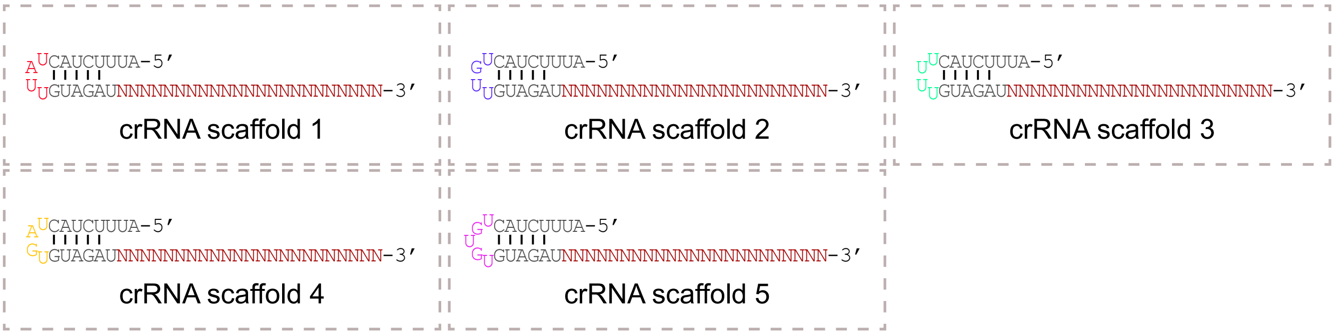

CAUCUUUA-5′
crRNA scaffold 1
CAUCUUUA-5′
crRNA scaffold 2
CAUCUUUA-5′
crRNA scaffold 3
CAUCUUUA-5′
crRNA scaffold 4
CAUCUUUA-5′
crRNA scaffold 5

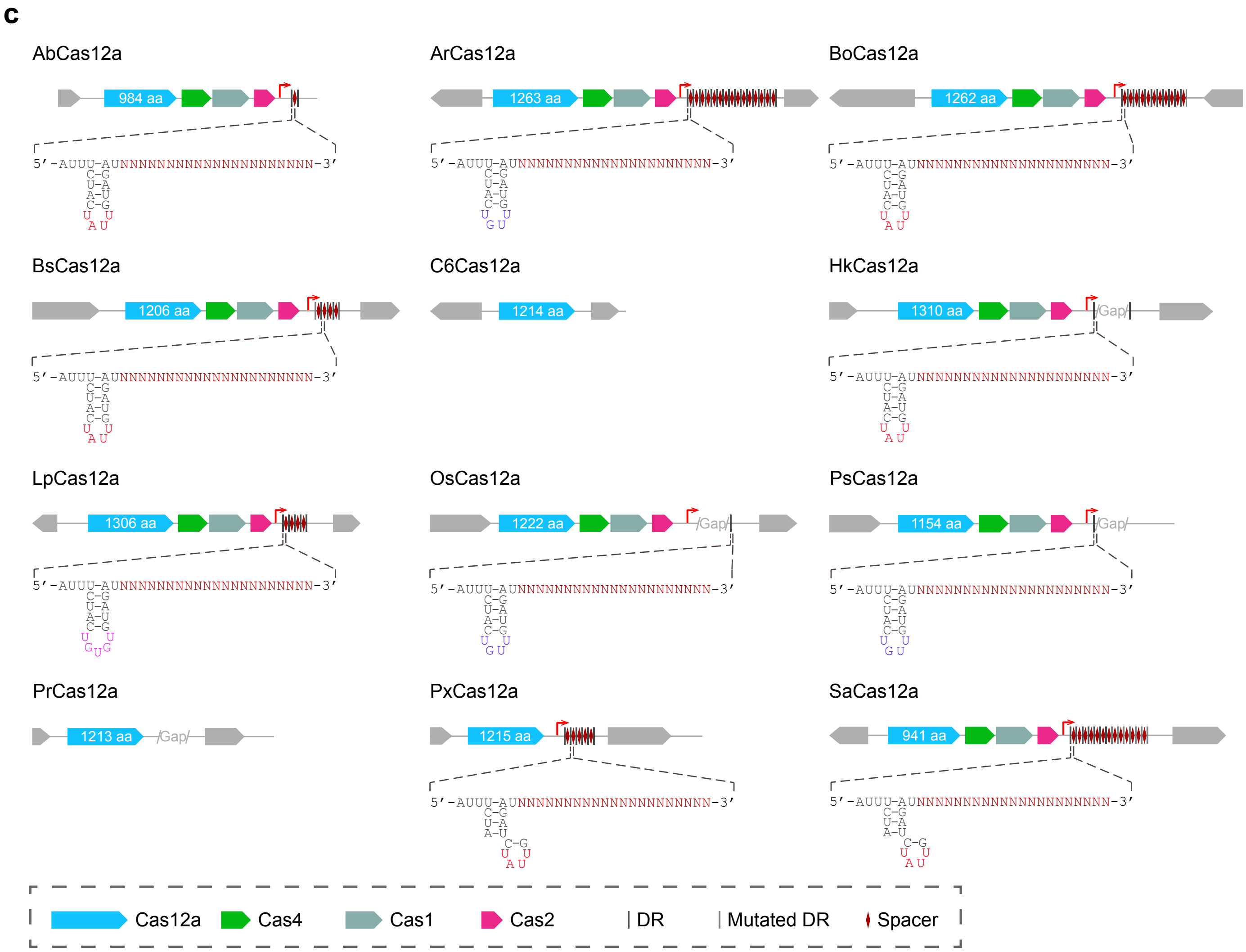

Figure S1. Phylogeny tree of non-redundant Cas12a orthologs and selected Cas12a loci for genome editing. **a** Evolutionary relationships of the Cas12a bacterial strains. Candidates synthesized for further functional experimentation were highlighted in red. **b** Schematic showing the 5 naturally existed crRNA scaffolds from the 25 Cas12a bacterial strains in Figure 1a. **c** Maps of bacterial genomic CRISPR-Cas12a loci corresponding to the 12 candidates selected to be tested.

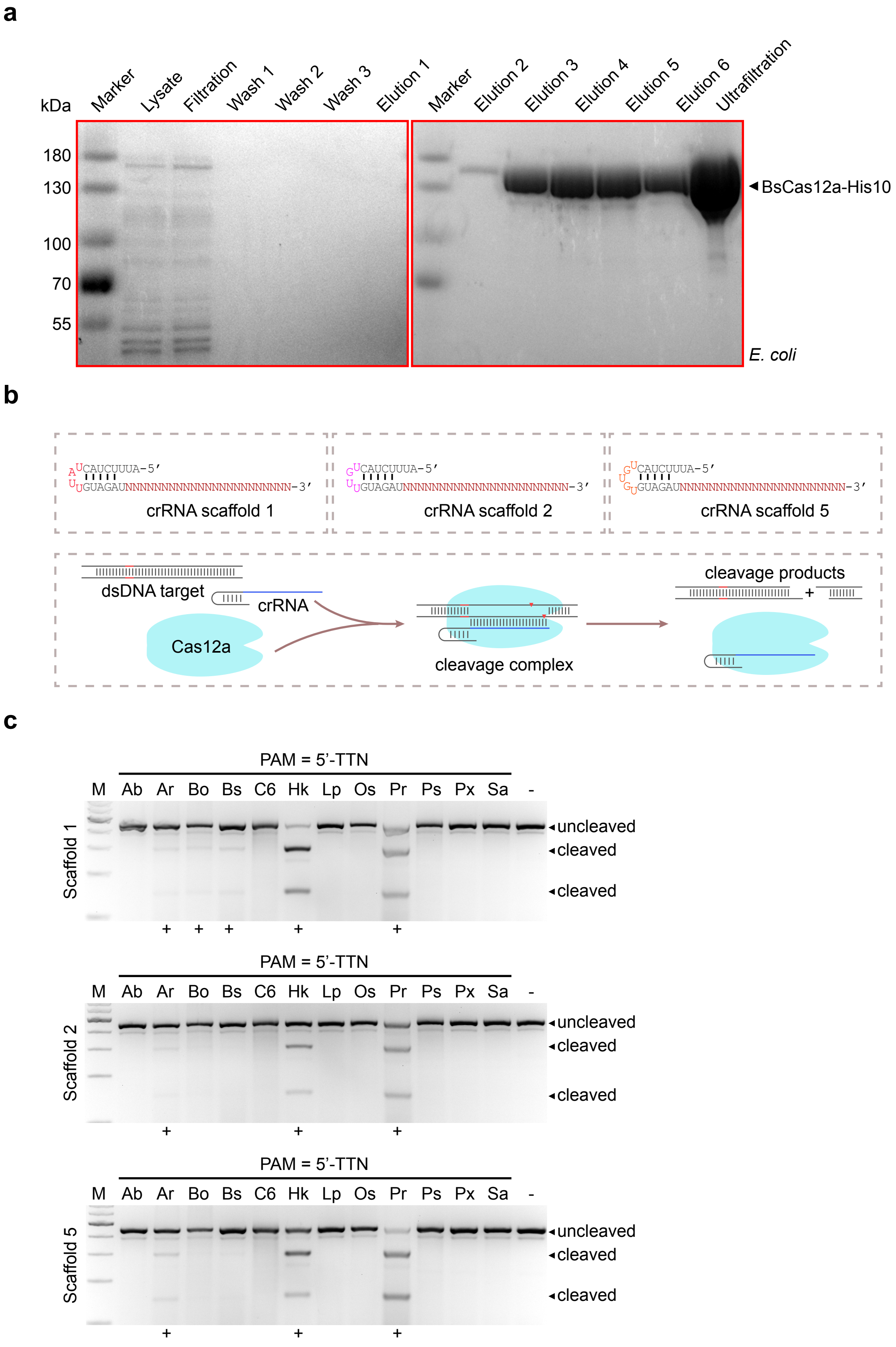
a
kDa
Marker
Lysate
Filtration
Wash 1
Wash 2
Wash 3
Elution 1
Marker
Elution 2
Elution 3
Elution 4
Elution 5
Elution 6
Ultrafiltration
180
130
100
70
55
BsCas12a-His10
E. coli
b
crRNA scaffold 1
crRNA scaffold 2
crRNA scaffold 5
dsDNA target
crRNA
Cas12a
cleavage complex
cleavage products
c
PAM = 5'-TTN
M Ab Ar Bo Bs C6 Hk Lp Os Pr Ps Px Sa -
Scaffold 1
uncleaved
cleaved
cleaved
+ + + + +
PAM = 5'-TTN
M Ab Ar Bo Bs C6 Hk Lp Os Pr Ps Px Sa -
Scaffold 2
uncleaved
cleaved
cleaved
+ + +
PAM = 5'-TTN
M Ab Ar Bo Bs C6 Hk Lp Os Pr Ps Px Sa -
Scaffold 5
uncleaved
cleaved
cleaved
+ + +

d

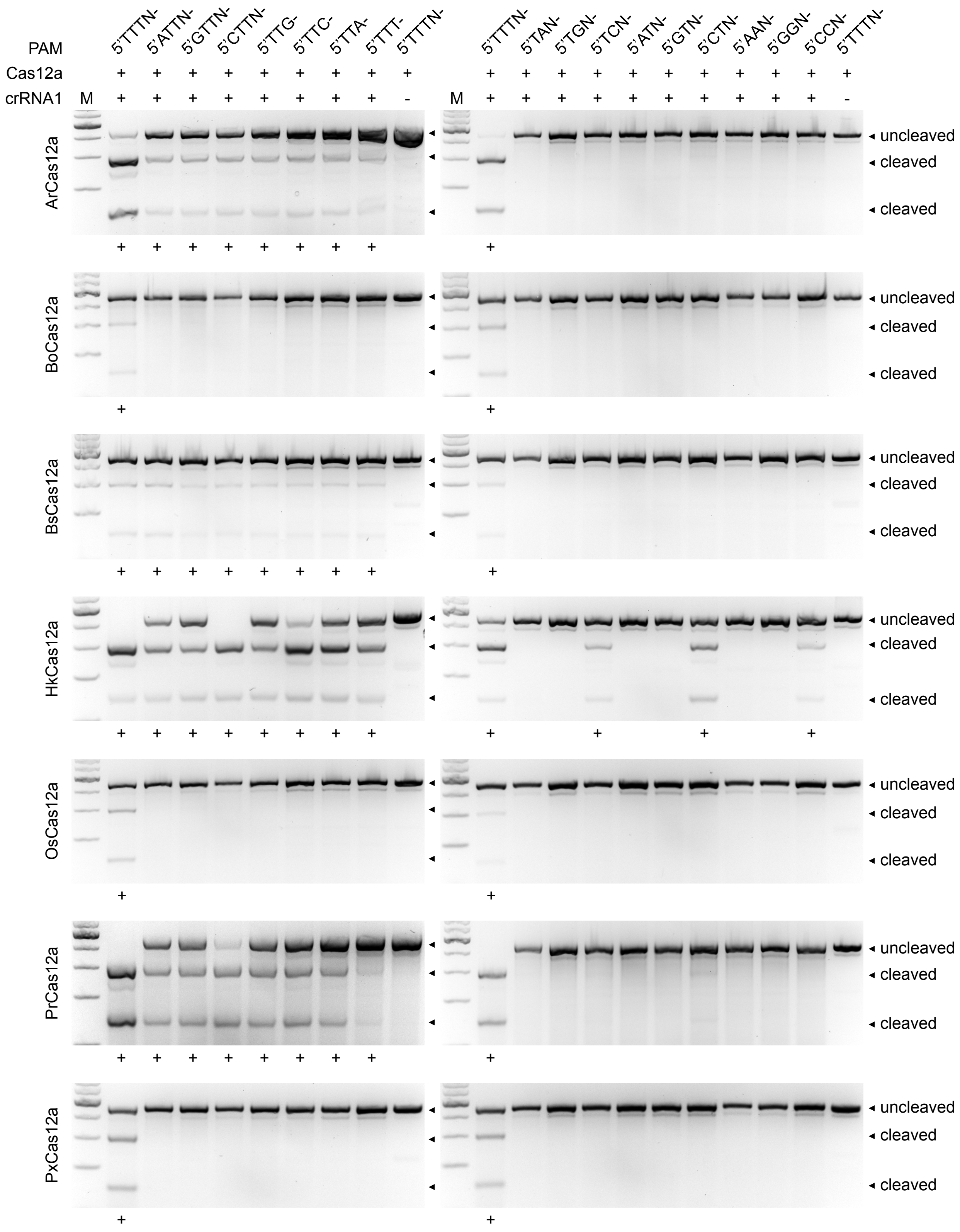
PAM
Cas12a
crRNA1
M
5'TTTN-
5'ATTN-
5'GTTN-
5'CTTN-
5'TTG-
5'TTC-
5'TTA-
5'TTT-
5'TTTN-
5'TAN-
5'TGN-
5'TCN-
5'ATN-
5'GTN-
5'CTN-
5'AAN-
5'GGN-
5'CCN-
ArCas12a
BoCas12a
BsCas12a
HkCas12a
OsCas12a
PrCas12a
PxCas12a
uncleaved
cleaved
cleaved

Figure S2. *In vitro* DNA cleavage assay for Cas12a PAM sequences. **a** Coomassie blue stained SDS-PAGE of *E. coli* expressed BsCas12a stepwise purification as a representative. The black triangle indicates Cas12a-His_{10} fused protein. **b** (*Upper*) Schematic showing the 3 naturally existed crRNA scaffolds used for *in vitro* DNA cleavage assay. (*Lower*) Depiction of workflow of *in vitro* DNA cleavage assay. **c** *In vitro* DNA cleavage assay using the 12 candidate Cas12a proteins directed by crRNA scaffold 1, 2 and 5, respectively. The PCR-generated templates contained 5'-T rich PAM sequences. N = A, T, G, C. M, DNA marker. **d** *In vitro* DNA cleavage analysis of the seven Cas12a nucleases possessing *in vitro* nuclease activity in **Figure S2c** to target various 5' PAM sequences directed by crRNA scaffold 1. N = A, T, G, C. M, DNA marker.

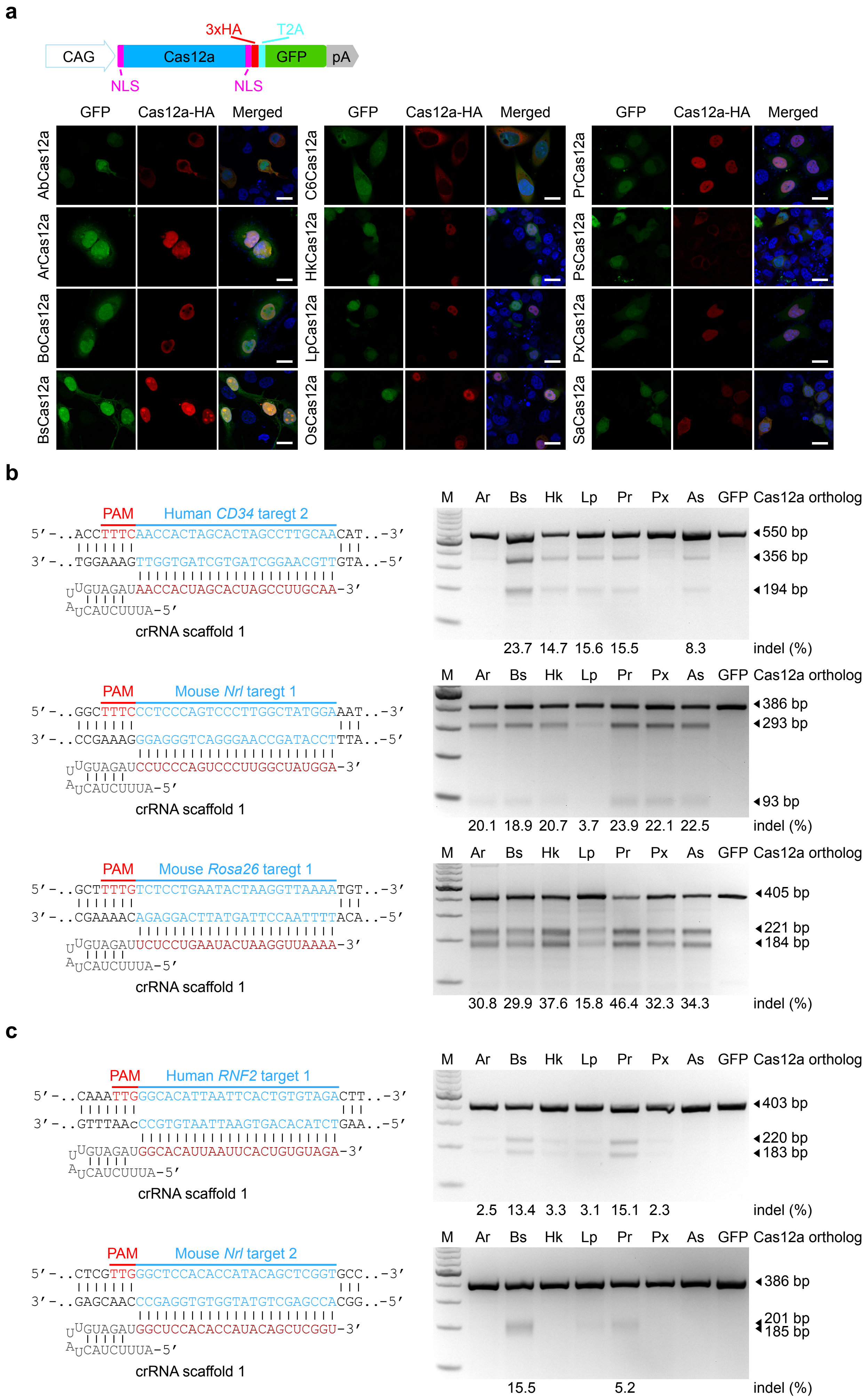
a
CAG
Cas12a
GFP
pA
3xHA
T2A
NLS
NLS
GFP
Cas12a-HA
Merged
AbCas12a
ArCas12a
BoCas12a
BsCas12a
C6Cas12a
HkCas12a
LpCas12a
OsCas12a
PrCas12a
PsCas12a
PxCas12a
SaCas12a
b
PAM
Human CD34 taregt 2
5′-..ACCTTTCAACCACTAGCACTAGCCTTGCAACAT..-3′
3′-..TGGAAAGTTGGTGATCGTGATCGGAACGTTGTA..-5′
UUGUAGAUAACCACUAGCACUAGCCUUGCAA-3′
AUCAUCUUUA-5′
crRNA scaffold 1
M Ar Bs Hk Lp Pr Px As GFP Cas12a ortholog
550 bp
356 bp
194 bp
23.7 14.7 15.6 15.5 8.3 indel (%)
PAM
Mouse Nrl taregt 1
5′-..GGCTTTCCCTCCCAGTCCCTTGGCTATGGAAAT..-3′
3′-..CCGAAAGGGAGGGTCAGGGAACCGATACCTTTA..-5′
UUGUAGAUCCUCCCAGUCCCUUGGCUAUGGA-3′
AUCAUCUUUA-5′
crRNA scaffold 1
M Ar Bs Hk Lp Pr Px As GFP Cas12a ortholog
386 bp
293 bp
93 bp
20.1 18.9 20.7 3.7 23.9 22.1 22.5 indel (%)
PAM
Mouse Rosa26 taregt 1
5′-..GCTTTTGTCTCCTGAATACTAAGGTTAAAATGT..-3′
3′-..CGAAAACAGAGGACTTATGATTCCAATTTTACA..-5′
UUGUAGAUUCUCCUGAAUACUAAGGUUAAAA-3′
AUCAUCUUUA-5′
crRNA scaffold 1
M Ar Bs Hk Lp Pr Px As GFP Cas12a ortholog
405 bp
221 bp
184 bp
30.8 29.9 37.6 15.8 46.4 32.3 34.3 indel (%)
c
PAM
Human RNF2 target 1
5′-..CAAATTGGGCACATTAATTCACTGTGTAGACTT..-3′
3′-..GTTTAAcCCGTGTAATTAAGTGACACATCTGAA..-5′
UUGUAGAUGGCACAUUAAUUCACUGUGUAGA-3′
AUCAUCUUUA-5′
crRNA scaffold 1
M Ar Bs Hk Lp Pr Px As GFP Cas12a ortholog
403 bp
220 bp
183 bp
2.5 13.4 3.3 3.1 15.1 2.3 indel (%)
PAM
Mouse Nrl target 2
5′-..CTCGTTGGGCTCCACACCATACAGCTCGGTGCC..-3′
3′-..GAGCAACCCGAGGTGTGGTATGTCGAGCCACGG..-5′
UUGUAGAUGGCUCCACACCAUACAGCUCGGU-3′
AUCAUCUUUA-5′
crRNA scaffold 1
M Ar Bs Hk Lp Pr Px As GFP Cas12a ortholog
386 bp
201 bp
185 bp
15.5 5.2 indel (%)

d

Human *CD34* target 1

ArCas12a

```
CCACCCCAGCCAACGTTTCAACTCCAGAGACAACCTT-------GA-AGCCTAG-CCTGTCACCTGGAAATGTTTCAGACCT
CCACCCCAGCCAACGTTTCAACTCCAGAGACAACctttccaggtgacaggctaggCCTGTCACCTGGAAATGTTTCAGACCT
CCACCCCAGCCAACGTTTCAACTCCAGAGACA----T-------GA-AGCCTAG-CCTGTCACCTGGAAATGTTTCAGACCT
CCACCCCAGCCAACGTTTCAACTCCAGAGACAA------------------------TGTCACCTGGAAATGTTTCAGACCT
CCACCCCAGCCAACGTTTCAACTCCAGAGACAACCTT-------------------------------AATGTTTCAGACCT
CCACCC---------------------------------agcctGA-AGCCTAG-CCTGTCACCTGGAAATGTTTCAGACCT
CCACCCCAGCCA-------------------------------------------CCTGTCACCTGGAAATGTTTCAGACCT
CCACCCCAGCCAACGTTTCAACTCCAGAGACA-----------------------------ACCTGGAAATGTTTCAGACCT
CCACCCCAGCCAACGTTTCAACTCCAGAGAC-----------------------------------(Δ 93 bp)-------
```

BsCas12a

```
CCACCCCAGCCAACGTTTCAACTCCAGAGACAACC--TTGAAGCCTAGCCTGTCACCTGGAAATGTTTCAGACCTTTCAACC
CCACCCCAGCCAACGTTTCAACTCCAGAGACAACCgaagGAAGCCTAGCCTGTCACCTGGAAATGTTTCAGACCTTTCAACC
CCACCCCAGCCAACGTTTCAACTCCAGAGACA--C--TTGAAGCCTAGCCTGTCACCTGGAAATGTTTCAGACCTTTCAACC
CCACCCCAGCCAACGTTTCAACTCCAGAGACAAC----TGAAGCCTAGCCTGTCACCTGGAAATGTTTCAGACCTTTCAACC
CCACCCCAGCCAACGTTTCAACTCCAGAGACA--------AAGCCTAGCCTGTCACCTGGAAATGTTTCAGACCTTTCAACC
CCACCCCAGCCAACGTTTCAACTCCAGAGACA---------AGCCTAGCCTGTCACCTGGAAATGTTTCAGACCTTTCAACC
CCACCCCAGCCAACGTTTCAACTCCAGAGACA----------GCCTAGCCTGTCACCTGGAAATGTTTCAGACCTTTCAACC
CCACCCCAGCCAACGTTT----------------------AAGCCTAGCCTGTCACCTGGAAATGTTTCAGACCTTTCAACC
```

HkCas12a

```
CCACCCCAGCCAACGTTTCAACTCCAGAGACAACCTTG-------------------------------AAGCCTAGCCTGT
CCACCCCAGCCAACGTTTCAACTCCAGAGACAACCTTGccctgggatgcaaggctggttcaacatatgcaaatCTAGCCTGT
CCACCCCAGCCAACGTTTCAACTCCAGAGACAACCTTGaagcctttg----------------------AAGCCTAGCCTGT
CCACCCCAGCCAACGTTTCAACTCCAGAGACAACCTT--------------------------------------AGCCTGT
CCACCCCAGCCAACGTTTCAACTCCAGAGACA--------------------------------------------ggCTGT
CCACCCCAGCCAACGTTTCAACTCCAGA---------------------------------------------CTAGCCTGT
```

LpCas12a

```
CCACCCCAGCCAACGTTTCAACTCCAGAGACAACCTTGAAGCCTAGCCTGTCACCTGGAAATGTTTCAGACCTTTCAACCAC
CCACCCCAGCCAACGTTTCAACTCCAGAGACAA---aGAAGCCTAGCCTGTCACCTGGAAATGTTTCAGACCTTTCAACCAC
CCACCCCAGCCAACGTTTCAACTCCAGAGACAACCTcga-----AGCCTGTCACCTGGAAATGTTTCAGACCTTTCAACCAC
CCACCCCAGCCAACGTTTCAACTCCAGAGACAACCT-------------------TGGAAATGTTTCAGACCTTTCAACCAC
```

PrCas12a

```
CCACCCCAGCCAACGTTTCAACTCCAGAGACAACCTTGAAGCCTAGCCTGTCACCTGGAAATGTTTCAGACCTTTCAACCAC
CCACCCCAGCCAACGTTTCAACTCCAGAGACAAC---GAAGCCTAGCCTGTCACCTGGAAATGTTTCAGACCTTTCAACCAC
CCACCCCAGCCAACGTTTCAACTCCAGAGACAACCT----GCCTAGCCTGTCACCTGGAAATGTTTCAGACCTTTCAACCAC
CCACCCCAGCCAACGTTTCAACTCCAGAGACA------AAGCCTAGCCTGTCACCTGGAAATGTTTCAGACCTTTCAACCAC
CCACCCCAGCCAACGTTTCAACTCCAGAGACAAC---------------------TGGAAATGTTTCAGACCTTTCAACCAC
CCACCCCAGCCAACGTTTCAACTCCAGAGACAA--------------------(Δ 118 bp)--------------------
```

PxCas12a

```
CCACCCCAGCCAACGTTTCAACTCCAGAGACAACCTTGAAGCCTAGCCTGTCACCTGGAAATGTTTCAGACCTTTCAACCAC
CCACCCCAGCCAACGTTTCAACTCCAGAGACAACCT---AGCCTAGCCTGTCACCTGGAAATGTTTCAGACCTTTCAACCAC
CCACCCCAGCCAACGTTTCAACTCCAGAGAC--------AGCCTAGCCTGTCACCTGGAAATGTTTCAGACCTTTCAACCAC
CCACCCCAGCCAACGTTTCAACTCCAGAGACA--------ggCTAGCCTGTCACCTGGAAATGTTTCAGACCTTTCAACCAC
CCACCCCAGCCAACGTTTCAACTCCAGAGACAACCT-------------GTCACCTGGAAATGTTTCAGACCTTTCAACCAC
CCACCCCAGCCAACGTTTCAACTCCAGAGACAACCT--------------------GGAAATGTTTCAGACCTTTCAACCAC
CCACCCCAGCCAACG-----------------------AAGCCTAGCCTGTCACCTGGAAATGTTTCAGACCTTTCAACCAC
CCACCCCAGCCAACGTTTCAACTCC------------------------------------------AGACCTTTCAACCAC
```

e

Human *CD34* target 2

```
BsCas12a
GAAATGTTTCAGACCTTTCAACCACTAGCACTAGCCTTGCAACATCTCCCACTAAACCCTATACATCATCTTCTCCT
GAAATGTTTCAGACCTTTCAACCACTAGCACT----TTGCAACATCTCCCACTAAACCCTATACATCATCTTCTCCT
GAAATGTTTCAGACCTTTCAACCACTAGCACTA-----GCAACATCTCCCACTAAACCCTATACATCATCTTCTCCT
GAAATGTTTCAGACCTTTCAACCACTAGCAC------TGCAACATCTCCCACTAAACCCTATACATCATCTTCTCCT
GAAATGTTTCAGACCTTTCAACCACTAGCA--------------TCTCCCACTAAACCCTATACATCATCTTCTCCT
GAAATGTTTCAGACCTTTCAACCACTAG--------------------CCACTAAACCCTATACATCATCTTCTCCT
GAAATGTTTCAGACCTTTCAACCACTA---------------------------AACCCTATACATCATCTTCTCCT
GAAATGTTTCAGACCTTTCAACCACTAG----------------------------CCCTATACATCATCTTCTCCT
HkCas12a
GAAATGTTTCAGACCTTTCAACCACTAGCACTAGC----CTTGCAACATCTCCCACTAAACCCTATACATCATCTTC
GAAATGTTTCAGACCTTTCAACCACTAGCACT----------GCAACATCTCCCACTAAACCCTATACATCATCTTC
GAAATGTTTCAGACCTTTCAACCACTAGCACTAGCaagcCTTGCAACATCTCCCACTAAACCCTATACATCATCTTC
GAAATGTTTCAGACCTTTCAACCACTAGCACTA--------TGCAACATCTCCCACTAAACCCTATACATCATCTTC
GAAATGTTTCAGACCTTTCAACCACTAGCA---------CTTGCAACATCTCCCACTAAACCCTATACATCATCTTC
GAAATGTTTCAGACCTTTCAACCACTAGCACTAGC----------ACATCTCCCACTAAACCCTATACATCATCTTC
GAAATGTTTCAGACCTTTCAACCACTAGCACTA------------ACATCTCCCACTAAACCCTATACATCATCTTC
GAAATGTTTCAGACCTTTCAACCACTAGCACTA------------------------AAACCCTATACATCATCTTC
LpCas12a
GAAATGTTTCAGACCTTTCAACCACTAGCACTAGCCTTGCAACATCTCCCACTAAACCCTATACATCATCTTCTCCT
GAAATGTTTCAGACCTTTCAACCACTAGCACTAGC----CAACATCTCCCACTAAACCCTATACATCATCTTCTCCT
GAAATGTTTCAGACCTTTCAACCACTAGCACTAGCTTG----CATCTCCCACTAAACCCTATACATCATCTTCTCCT
GAAATGTTTCAGACCTTTCAACCACTAGCACTAGC-----AACATCTCCCACTAAACCCTATACATCATCTTCTCCT
GAAATGTTTCAGACCTTTCAACCACTAG---------------------CACTAAACCCTATACATCATCTTCTCCT
--------------------------(Δ 80 bp)-----------------------CTATACATCATCTTCTCCT
--------------(Δ 83 bp)-----------------AACATCTCCCACTAAACCCTATACATCATCTTCTCCT
PrCas12a
GAAATGTTTCAGACCTTTCAACCACTAGCACTAGCCTTGCAACATCTCCCACTAAACCCTATACATCATCTTCTCCT
GAAATGTTTCAGACCTTTCAACCACTAGCACTAGC--TGCAACATCTCCCACTAAACCCTATACATCATCTTCTCCT
GAAATGTTTCAGACCTTTCAACCACTAGCACTAGCCTTGCA--ATCTCCCACTAAACCCTATACATCATCTTCTCCT
GAAATGTTTCAGACCTTTCAACCACTAGCACTA----TGCAACATCTCCCACTAAACCCTATACATCATCTTCTCCT
GAAATGTTTCAGACCTTTCAACCACTAGCACTA---------aATCTCCCACTAAACCCTATACATCATCTTCTCCT
GAAATGTTTCAGACCTTTCAAC---------------------------CACTAAACCCTATACATCATCTTCTCCT
```

f

Mouse *Nrl* taregt 1

```
ArCas12a
CTCAGTCCCAGAATGGCTTTCCCTCCCAGTCCCTTGGCT-ATGGAATATGTTAATGACTTTGATTTGATGAAGTTCG
CTCAGTCCCAGAATGGCTTTCCCTCCCAGTCCCTTGGCTaATGGAATATGTTAATGACTTTGATTTGATGAAGTTCG
CTCAGTCCCAGAATGGCTTTCCCTCCCAGTCCCTT-GCT-ATGGAATATGTTAATGACTTTGATTTGATGAAGTTCG
CTCAGTCCCAGAATGGCTTTCCCTCCCAGTCCCTT---T-ATGGAATATGTTAATGACTTTGATTTGATGAAGTTCG
CTCAGTCCCAGAATGGCTTTCCCTCCCAGTCCCT----------gATATGTTAATGACTTTGATTTGATGAAGTTCG
CTCAGTCCCAGAATGGCTTTCCCTCCCAGTCCCTT------------tTGTTAATGACTTTGATTTGATGAAGTTCG
BsCas12a
CTCAGTCCCAGAATGGCTTTCCCTCCCAGTCCCTTGGCTATGGAATATGTTAATGACTTTGATTTGATGAAGTTCGA
CTCAGTCCCAGAATGGCTTTCCCTCCCAGTCC-----CTATGGAATATGTTAATGACTTTGATTTGATGAAGTTCGA
CTCAGTCCCAGAATGGCTTTCCCTCCCAGTCCCT---------AATATGTTAATGACTTTGATTTGATGAAGTTCGA
CTCAGTCCCAGAATGGCTTTCCCTCCCAGTCCCTT----------aATGTTAATGACTTTGATTTGATGAAGTTCGA
CTCAGTCCCAGAATGGCTTTCC---------------------------------ACTTTGATTTGATGAAGTTCGA
CTCAGTCCCAGAATGGCTTT----------------------------------------GATTTGATGAAGTTCGA
CTCAGTCCCAGAATGGCTTT----------------------------------------ttTTTGATGAAGTTCGA
HkCas12a
CTCAGTCCCAGAATGGCTTTCCCTCCCAGTCCCTTGGCTATGGAATATGTTAATGACTTTGATTTGATGAAGTTCGA
CTCAGTCCCAGAATGGCTTTCCCTCCCAGTCCCTTGGCT----AATATGTTAATGACTTTGATTTGATGAAGTTCGA
CTCAGTCCCAGAATGGCTTTCCCTCCCAGTCCC------ATGGAATATGTTAATGACTTTGATTTGATGAAGTTCGA
CTCAGTCCCAGAATGGCTTTCCCTCCCAGTCCCTTGGC-----------TTAATGACTTTGATTTGATGAAGTTCGA
CTCAGTCCCAGAATGGCTTTCCCTCCCAGTCCCTT-----------ATGTTAATGACTTTGATTTGATGAAGTTCGA
LpCas12a
CTCAGTCCCAGAATGGCTTTCCCTCCCAGTCCCTTGGCTATGGAATATGTTAATGACTTTGATTTGATGAAGTTCGA
CTCAGTCCCAGAATGGCTTTCCCTCCCAGTCCCTT-----TGGAATATGTTAATGACTTTGATTTGATGAAGTTCGA
CTCAGTCCCAGAATGGCTTTCCCTCCCAGTCCCT-------------TGTTAATGACTTTGATTTGATGAAGTTCGA
CTCAGTCCCAGAATGGCTTTCCCTCCCAGTC---------------------ATGACTTTGATTTGATGAAGTTCGA
PrCas12a
CTCAGTCCCAGAATGGCTTTCCCTCCCAGTCCCTTGGCTATGGAATATGTTAATGACTTTGATTTGATGAAGTTCGA
CTCAGTCCCAGAATGGCTTTCCCTCCCAGTCCCT---------AATATGTTAATGACTTTGATTTGATGAAGTTCGA
CTCAGTCCCAGAATGGCTTTCCCTCCCAGTCCC--------------aGTTAATGACTTTGATTTGATGAAGTTCGA
CTCAGTCCCAGAATGGCTTTCCCTCCCAGTCCCTT--------------TTAATGACTTTGATTTGATGAAGTTCGA
CTCAGTCCCAGAAT-----------------------------------TTAATGACTTTGATTTGATGAAGTTCGA
CTCAGTCCCAGAATG-----------------------------------------CTTTGATTTGATGAAGTTCGA
CTCAGTCCCAGAATGGCTTTCCCTCCC-------------------------------------------AGTTCGA
PxCas12a
CTCAGTCCCAGAATGGCTTTCCCTCCCAGTCCCTTGGCTATGGAATATGTTAATGACTTTGATTTGATGAAGTTCGA
CTCAGTCCCAGAATGGCTTTCCCTCCCAGTCCCT----TATGGAATATGTTAATGACTTTGATTTGATGAAGTTCGA
CTCAGTCCCAGAATGGCTTTCCCTCCCAGTCCCTT--------AATATGTTAATGACTTTGATTTGATGAAGTTCGA
CTCAGTCCCAGAATGGCTTTCCCTCCCAGTCCC----------AATATGTTAATGACTTTGATTTGATGAAGTTCGA
CTCAGTCCCAGAATGGCTTTCCCTCCCAGTCCCTTGGCT------------------------------AAGTTCGA
```

Figure S3. Six new Cas12a proteins mediated robust genome editing in mammalian cells. **a** (*Upper*) Diagram of Cas12a expression vector driven by the CAG promoter. (*Lower*) Immunofluorescence staining of the tested 12 HA-tagged Cas12a proteins in human HeLa cells. NLS, nuclear localization signal. Scale bars, 20 µm. **b** (*Left*) Schematic showing the sequence of crRNAs targeting one human and two mouse gene sites with the requisite 5'-TTTN PAM. (*Right*) T7EI analysis of targeted indel frequencies generated by the six Cas12a orthologs as indicated. AsCas12a was used as positive control. GFP, an empty backbone vector without Cas12a protein expression. M, DNA marker. **c** (*Left*) Schematic showing the sequence of crRNA targeting one human and one mouse gene sites with the requisite 5'-TTN PAM. (*Right*) T7EI analysis of targeted indel frequencies generated by the six Cas12a orthologs and AsCas12a as indicated. GFP, an empty backbone vector without Cas12a protein expression. M, DNA marker. **d-f** Representative targeted human *CD34* sequences observed in **Figure 1b** and **Figure S3b**, and targted mouse *Nrl* sequences observed in **Figure S3c**. The PAM sequences were colored red. Green dashes and blue lowercases indicated deletions and insertions, respectively.

a

HkCas12a

Human *AAVS1* target site 1

GTGGAAAACTCCCTTTGTGAGAATGGTGCGTCC---TAGGTGTTCACCAGGTCGTGGCCGCCTCTACTCCCTTTCTC
GTGGAAAACTCCCTTTGTGAGAATGGTGCGTCCctaTAGGTGTTCACCAGGTCGTGGCCGCCTCTACTCCCTTTCTC
GTGGAAAACTCCCTTTGTGAGAATGGTGCGcac---acccccatttCCAGGTCGTGGCCGCCTCTACTCCCTTTCTC
GTGGAAAACTCCCTTTGTGAGAATGGTGCGTC----TAGGTGTTCACCAGGTCGTGGCCGCCTCTACTCCCTTTCTC
GTGGAAAACTCCCTTTGTGAGAATGGTGCG--------GGTGTTCACCAGGTCGTGGCCGCCTCTACTCCCTTTCTC

Human *AAVS1* target site 2

GTGGAAAACTCCCTTTGTGAGAATGGTGCGTCCTAGGTGTTCACCAGGTCGTGGCCGCCTCTACTCCCTTTCTCTTT
GTGGAAAACTCCCTTTGTGAGAATGGTGCGTCC--GGTGTTCACCAGGTCGTGGCCGCCTCTACTCCCTTTCTCTTT
GTGGAAAACTCCCTTTGTGAGAATGGTGCG-----------------------------TCTACTCCCTTTCTCTTT

Human *AAVS1* target site 3

TGCGTCCTAGGTGTTCACCAGGTCGTGGCCGCCTCTACTCCCTTTCTCTTTCTCCATCCTTCTTTCCTTAAAGAGTC
TGCGTCCTAGGTGTTCACCAGGTCGTGGCCGCCTCTACT-CCTTTCTCTTTCTCCATCCTTCTTTCCTTAAAGAGTC
TGCGTCCTAGGTGTTCACCAGGTCGTGGCCGCCT--ACTCCCTTTCTCTTTCTCCATCCTTCTTTCCTTAAAGAGTC
TGCGTCCTAGGTGTTCACCAGGTCGTGGCCGCCTC-----CCTTTCTCTTTCTCCATCCTTCTTTCCTTAAAGAGTC

Human *AAVS1* target site 4

AGGAGGGGGGTGTCCGTGTGGAAAACTCCCTTT----------------------------GTGAGAATGGTGCGTC
AGGAGGGGGGTGTCCGTGTGGAAAACTCCCTTTtgttcgccagttaatagtttgcgcaacgGTGAGAATGGTGCGTC
AGGAGGGGGGTGTCCGTGTGGAAAACTCCCTTT------------------------------GAGAATGGTGCGTC
AGGAGGGGGGTGTCCGTGTGGAAAACT-------------------------------------acAATGGTGCGTC

Human *AAVS1* target site 5

GGCGCTCAGGCTTCCCTGTCCCCCTTCCTCGTCCAC--CATCTCATGCCCCTGGCTCTCCTGCCCCTTCCCTACAGG
GGCGCTCAGGCTTCCCTGTCCCCCTTCCTCGTCCACcaCATCTCATGCCCCTGGCTCTCCTGCCCCTTCCCTACAGG
GGCGCTCAGGCTTCCCTGTCCCCCTTCCTC--------------ATGCCCCTGGCTCTCCTGCCCCTTCCCTACAGG
GGCGCTCAGGCTTCCCTGTCCCCCTTCCTCG--------------TGCCCCTGGCTCTCCTGCCCCTTCCCTACAGG

Human *AAVS1* target site 6

TCCGTGTGGAAAACTCCCTTTGTGAGAATGGTGCGTCCTAGGTGTTCACCAGGTCGTGGCCGCCTCTACTCCCTTTC
TCCGTGTGGAAAACTCCCTTTGTGAGAAT--TGCGTCCTAGGTGTTCACCAGGTCGTGGCCGCCTCTACTCCCTTTC
TCCGTGTGGAAAACTCCCTTTGTGAGAATG-------CTAGGTGTTCACCAGGTCGTGGCCGCCTCTACTCCCTTTC
TCCGTGTGGAAAACTCCCTT------------GCGTCCTAGGTGTTCACCAGGTCGTGGCCGCCTCTACTCCCTTTC
TCCGTGTGGAAAACTCCCTTTGTGAGAAcg-------------GTTCACCAGGTCGTGGCCGCCTCTACTCCCTTTC
TCCGTGTGGAAAACTCCCTTTGTGA------------------GTTCACCAGGTCGTGGCCGCCTCTACTCCCTTTC

Human *AAVS1* target site 7

CTCTCCTGCCCCTTCCCTACAGGGGTTCCTGGCT-CTGCTCTTCAGACTGAGCCCCGTTCCCCTGCATCCCCGTTCC
CTCTCCTGCCCCTTCCCTACAGGGGTTCCTGGCTtCTGCTCTTCAGACTGAGCCCCGTTCCCCTGCATCCCCGTTCC
CTCTCCTGCCCCTTCCCTACAGGGGTTCCTG-------CTCTTCAGACTGAGCCCCGTTCCCCTGCATCCCCGTTCC
CTCTCCTGCCCCTTCCCTACAGGGGTTCCTG-------agCTTCAGACTGAGCCCCGTTCCCCTGCATCCCCGTTCC
CTCTCCTGCCCCTTCCCTACAGGGGTTCCTG--------------GACTGAGCCCCGTTCCCCTGCATCCCCGTTCC

HkCas12a

Human *CCR5* target site 1

GCTTGTCATGGTCATCTGCTACTCGGGAATCCTAAAAACTCTGCTTCGGTGTCGAAATGAGAAGAAGAGGCACAGGG
GCTTGTCATGGTCATCTGCTACTCGGGAATCCTAAAAA----GCTTCGGTGTCGAAATGAGAAGAAGAGGCACAGGG
GCTTGTCATGGTCATCTGCTACTCGGGAATCCTAA------TGCTTCGGTGTCGAAATGAGAAGAAGAGGCACAGGG
GCTTGTCATGGTCATCTGCTACTCGGGAATCCTA-------TGCTTCGGTGTCGAAATGAGAAGAAGAGGCACAGGG
GCTTGTCATGGTCATCTGCTACTCGGGAATCCTAA----------TCGGTGTCGAAATGAGAAGAAGAGGCACAGGG

Human *CCR5* target site 2

TGTTTATTTTCTCTTCTGGGCTCCCTACAACATTGTCCTTCTCCTGAACACCTTCCAGGAATTCTTTGGCCTGAATA
TGTTTATTTTCTCTTCTGGGCTCCCTACAACATTGT--aTCTCCTGAACACCTTCCAGGAATTCTTTGGCCTGAATA
TGTTTATTTTCTCTTCTGGGCTCCCTACAACATTGT----CTCCTGAACACCTTCCAGGAATTCTTTGGCCTGAATA
TGTTTATTTTCTCTTCTGGGCTCCCTACAACATTG------TCCTGAACACCTTCCAGGAATTCTTTGGCCTGAATA
TGTTTATTTTCTCTTCTGGGCTCCCTACAACATTG-------gCTGAACACCTTCCAGGAATTCTTTGGCCTGAATA
TGTTTATTTTCTCTTCTGGGCTCCCTACA--------------CTGAACACCTTCCAGGAATTCTTTGGCCTGAATA

b

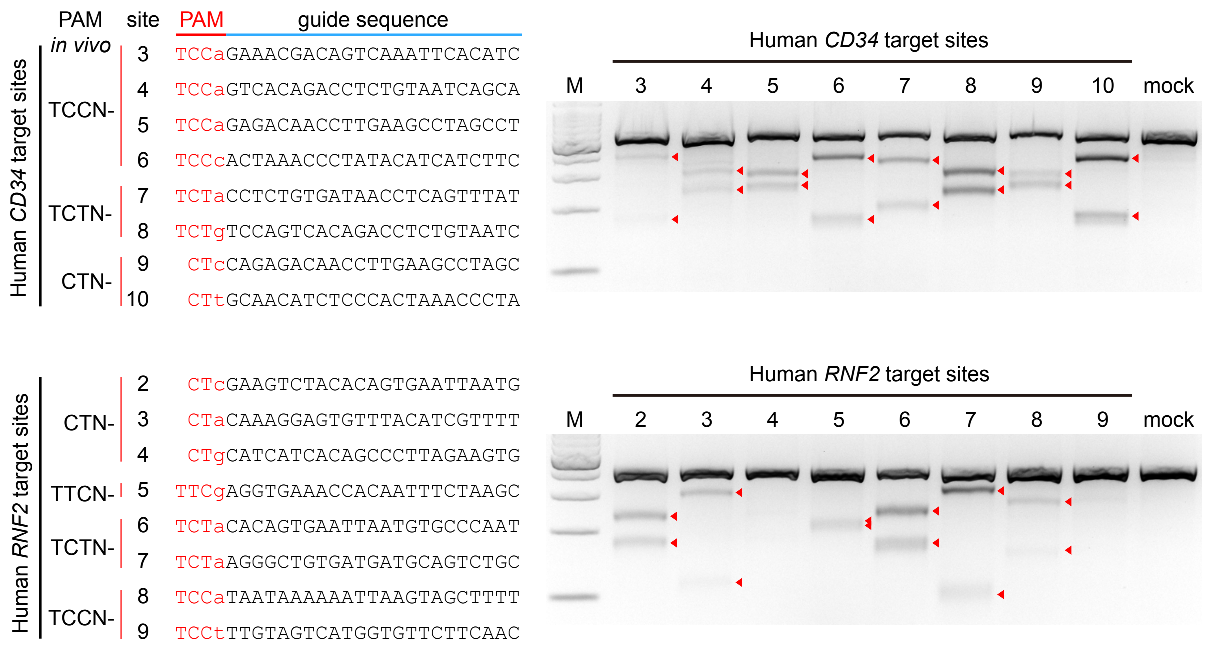

c

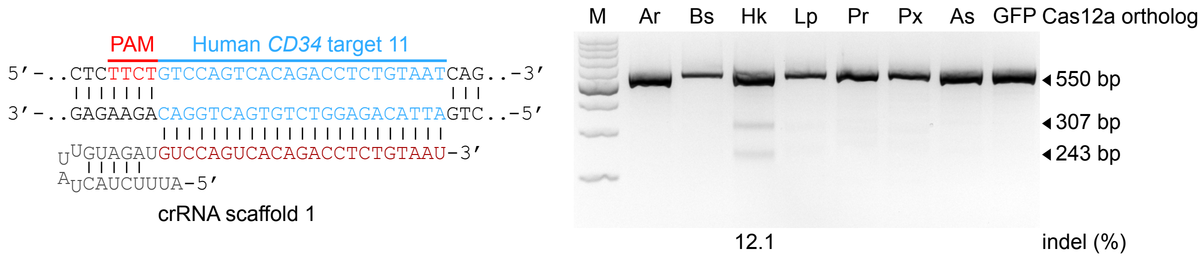

Figure S4. Increased genome-wide coverage of HkCas12a with altered PAMs. **a** Representative sequences of mutated alleles in the human *AAVS1* and *CCR5* loci indicated HkCas12a-crRNA1 edited the human genome recognizing 5'-YTN, 5'-TYN, 5'-TTYN and 5'-TCCN PAMs. The PAM sequences were colored red. Green dashes and blue lowercases indicated deletions and insertions, respectively. **b** (*Left*) Cas12a target sequences with indicated PAM sequences in the human *CD34*, *RNF2* and *CCR5* loci. (*Right*) T7EI analysis indicated that HkCas12a-crRNA1 targeted the human genome recognizing 5'-CTN and 5'-TYYN PAMs. Red triangles indicate cleavage bands. mock, an U6 empty vector without crRNA expression. M, DNA marker. **c** (*Left*) Schematic showing the crRNA targeting human *CD34* site 11. (*Right*) T7EI assay indicated that HkCas12a induced targeted indels directed by 5'-TTCN PAM. GFP, an empty backbone vector without Cas12a protein expression. M, DNA marker.

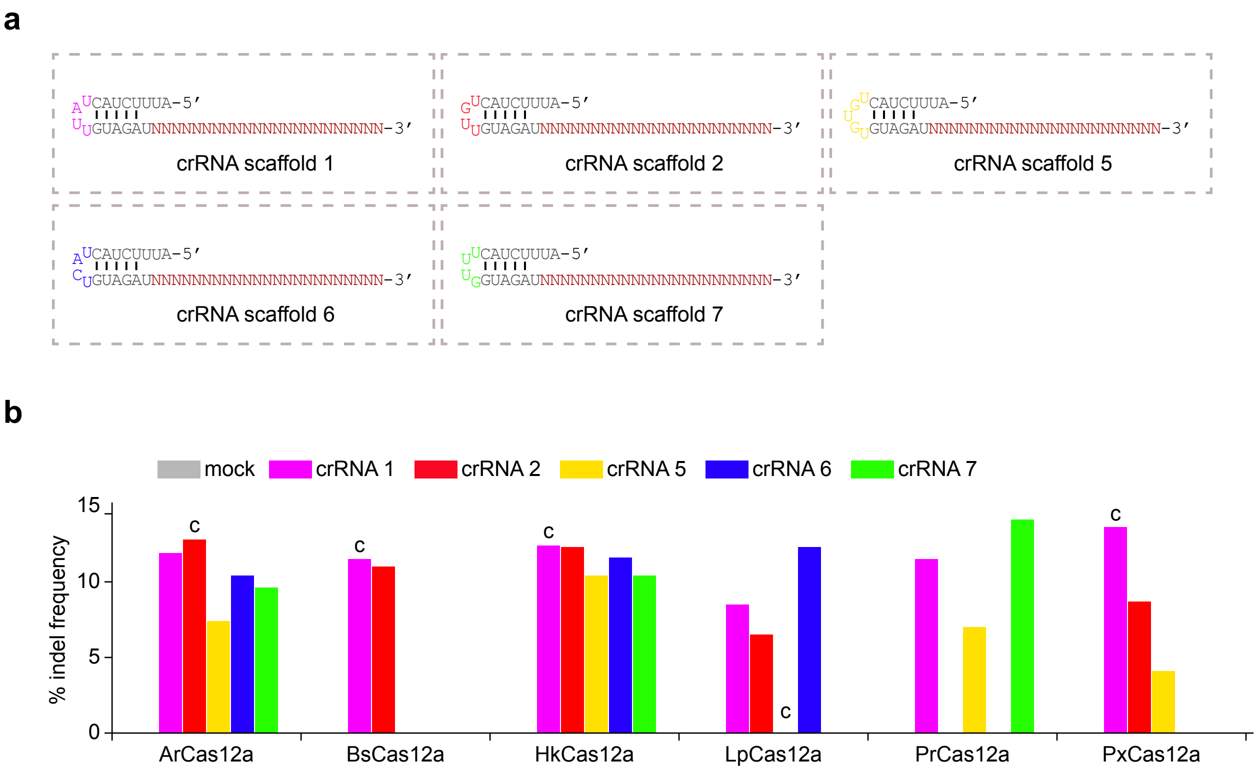

Figure S5. crRNA scaffold alters targeted indel efficiencies of Cas12a proteins. **a** Schematic showing the five crRNA scaffolds. Scaffold 1, 2 and 5 cover all cases of naturally existed crRNA scaffolds in this study, while scaffold 6 and 7 are two artifacts not found in nature. **b** Efficiencies of targeted indel mutations in the *MeCP2* locus in mES cells mediated by the five crRNA scaffolds with individual Cas12a-family protein were calculated by T7EI assay. Cognate crRNA was indicated with a “c” on the column. mock, an U6 empty vector without crRNA expression.

a

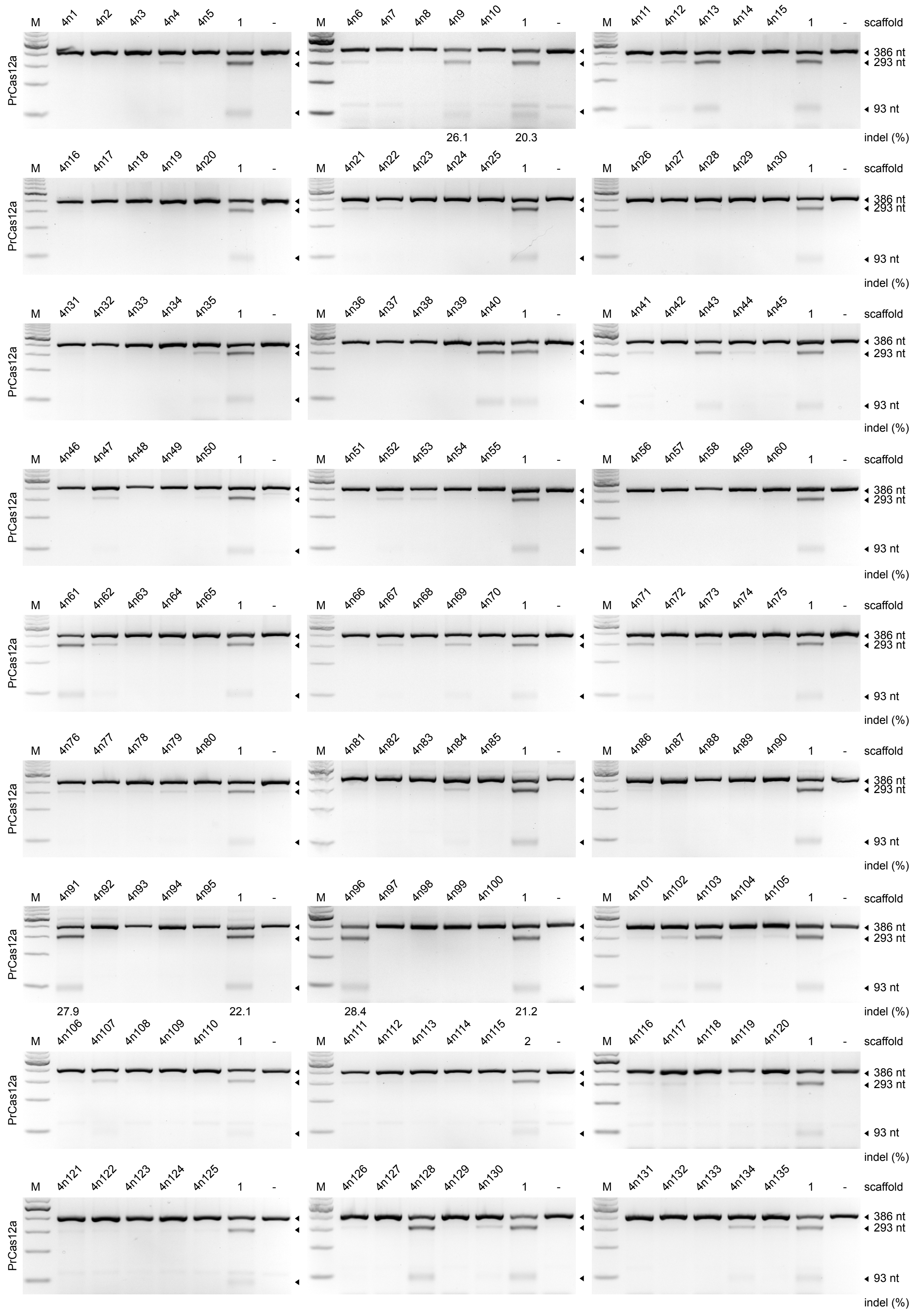
M 4n1 4n2 4n3 4n4 4n5 1 - M 4n6 4n7 4n8 4n9 4n10 1 - M 4n11 4n12 4n13 4n14 4n15 1 - scaffold
PrCas12a
386 nt
293 nt
93 nt
26.1 20.3
indel (%)
M 4n16 4n17 4n18 4n19 4n20 1 - M 4n21 4n22 4n23 4n24 4n25 1 - M 4n26 4n27 4n28 4n29 4n30 1 - scaffold
PrCas12a
386 nt
293 nt
93 nt
indel (%)
M 4n31 4n32 4n33 4n34 4n35 1 - M 4n36 4n37 4n38 4n39 4n40 1 - M 4n41 4n42 4n43 4n44 4n45 1 - scaffold
PrCas12a
386 nt
293 nt
93 nt
indel (%)
M 4n46 4n47 4n48 4n49 4n50 1 - M 4n51 4n52 4n53 4n54 4n55 1 - M 4n56 4n57 4n58 4n59 4n60 1 - scaffold
PrCas12a
386 nt
293 nt
93 nt
indel (%)
M 4n61 4n62 4n63 4n64 4n65 1 - M 4n66 4n67 4n68 4n69 4n70 1 - M 4n71 4n72 4n73 4n74 4n75 1 - scaffold
PrCas12a
386 nt
293 nt
93 nt
indel (%)
M 4n76 4n77 4n78 4n79 4n80 1 - M 4n81 4n82 4n83 4n84 4n85 1 - M 4n86 4n87 4n88 4n89 4n90 1 - scaffold
PrCas12a
386 nt
293 nt
93 nt
indel (%)
M 4n91 4n92 4n93 4n94 4n95 1 - M 4n96 4n97 4n98 4n99 4n100 1 - M 4n101 4n102 4n103 4n104 4n105 1 - scaffold
PrCas12a
386 nt
293 nt
93 nt
27.9 22.1 28.4 21.2
indel (%)
M 4n106 4n107 4n108 4n109 4n110 1 - M 4n111 4n112 4n113 4n114 4n115 2 - M 4n116 4n117 4n118 4n119 4n120 1 - scaffold
PrCas12a
386 nt
293 nt
93 nt
indel (%)
M 4n121 4n122 4n123 4n124 4n125 1 - M 4n126 4n127 4n128 4n129 4n130 1 - M 4n131 4n132 4n133 4n134 4n135 1 - scaffold
PrCas12a
386 nt
293 nt
93 nt
indel (%)

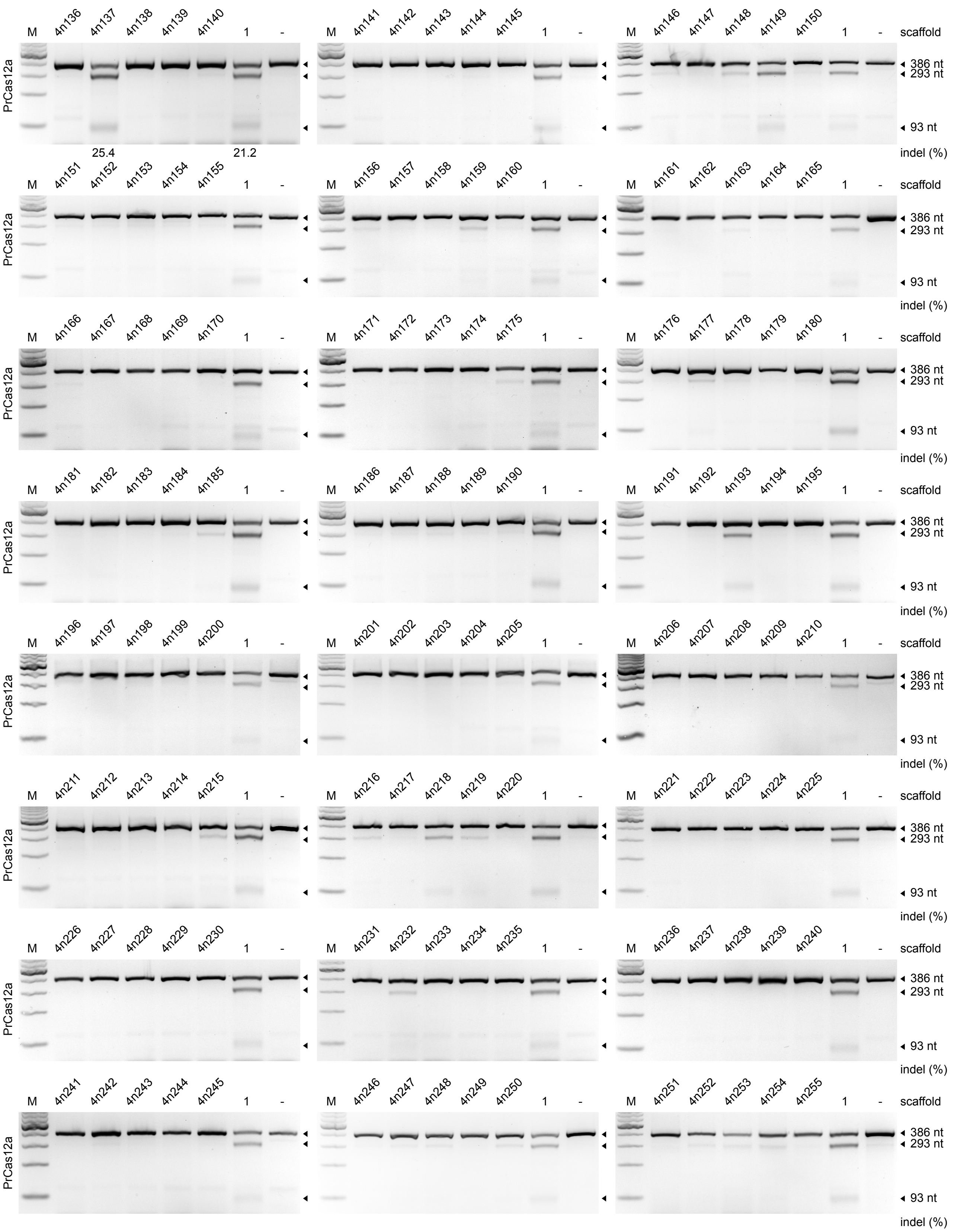

M 4n136 4n137 4n138 4n139 4n140 1 -
M 4n141 4n142 4n143 4n144 4n145 1 -
M 4n146 4n147 4n148 4n149 4n150 1 - scaffold
PrCas12a
386 nt
293 nt
93 nt
25.4
21.2
indel (%)
M 4n151 4n152 4n153 4n154 4n155 1 -
M 4n156 4n157 4n158 4n159 4n160 1 -
M 4n161 4n162 4n163 4n164 4n165 1 - scaffold
PrCas12a
386 nt
293 nt
93 nt
indel (%)
M 4n166 4n167 4n168 4n169 4n170 1 -
M 4n171 4n172 4n173 4n174 4n175 1 -
M 4n176 4n177 4n178 4n179 4n180 1 - scaffold
PrCas12a
386 nt
293 nt
93 nt
indel (%)
M 4n181 4n182 4n183 4n184 4n185 1 -
M 4n186 4n187 4n188 4n189 4n190 1 -
M 4n191 4n192 4n193 4n194 4n195 1 - scaffold
PrCas12a
386 nt
293 nt
93 nt
indel (%)
M 4n196 4n197 4n198 4n199 4n200 1 -
M 4n201 4n202 4n203 4n204 4n205 1 -
M 4n206 4n207 4n208 4n209 4n210 1 - scaffold
PrCas12a
386 nt
293 nt
93 nt
indel (%)
M 4n211 4n212 4n213 4n214 4n215 1 -
M 4n216 4n217 4n218 4n219 4n220 1 -
M 4n221 4n222 4n223 4n224 4n225 1 - scaffold
PrCas12a
386 nt
293 nt
93 nt
indel (%)
M 4n226 4n227 4n228 4n229 4n230 1 -
M 4n231 4n232 4n233 4n234 4n235 1 -
M 4n236 4n237 4n238 4n239 4n240 1 - scaffold
PrCas12a
386 nt
293 nt
93 nt
indel (%)
M 4n241 4n242 4n243 4n244 4n245 1 -
M 4n246 4n247 4n248 4n249 4n250 1 -
M 4n251 4n252 4n253 4n254 4n255 1 - scaffold
PrCas12a
386 nt
293 nt
93 nt
indel (%)

b

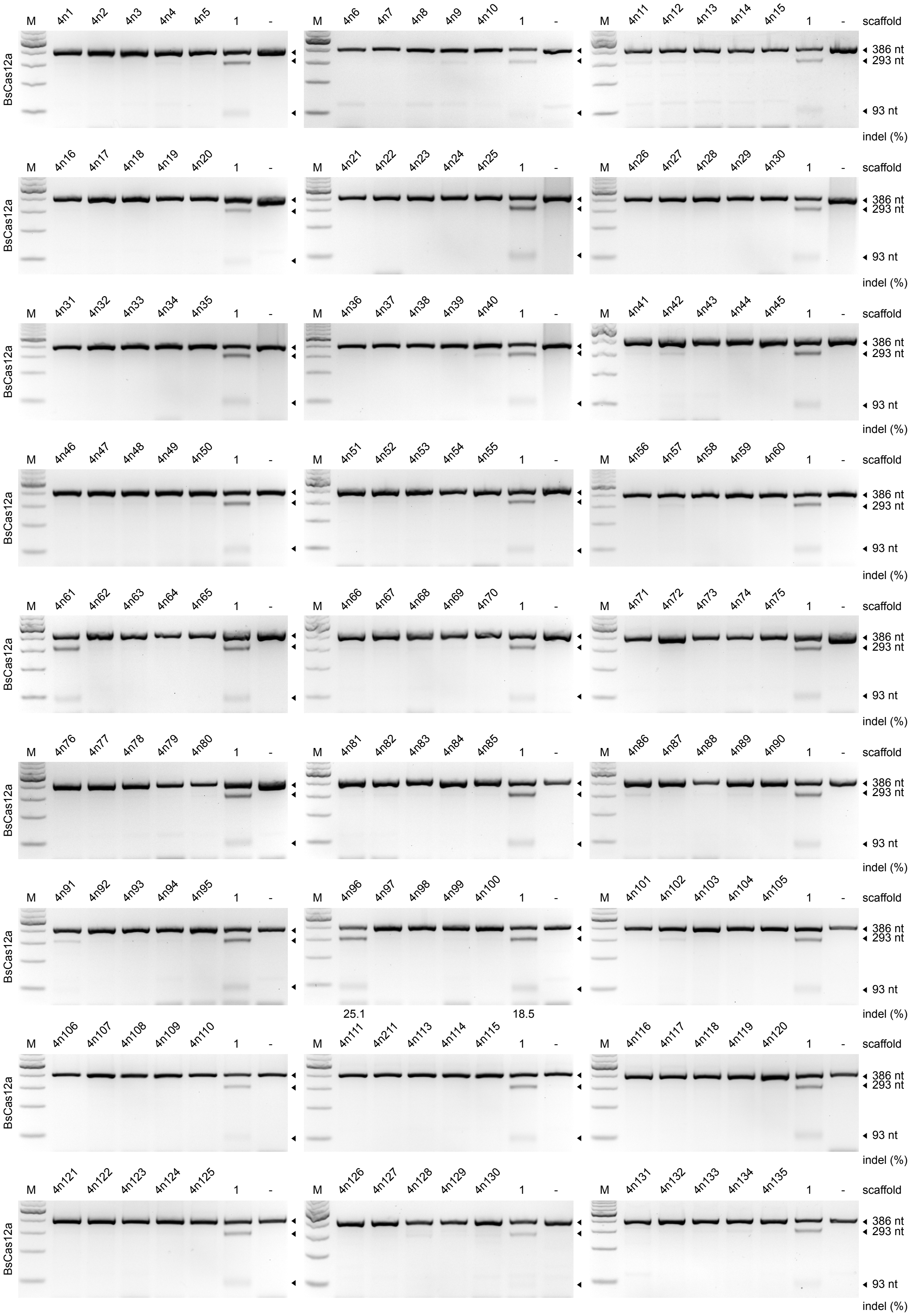
M 4n1 4n2 4n3 4n4 4n5 1 -
M 4n6 4n7 4n8 4n9 4n10 1 -
M 4n11 4n12 4n13 4n14 4n15 1 - scaffold
BsCas12a
386 nt
293 nt
93 nt
indel (%)
M 4n16 4n17 4n18 4n19 4n20 1 -
M 4n21 4n22 4n23 4n24 4n25 1 -
M 4n26 4n27 4n28 4n29 4n30 1 - scaffold
BsCas12a
386 nt
293 nt
93 nt
indel (%)
M 4n31 4n32 4n33 4n34 4n35 1 -
M 4n36 4n37 4n38 4n39 4n40 1 -
M 4n41 4n42 4n43 4n44 4n45 1 - scaffold
BsCas12a
386 nt
293 nt
93 nt
indel (%)
M 4n46 4n47 4n48 4n49 4n50 1 -
M 4n51 4n52 4n53 4n54 4n55 1 -
M 4n56 4n57 4n58 4n59 4n60 1 - scaffold
BsCas12a
386 nt
293 nt
93 nt
indel (%)
M 4n61 4n62 4n63 4n64 4n65 1 -
M 4n66 4n67 4n68 4n69 4n70 1 -
M 4n71 4n72 4n73 4n74 4n75 1 - scaffold
BsCas12a
386 nt
293 nt
93 nt
indel (%)
M 4n76 4n77 4n78 4n79 4n80 1 -
M 4n81 4n82 4n83 4n84 4n85 1 -
M 4n86 4n87 4n88 4n89 4n90 1 - scaffold
BsCas12a
386 nt
293 nt
93 nt
indel (%)
M 4n91 4n92 4n93 4n94 4n95 1 -
M 4n96 4n97 4n98 4n99 4n100 1 -
M 4n101 4n102 4n103 4n104 4n105 1 - scaffold
BsCas12a
386 nt
293 nt
93 nt
25.1
18.5
indel (%)
M 4n106 4n107 4n108 4n109 4n110 1 -
M 4n111 4n211 4n113 4n114 4n115 1 -
M 4n116 4n117 4n118 4n119 4n120 1 - scaffold
BsCas12a
386 nt
293 nt
93 nt
indel (%)
M 4n121 4n122 4n123 4n124 4n125 1 -
M 4n126 4n127 4n128 4n129 4n130 1 -
M 4n131 4n132 4n133 4n134 4n135 1 - scaffold
BsCas12a
386 nt
293 nt
93 nt
indel (%)

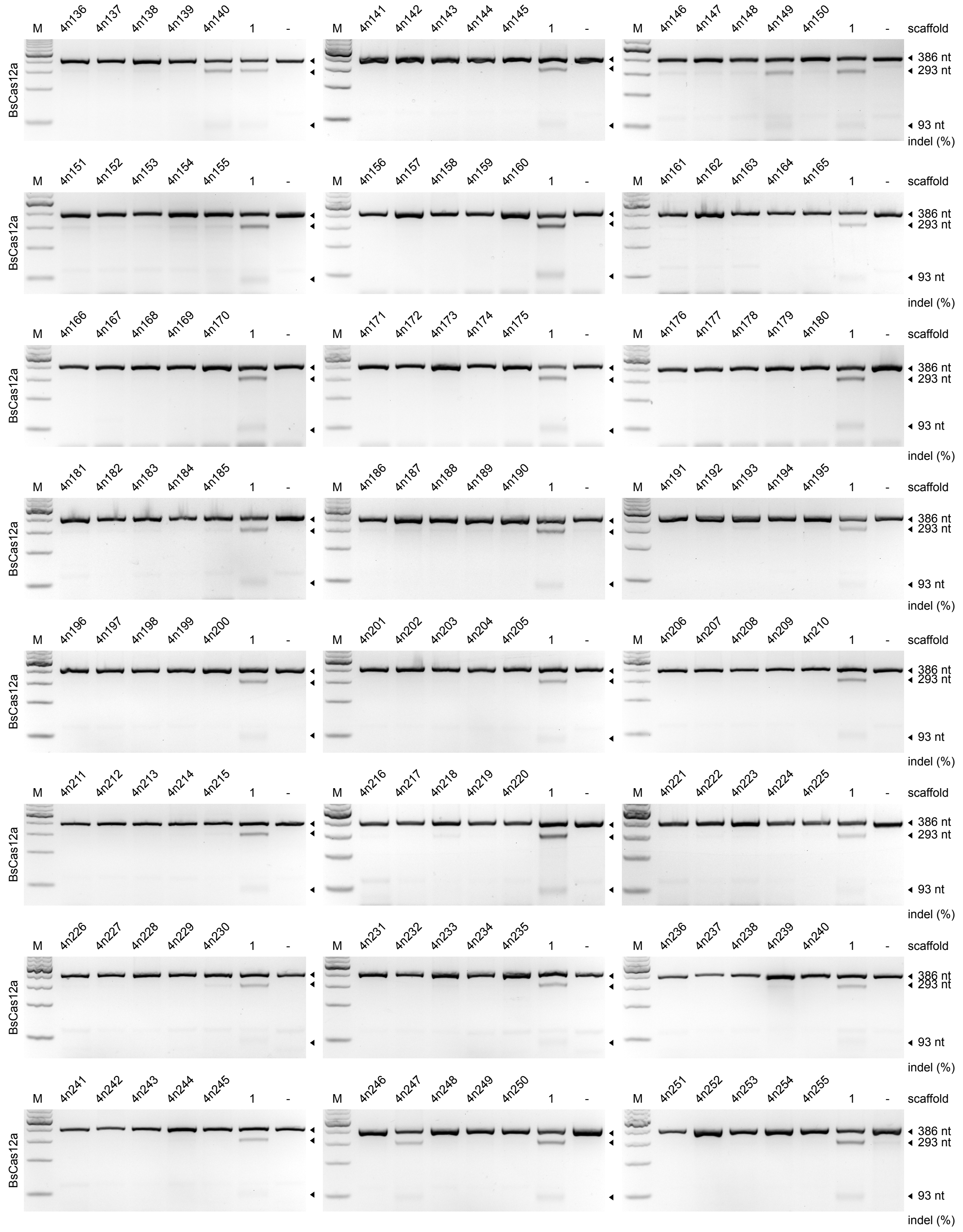

M
4n136
4n137
4n138
4n139
4n140
1
-
M
4n141
4n142
4n143
4n144
4n145
1
-
M
4n146
4n147
4n148
4n149
4n150
1
-
scaffold
BsCas12a
386 nt
293 nt
93 nt
indel (%)
4n151
4n152
4n153
4n154
4n155
4n156
4n157
4n158
4n159
4n160
4n161
4n162
4n163
4n164
4n165
4n166
4n167
4n168
4n169
4n170
4n171
4n172
4n173
4n174
4n175
4n176
4n177
4n178
4n179
4n180
4n181
4n182
4n183
4n184
4n185
4n186
4n187
4n188
4n189
4n190
4n191
4n192
4n193
4n194
4n195
4n196
4n197
4n198
4n199
4n200
4n201
4n202
4n203
4n204
4n205
4n206
4n207
4n208
4n209
4n210
4n211
4n212
4n213
4n214
4n215
4n216
4n217
4n218
4n219
4n220
4n221
4n222
4n223
4n224
4n225
4n226
4n227
4n228
4n229
4n230
4n231
4n232
4n233
4n234
4n235
4n236
4n237
4n238
4n239
4n240
4n241
4n242
4n243
4n244
4n245
4n246
4n247
4n248
4n249
4n250
4n251
4n252
4n253
4n254
4n255

c

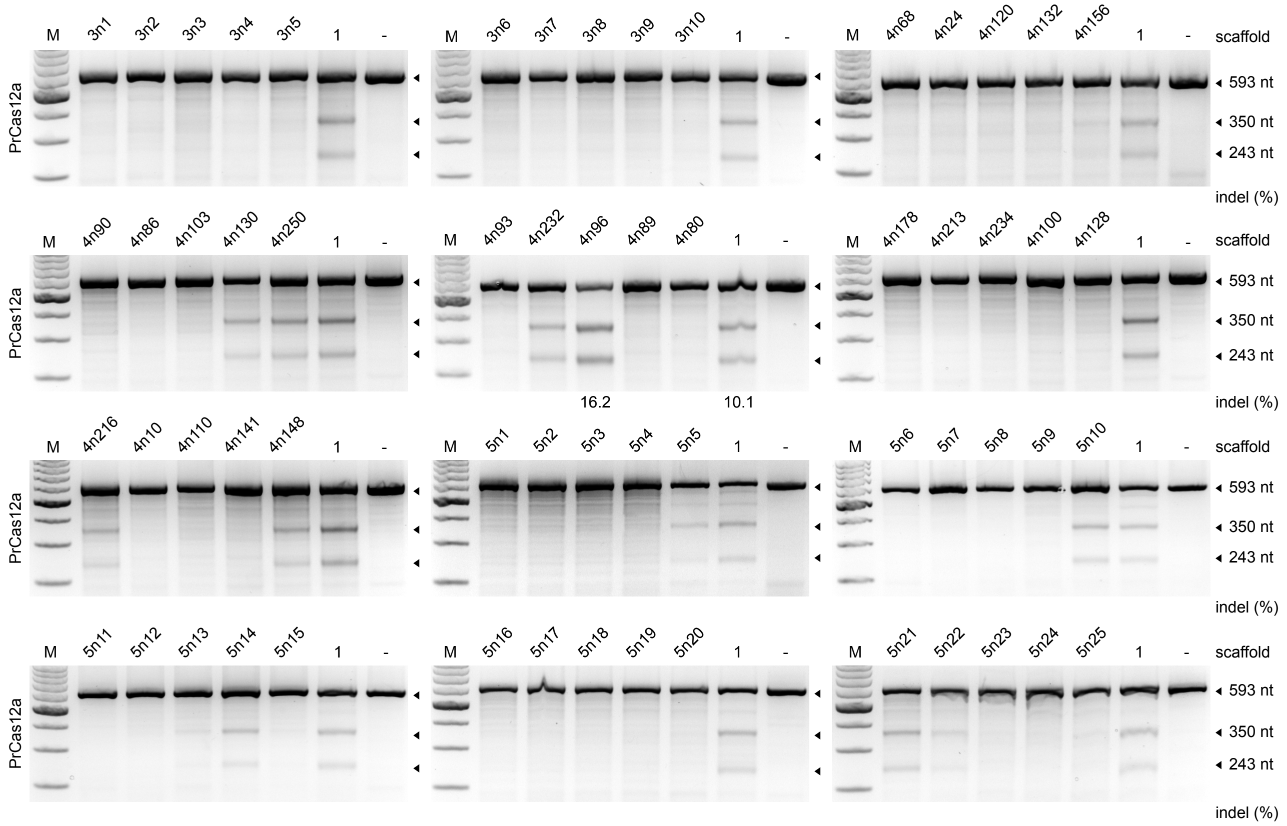
M 3n1 3n2 3n3 3n4 3n5 1 -
M 3n6 3n7 3n8 3n9 3n10 1 -
M 4n68 4n24 4n120 4n132 4n156 1 - scaffold
PrCas12a
593 nt
350 nt
243 nt
indel (%)
M 4n90 4n86 4n103 4n130 4n250 1 -
M 4n93 4n232 4n96 4n89 4n80 1 -
M 4n178 4n213 4n234 4n100 4n128 1 - scaffold
PrCas12a
593 nt
350 nt
243 nt
16.2
10.1
indel (%)
M 4n216 4n10 4n110 4n141 4n148 1 -
M 5n1 5n2 5n3 5n4 5n5 1 -
M 5n6 5n7 5n8 5n9 5n10 1 - scaffold
PrCas12a
593 nt
350 nt
243 nt
indel (%)
M 5n11 5n12 5n13 5n14 5n15 1 -
M 5n16 5n17 5n18 5n19 5n20 1 -
M 5n21 5n22 5n23 5n24 5n25 1 - scaffold
PrCas12a
593 nt
350 nt
243 nt
indel (%)

d

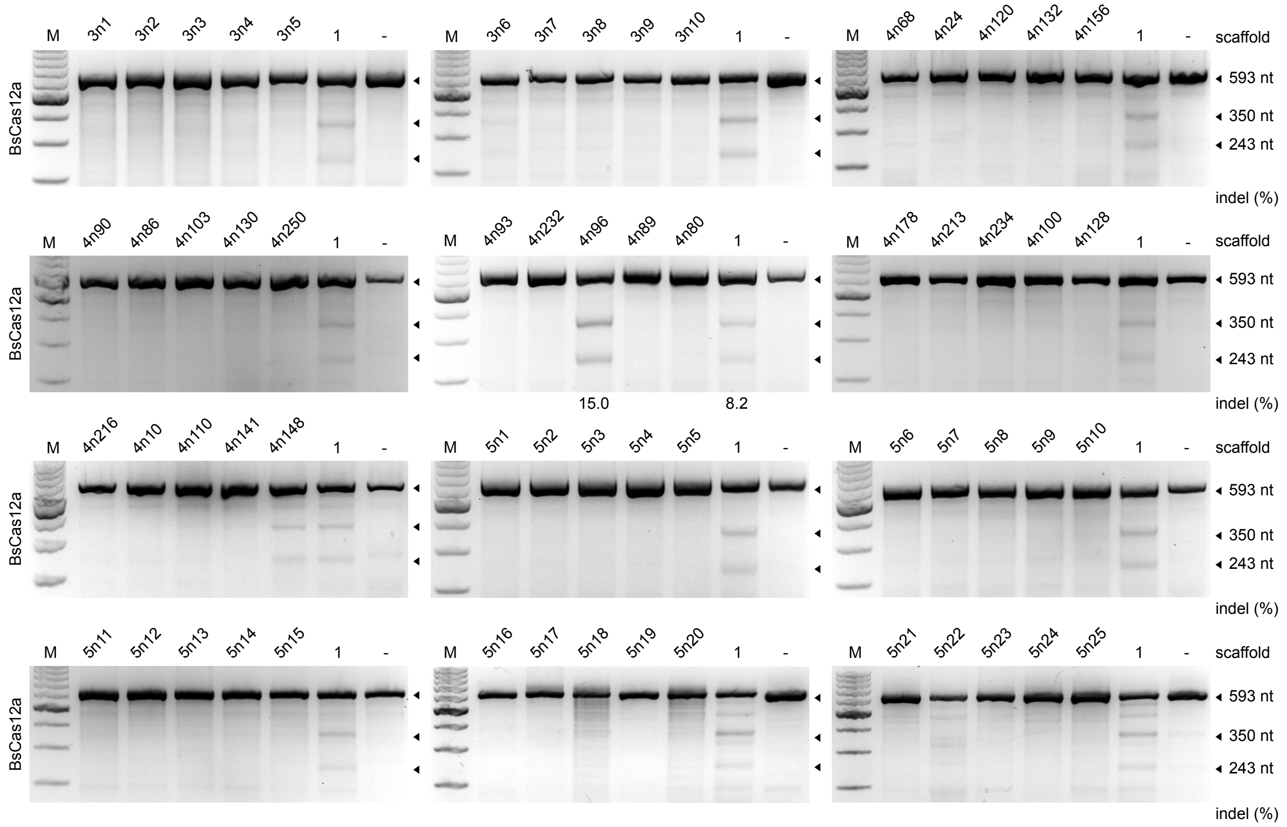
M 3n1 3n2 3n3 3n4 3n5 1 -
M 3n6 3n7 3n8 3n9 3n10 1 -
M 4n68 4n24 4n120 4n132 4n156 1 - scaffold
BsCas12a
593 nt
350 nt
243 nt
indel (%)
M 4n90 4n86 4n103 4n130 4n250 1 -
M 4n93 4n232 4n96 4n89 4n80 1 -
M 4n178 4n213 4n234 4n100 4n128 1 - scaffold
BsCas12a
593 nt
350 nt
243 nt
15.0
8.2
indel (%)
M 4n216 4n10 4n110 4n141 4n148 1 -
M 5n1 5n2 5n3 5n4 5n5 1 -
M 5n6 5n7 5n8 5n9 5n10 1 - scaffold
BsCas12a
593 nt
350 nt
243 nt
indel (%)
M 5n11 5n12 5n13 5n14 5n15 1 -
M 5n16 5n17 5n18 5n19 5n20 1 -
M 5n21 5n22 5n23 5n24 5n25 1 - scaffold
BsCas12a
593 nt
350 nt
243 nt
indel (%)

Figure S6. crRNA scaffold optimization and screening. **a-b** 256 crRNA scaffolds with all possible 4 nt-loop were used for genome editing screening with PrCas12a **(a)** and BsCas12a **(b)** in the *Nrl* locus in mES cells and T7EI assay was applied. Enhanced indels compared with scaffold 1 were labelled. -, an U6 empty vector without crRNA expression. M, DNA marker. **c-d** Target indels produced in the *MeCP2* locus in mES cells by PrCas12a **(c)** and BsCas12a **(d)** with crRNA scaffolds bearing mutations within 3 nt-, 4 nt- or 5 nt-loop. Enhanced indels compared with scaffold 1 were labelled. -, an U6 empty vector without crRNA expression. M, DNA marker.

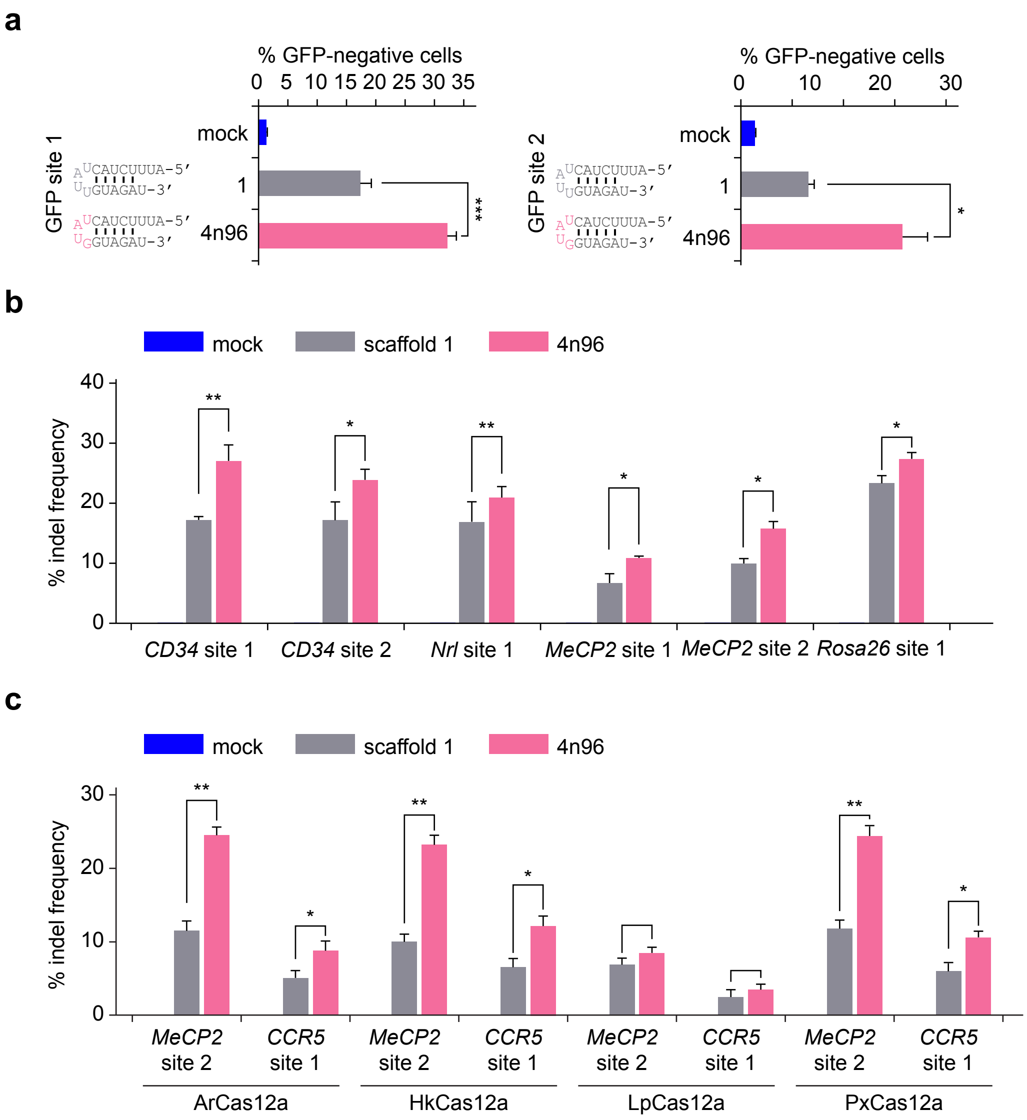

Figure S7. Enhanced targeted efficiency with optimized crRNA scaffold. **a** Efficiencies of GFP disruption in human 293FT cells mediated by BsCas12a with crRNA scaffold 4n96 and 1. Error bars indicate standard errors of the mean (s.e.m.), n = 3. * and *** mean p value < 0.05 and 0.001, respectively. mock, an U6 empty vector without crRNA expression. **b** T7EI analysis of targeted mutation efficiencies induced at six different endogenous loci by scaffold 4n96 and 1 combined with BsCas12a. Error bars indicate s.e.m., n = 3. * means p value < 0.05 and ** means p value < 0.01. **c** T7EI analysis of targeted mutation efficiencies induced at two different endogenous loci by scaffold 4n96 and 1 combined with individual Cas12a. Error bars indicate s.e.m., n = 3. * means p value < 0.05 and ** means p value < 0.01.

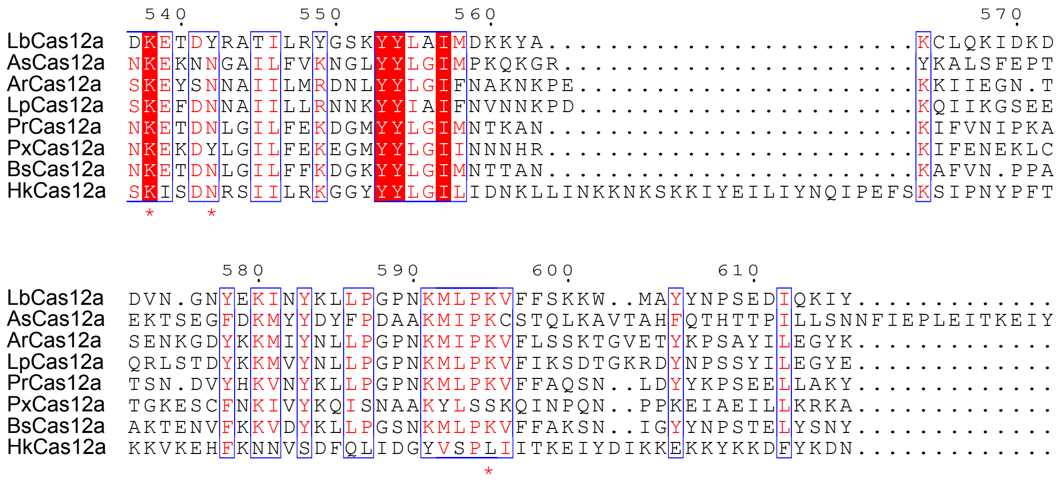

Figure S8. Conserved residues in PAM-interacting (PI) domain of Cas12a proteins. The three residues in the PI domain responsible for PAM recognition are indicated with red asterisk. In HkCas12a, L642 equivalent to K592 of LbCas12a, is not conserved.
