# Supplementary material for: Enhanced mammalian genome editing by new Cas12a orthologs with optimized crRNA scaffolds
Source: Genome Biol. 2019 Feb 5;20:15. doi: 10.1186/s13059-019-1620-8 (PMC6362571; doi:10.1186/s13059-019-1620-8)
Supplement: Supplementary file 4 — Table S2. Sequences of targeting crRNAs used for in vitro RNA transcription. Table S3. Target sequences harboring various 5′ PAM sequences used for in vitro DNA cleavage assay. Table S4. Protospacer sequences used for genome editing. Table S5. crRNA scaffold optimization and screening. Table S6. Primer sequences used for PCR amplification. Table S7. Frequency of Cas12a- and SpCas9-mediated targeted indel mutations at on-target sites in human 293FT cells. Table S8. Off-target analysis by targeted deep sequencing in the human 293FT cells. Table S9. Off-target analysis by whole genome sequencing (WGS) in human 293FT cells. (PDF 395 kb) [file 13059_2019_1620_MOESM4_ESM.pdf]

Table S2. Sequences of targeting crRNAs used for *in vitro* RNA transcription. Targeting crRNAs bear the 5' T7 promoter sequence. The purchased oligos were annealed to form dsDNA templates. Sequences of T7 promoter and crRNA scaffolds were highlighted in blue and red fonts, respectively.

crRNA ID	crRNA sequences (5′ - 3′)
crRNA1-F<br>crRNA1-R	TAATACGACTCACTATAGGATTTCTACTATTGTAGATAGGAGTGTTCAGTCTCCGTGAAC<br>GTTCACGGAGACTGAACACTCCTATCTACAATAGTAGAAATCCTATAGTGAGTCGTATTA
crRNA2-F<br>crRNA2-R	TAATACGACTCACTATAGGATTTCTACTGTTGTAGATAGGAGTGTTCAGTCTCCGTGAAC<br>GTTCACGGAGACTGAACACTCCTATCTACAACAGTAGAAATCCTATAGTGAGTCGTATTA
crRNA5-F<br>crRNA5-R	TAATACGACTCACTATAGGATTTCTACTGTGTGTAGATAGGAGTGTTCAGTCTCCGTGAAC<br>GTTCACGGAGACTGAACACTCCTATCTACACACAGTAGAAATCCTATAGTGAGTCGTATTA

Table S3. Target sequences harboring various 5' PAM sequences used for *in vitro* DNA cleavage assay. Synthesized oligos containing the protospacer targeting sequences and adjacent 5' PAMs were annealed and constructed into EcoRI and SphI double-digested p11-LacY-wtx1 vector. 5' EcoRI overhang and 3' SphI overhang were highlighted in yellow and green backgrounds, respectively. The 5' PAM sequences were colored red.

PAM ID	Target sequences (5′ to 3′)	5′PAM sequences
p11-PMA1-F	AATTATTTNAGGAGTGTTCAGTCTCCGTGAACCATG	TTTN
p11-PMA1-R	GTTCACGGAGACTGAACACTCCTNAAAT	
p11-PMA2-F	AATTAATTNAGGAGTGTTCAGTCTCCGTGAACCATG	ATTN
p11-PMA2-R	GTTCACGGAGACTGAACACTCCTNAATT	
p11-PMA3-F	AATTAGTTNAGGAGTGTTCAGTCTCCGTGAACCATG	GTTN
p11-PMA3-R	GTTCACGGAGACTGAACACTCCTNAACT	
p11-PMA4-F	AATTACTTNAGGAGTGTTCAGTCTCCGTGAACCATG	CTTN
p11-PMA4-R	GTTCACGGAGACTGAACACTCCTNAAGT	
p11-PMA5-F	AATTATTNAGGAGTGTTCAGTCTCCGTGAACCATG	TTN
p11-PMA5-R	GTTCACGGAGACTGAACACTCCTNAAT	
p11-PMA6-F	AATTATANAGGAGTGTTCAGTCTCCGTGAACCATG	TAN
p11-PMA6-R	GTTCACGGAGACTGAACACTCCTNTAT	
p11-PMA7-F	AATTATGNAGGAGTGTTCAGTCTCCGTGAACCATG	TGN
p11-PMA7-R	GTTCACGGAGACTGAACACTCCTNCAT	
p11-PMA8-F	AATTATCNAGGAGTGTTCAGTCTCCGTGAACCATG	TCN
p11-PMA8-R	GTTCACGGAGACTGAACACTCCTNGAT	
p11-PMA9-F	AATTAATNAGGAGTGTTCAGTCTCCGTGAACCATG	ATN
p11-PMA9-R	GTTCACGGAGACTGAACACTCCTNATT	
p11-PMA10-F	AATTAGTNAGGAGTGTTCAGTCTCCGTGAACCATG	GTN
p11-PMA10-R	GTTCACGGAGACTGAACACTCCTNACT	
p11-PMA11-F	AATTACTNAGGAGTGTTCAGTCTCCGTGAACCATG	CTN
p11-PMA11-R	GTTCACGGAGACTGAACACTCCTNAGT	
p11-PMA12-F	AATTAAANAGGAGTGTTCAGTCTCCGTGAACCATG	AAN
p11-PMA12-R	GTTCACGGAGACTGAACACTCCTNTTT	
p11-PMA13-F	AATTAGGNAGGAGTGTTCAGTCTCCGTGAACCATG	GGN
p11-PMA13-R	GTTCACGGAGACTGAACACTCCTNCCT	
p11-PMA14-F	AATTACCNAGGAGTGTTCAGTCTCCGTGAACCATG	CCN
p11-PMA14-R	GTTCACGGAGACTGAACACTCCTNGGT	
p11-PMA15-F	AATTATTGAGGAGTGTTCAGTCTCCGTGAACCATG	TTG
p11-PMA15-R	GTTCACGGAGACTGAACACTCCTCAAT	
p11-PMA16-F	AATTATTCAGGAGTGTTCAGTCTCCGTGAACCATG	TTC
p11-PMA16-R	GTTCACGGAGACTGAACACTCCTGAAT	

p11-PMA17-F	AATTATTAAGGAGTGTTCAGTCTCCGTGAACCATG	TTA
p11-PMA17-R	GTTCACGGAGACTGAACACTCCTCTAAT	
p11-PMA18-F	AATTATTTAGGAGTGTTCAGTCTCCGTGAACCATG	TTT
p11-PMA18-R	GTTCACGGAGACTGAACACTCCTAAAT	

Table S4. Protospacer sequences used for mammalian genome editing. Mammalian genome targeting protospacers were designed based on type V-A CRISPR-Cas12a or type II CRISPR-SpCas9 loci with their requisite PAMs.

Target species	Gene	Proto-spacer ID	Protospacer sequence (5' to 3')	Cell line tested
Homo sapiens	*AAVS1*	1	TTTGTGAGAATGGTGCGTCCTAGGTGT	293FT
		2	TTTGTGAGAATGGTGCGTCCTAGGTG	293FT
		3	TTCACCAGGTCGTGGCCGCCTCTACT	293FT
		4	TCCGTGTGGAAAACTCCCTTTGTGAGA	293FT
		5	TCCCTGTCCCCCTTCCTCGTCCACCAT	293FT
		6	CTCCCTTTGTGAGAATGGTGCGTCCT	293FT
		7	TTCCCTACAGGGGTTCCTGGCTCTGCT	293FT
	CD34	1	TTTCAACTCCAGAGACAACCTTGAAGC	293FT
		2	TTTCAACCACTAGCACTAGCCTTGCAA	293FT
		3	TCCAGAAACGACAGTCAAATTCACATC	293FT
		4	TCCAGTCACAGACCTCTGTAATCAGCA	293FT
		5	TCCAGAGACAACCTTGAAGCCTAGCCT	293FT
		6	TCCCACTAAACCCTATACATCATCTTC	293FT
		7	TCTACCTCTGTGATAACCTCAGTTTAT	293FT
		8	TCTGTCCAGTCACAGACCTCTGTAATC	293FT
		9	CTCCAGAGACAACCTTGAAGCCTAGC	293FT
		10	CTTGCAACATCTCCCACTAAACCCTA	293FT
		11	TTCTGTCCAGTCACAGACCTCTGTAAT	293FT
	RNF2	1	TTGGGCACATTAATTCACTGTGTAGA	293FT
		2	CTCGAAGTCTACACAGTGAATTAATG	293FT
		3	CTACAAAGGAGTGTTTACATCGTTTT	293FT
		4	CTGCATCATCACAGCCCTTAGAAGTG	293FT
		5	TTCGAGGTGAAACCACAATTTCTAAGC	293FT
		6	TCTACACAGTGAATTAATGTGCCCAAT	293FT
		7	TCTAAGGGCTGTGATGATGCAGTCTGC	293FT
		8	TCCATAATAAAAAATTAAGTAGCTTTT	293FT
		9	TCCTTTGTAGTCATGGTGTTCTTCAAC	293FT
	DNMT1	1	TTTCCCTTCAGCTAAAATAAAGGAGGA	293FT
		2	TTTATTTCCCTTCAGCTAAAATAAAGG	293FT
	CCR5	1	TTTGGCCTGAATAATTGCAGTAGCTCT	293FT
	EMX1	1	CCAATGACTAGGGTGGGCAACCACAAA	293FT
	DNMT1	1	CCCTTCAGCTAAAATAAAGGAGG	293FT
		2	TTTCCCTTCAGCTAAAATAAAGG	293FT
	CCR5	1	CCTGAATAATTGCAGTAGCTCTA	293FT

	EMX1	1	GTCACCTCCAATGACTAGGGTGG	293FT
Mus musculus	*MeCP2*	1	TTTGCCTGCCTCTGCTGGCTCTGCAGA	ESC
		2	TTTCGTGTCCAACCTTCAGGCAAGGTG	ESC
	Nrl	1	TTTCCCTCCCAGTCCCTTGGCTATGGA	ESC
		2	TTGGGCTCCACACCATACAGCTCGGT	ESC
	Rosa26	1	TTTGTCTCCTGAATACTAAGGTTAAAA	ESC

Table S5. crRNA scaffold optimization and screening. Sequences of crRNA scaffolds within the loop were optimized, and the rest sequences remained the same as crRNA scaffold 1. Totally, 256 crRNA scaffolds (including scaffold 1, 2, 6 and 7) with all the possible 4-nt loop sequences thoroughly covered were generated. Meanwhile, 10 crRNA scaffolds with the 3-nt loop sequence mutated, 25 crRNA scaffolds with the 4-nt loop sequence mutated and 25 crRNA scaffolds (including scaffold 5) with the 5-nt loop sequence mutated targeting the *MeCP2* gene site were generated for screening. Spacer sequences targeting mouse endogenous genes are in blue or green font.

crRNA scaffold ID	crRNA sequences containing spacer (5′ to 3′)
crRNA4n-*Nrl* Pool	TGCAATTTCTACNNNNGTAGATGTGTCCAACCTTCAGGCAAGGTG<br>TGTTCACCTTGCCTGAAGGTTGGACACATCTACNNNNGTAGAAAT
crRNA4n-*MeCP2* Pool	TGCAATTTCTACNNNNGTAGATCCTGCCTCTGCTGGCTCTGCAGA<br>TGTTTCTGCAGAGCCAGCAGAGGCAGGATCTACNNNNGTAGAAAT
crRNA1-*MeCP2*	ATTTCTACTATTGTAGATCCTGCCTCTGCTGGCTCTGCAGA
crRNA2-*MeCP2*	ATTTCTACTGTTGTAGATCCTGCCTCTGCTGGCTCTGCAGA
crRNA5-*MeCP2*	ATTTCTACTAGTTGTAGATCCTGCCTCTGCTGGCTCTGCAGA
crRNA6-*MeCP2*	ATTTCTACTACTGTAGATCCTGCCTCTGCTGGCTCTGCAGA
crRNA7-*MeCP2*	ATTTCTACTTTGGTAGATCCTGCCTCTGCTGGCTCTGCAGA
crRNA1-*Nrl*	ATTTCTACTATTGTAGATGTGTCCAACCTTCAGGCAAGGTG
crRNA4n1-*Nrl*	ATTTCTACGTACGTAGATGTGTCCAACCTTCAGGCAAGGTG
crRNA4n2-*Nrl*	ATTTCTACACGAGTAGATGTGTCCAACCTTCAGGCAAGGTG
crRNA4n3-*Nrl*	ATTTCTACGATAGTAGATGTGTCCAACCTTCAGGCAAGGTG
crRNA4n4-*Nrl*	ATTTCTACTGAAGTAGATGTGTCCAACCTTCAGGCAAGGTG
crRNA4n5-*Nrl*	ATTTCTACTGATGTAGATGTGTCCAACCTTCAGGCAAGGTG
crRNA4n6-*Nrl*	ATTTCTACTAGCGTAGATGTGTCCAACCTTCAGGCAAGGTG
crRNA4n7-*Nrl*	ATTTCTACCGTTGTAGATGTGTCCAACCTTCAGGCAAGGTG
crRNA4n8-*Nrl*	ATTTCTACAGAAGTAGATGTGTCCAACCTTCAGGCAAGGTG
crRNA4n9-*Nrl*	ATTTCTACTGTCGTAGATGTGTCCAACCTTCAGGCAAGGTG
crRNA4n10-*Nrl*	ATTTCTACCGGAGTAGATGTGTCCAACCTTCAGGCAAGGTG
crRNA4n11-*Nrl*	ATTTCTACGCTCGTAGATGTGTCCAACCTTCAGGCAAGGTG
crRNA4n12-*Nrl*	ATTTCTACTGTTGTAGATGTGTCCAACCTTCAGGCAAGGTG
crRNA4n13-*Nrl*	ATTTCTACCGGTGTAGATGTGTCCAACCTTCAGGCAAGGTG
crRNA4n14-*Nrl*	ATTTCTACGAGTGTAGATGTGTCCAACCTTCAGGCAAGGTG

crRNA4n15-*Nrl*	ATTTCTACGACAGTAGATGTGTCCAACCTTCAGGCAAGGTG
crRNA4n16-*Nrl*	ATTTCTACCCACGTAGATGTGTCCAACCTTCAGGCAAGGTG
crRNA4n17-*Nrl*	ATTTCTACGACTGTAGATGTGTCCAACCTTCAGGCAAGGTG
crRNA4n18-*Nrl*	ATTTCTACCACAGTAGATGTGTCCAACCTTCAGGCAAGGTG
crRNA4n19-*Nrl*	ATTTCTACCCGCGTAGATGTGTCCAACCTTCAGGCAAGGTG
crRNA4n20-*Nrl*	ATTTCTACGTATGTAGATGTGTCCAACCTTCAGGCAAGGTG
crRNA4n21-*Nrl*	ATTTCTACTCATGTAGATGTGTCCAACCTTCAGGCAAGGTG
crRNA4n22-*Nrl*	ATTTCTACTTCCGTAGATGTGTCCAACCTTCAGGCAAGGTG
crRNA4n23-*Nrl*	ATTTCTACGTCCGTAGATGTGTCCAACCTTCAGGCAAGGTG
crRNA4n24-*Nrl*	ATTTCTACCGATGTAGATGTGTCCAACCTTCAGGCAAGGTG
crRNA4n25-*Nrl*	ATTTCTACGGTAGTAGATGTGTCCAACCTTCAGGCAAGGTG
crRNA4n26-*Nrl*	ATTTCTACCACTGTAGATGTGTCCAACCTTCAGGCAAGGTG
crRNA4n27-*Nrl*	ATTTCTACAAAAGTAGATGTGTCCAACCTTCAGGCAAGGTG
crRNA4n28-*Nrl*	ATTTCTACGCGGGTAGATGTGTCCAACCTTCAGGCAAGGTG
crRNA4n29-*Nrl*	ATTTCTACACCAGTAGATGTGTCCAACCTTCAGGCAAGGTG
crRNA4n30-*Nrl*	ATTTCTACCTGAGTAGATGTGTCCAACCTTCAGGCAAGGTG
crRNA4n31-*Nrl*	ATTTCTACGTGTGTAGATGTGTCCAACCTTCAGGCAAGGTG
crRNA4n32-*Nrl*	ATTTCTACCTTTGTAGATGTGTCCAACCTTCAGGCAAGGTG
crRNA4n33-*Nrl*	ATTTCTACAATTGTAGATGTGTCCAACCTTCAGGCAAGGTG
crRNA4n34-*Nrl*	ATTTCTACCACGGTAGATGTGTCCAACCTTCAGGCAAGGTG
crRNA4n35-*Nrl*	ATTTCTACGCTTGTAGATGTGTCCAACCTTCAGGCAAGGTG
crRNA4n36-*Nrl*	ATTTCTACGGATGTAGATGTGTCCAACCTTCAGGCAAGGTG
crRNA4n37-*Nrl*	ATTTCTACATTGGTAGATGTGTCCAACCTTCAGGCAAGGTG
crRNA4n38-*Nrl*	ATTTCTACAGGCGTAGATGTGTCCAACCTTCAGGCAAGGTG
crRNA4n39-*Nrl*	ATTTCTACGGAAGTAGATGTGTCCAACCTTCAGGCAAGGTG
crRNA4n40-*Nrl*	ATTTCTACTGTAGTAGATGTGTCCAACCTTCAGGCAAGGTG
crRNA4n41-*Nrl*	ATTTCTACCAGCGTAAATGTGTCCAACCTTCAGGCAAGGTG
crRNA4n42-*Nrl*	ATTTCTACCGCAGTAGATGTGTCCAACCTTCAGGCAAGGTG
crRNA4n43-*Nrl*	ATTTCTACTACTGTAGATGTGTCCAACCTTCAGGCAAGGTG
crRNA4n44-*Nrl*	ATTTCTACACCTGTAGATGTGTCCAACCTTCAGGCAAGGTG
crRNA4n45-*Nrl*	ATTTCTACACGTGTAGATGTGTCCAACCTTCAGGCAAGGTG
crRNA4n46-*Nrl*	ATTTCTACCCGTGTAGATGTGTCCAACCTTCAGGCAAGGTG
crRNA4n47-*Nrl*	ATTTCTACTAACGTAGATGTGTCCAACCTTCAGGCAAGGTG
crRNA4n48-*Nrl*	ATTTCTACCTAAGTAGATGTGTCCAACCTTCAGGCAAGGTG
crRNA4n49-*Nrl*	ATTTCTACGGAGGTAGATGTGTCCAACCTTCAGGCAAGGTG
crRNA4n50-*Nrl*	ATTTCTACCGGGGTAGATGTGTCCAACCTTCAGGCAAGGTG
crRNA4n51-*Nrl*	ATTTCTACTCTTGTAGATGTGTCCAACCTTCAGGCAAGGTG
crRNA4n52-*Nrl*	ATTTCTACTGCAGTAGATGTGTCCAACCTTCAGGCAAGGTG
crRNA4n53-*Nrl*	ATTTCTACTTAAGTAGATGTGTCCAACCTTCAGGCAAGGTG
crRNA4n54-*Nrl*	ATTTCTACCATAGTAGATGTGTCCAACCTTCAGGCAAGGTG
crRNA4n55-*Nrl*	ATTTCTACGTGGGTAGATGTGTCCAACCTTCAGGCAAGGTG
crRNA4n56-*Nrl*	ATTTCTACAGGAGTAGATGTGTCCAACCTTCAGGCAAGGTG

crRNA4n57-*Nrl*	ATTTCTACCCTCGTAGATGTGTCCAACCTTCAGGCAAGGTG
crRNA4n58-*Nrl*	ATTTCTACGTCAGTAGATGTGTCCAACCTTCAGGCAAGGTG
crRNA4n59-*Nrl*	ATTTCTACACCGGTAGATGTGTCCAACCTTCAGGCAAGGTG
crRNA4n60-*Nrl*	ATTTCTACAACGGTAGATGTGTCCAACCTTCAGGCAAGGTG
crRNA4n61-*Nrl*	ATTTCTACTTGTGTAGATGTGTCCAACCTTCAGGCAAGGTG
crRNA4n62-*Nrl*	ATTTCTACTAGTGTAGATGTGTCCAACCTTCAGGCAAGGTG
crRNA4n63-*Nrl*	ATTTCTACGGTGGTAGATGTGTCCAACCTTCAGGCAAGGTG
crRNA4n64-*Nrl*	ATTTCTACTCGGGTAGATGTGTCCAACCTTCAGGCAAGGTG
crRNA4n65-*Nrl*	ATTTCTACCTTGGTAGATGTGTCCAACCTTCAGGCAAGGTG
crRNA4n66-*Nrl*	ATTTCTACATTCGTAGATGTGTCCAACCTTCAGGCAAGGTG
crRNA4n67-*Nrl*	ATTTCTACTGCGGTAGATGTGTCCAACCTTCAGGCAAGGTG
crRNA4n68-*Nrl*	ATTTCTACCCCTGTAGATGTGTCCAACCTTCAGGCAAGGTG
crRNA4n69-*Nrl*	ATTTCTACTTTCGTAGATGTGTCCAACCTTCAGGCAAGGTG
crRNA4n70-*Nrl*	ATTTCTACAAATGTAGATGTGTCCAACCTTCAGGCAAGGTG
crRNA4n71-*Nrl*	ATTTCTACTAGAGTAGATGTGTCCAACCTTCAGGCAAGGTG
crRNA4n72-*Nrl*	ATTTCTACACTAGTAGATGTGTCCAACCTTCAGGCAAGGTG
crRNA4n73-*Nrl*	ATTTCTACTTGCGTAGATGTGTCCAACCTTCAGGCAAGGTG
crRNA4n74-*Nrl*	ATTTCTACGTAGGTAGATGTGTCCAACCTTCAGGCAAGGTG
crRNA4n75-*Nrl*	ATTTCTACAGCGGTAGATGTGTCCAACCTTCAGGCAAGGTG
crRNA4n76-*Nrl*	ATTTCTACTTATGTAGATGTGTCCAACCTTCAGGCAAGGTG
crRNA4n77-*Nrl*	ATTTCTACGGGCGTAGATGTGTCCAACCTTCAGGCAAGGTG
crRNA4n78-*Nrl*	ATTTCTACTAATGTAGATGTGTCCAACCTTCAGGCAAGGTG
crRNA4n79-*Nrl*	ATTTCTACTCCAGTAGATGTGTCCAACCTTCAGGCAAGGTG
crRNA4n80-*Nrl*	ATTTCTACTTTTGTAGATGTGTCCAACCTTCAGGCAAGGTG
crRNA4n81-*Nrl*	ATTTCTACCATGGTAGATGTGTCCAACCTTCAGGCAAGGTG
crRNA4n82-*Nrl*	ATTTCTACGCCTGTAGATGTGTCCAACCTTCAGGCAAGGTG
crRNA4n83-*Nrl*	ATTTCTACAATAGTAGATGTGTCCAACCTTCAGGCAAGGTG
crRNA4n84-*Nrl*	ATTTCTACGAGCGTAGATGTGTCCAACCTTCAGGCAAGGTG
crRNA4n85-*Nrl*	ATTTCTACTATCGTAGATGTGTCCAACCTTCAGGCAAGGTG
crRNA4n86-*Nrl*	ATTTCTACTTACGTAGATGTGTCCAACCTTCAGGCAAGGTG
crRNA4n87-*Nrl*	ATTTCTACCTATGTAGATGTGTCCAACCTTCAGGCAAGGTG
crRNA4n88-*Nrl*	ATTTCTACAGAGGTAGATGTGTCCAACCTTCAGGCAAGGTG
crRNA4n89-*Nrl*	ATTTCTACCTCTGTAGATGTGTCCAACCTTCAGGCAAGGTG
crRNA4n90-*Nrl*	ATTTCTACGTTAGTAGATGTGTCCAACCTTCAGGCAAGGTG
crRNA4n91-*Nrl*	ATTTCTACTGTGGTAGATGTGTCCAACCTTCAGGCAAGGTG
crRNA4n92-*Nrl*	ATTTCTACCCTAGTAGATGTGTCCAACCTTCAGGCAAGGTG
crRNA4n93-*Nrl*	ATTTCTACGCCGGTAGATGTGTCCAACCTTCAGGCAAGGTG
crRNA4n94-*Nrl*	ATTTCTACACAAGTAGATGTGTCCAACCTTCAGGCAAGGTG
crRNA4n95-*Nrl*	ATTTCTACGAAGGTAGATGTGTCCAACCTTCAGGCAAGGTG
crRNA4n96-*Nrl*	ATTTCTACTATGGTAGATGTGTCCAACCTTCAGGCAAGGTG
crRNA4n97-*Nrl*	ATTTCTACAATGGTAGATGTGTCCAACCTTCAGGCAAGGTG
crRNA4n98-*Nrl*	ATTTCTACGACCGTAGATGTGTCCAACCTTCAGGCAAGGTG

crRNA4n99-*Nrl*	ATTTCTACATTAGTAGATGTGTCCAACCTTCAGGCAAGGTG
crRNA4n100-*Nrl*	ATTTCTACAATCGTAGATGTGTCCAACCTTCAGGCAAGGTG
crRNA4n101-*Nrl*	ATTTCTACTTTGGTAGATGTGTCCAACCTTCAGGCAAGGTG
crRNA4n102-*Nrl*	ATTTCTACTACAGTAGATGTGTCCAACCTTCAGGCAAGGTG
crRNA4n103-*Nrl*	ATTTCTACGCAGGTAGATGTGTCCAACCTTCAGGCAAGGTG
crRNA4n104-*Nrl*	ATTTCTACGTTTGTAGATGTGTCCAACCTTCAGGCAAGGTG
crRNA4n105-*Nrl*	ATTTCTACTGACGTAGATGTGTCCAACCTTCAGGCAAGGTG
crRNA4n106-*Nrl*	ATTTCTACGATGGTAGATGTGTCCAACCTTCAGGCAAGGTG
crRNA4n107-*Nrl*	ATTTCTACTGCTGTAGATGTGTCCAACCTTCAGGCAAGGTG
crRNA4n108-*Nrl*	ATTTCTACCCCGGTAGATGTGTCCAACCTTCAGGCAAGGTG
crRNA4n109-*Nrl*	ATTTCTACGTAAGTAGATGTGTCCAACCTTCAGGCAAGGTG
crRNA4n110-*Nrl*	ATTTCTACCCCCGTAGATGTGTCCAACCTTCAGGCAAGGTG
crRNA4n111-*Nrl*	ATTTCTACCGCTGTAGATGTGTCCAACCTTCAGGCAAGGTG
crRNA4n112-*Nrl*	ATTTCTACCCGAGTAGATGTGTCCAACCTTCAGGCAAGGTG
crRNA4n113-*Nrl*	ATTTCTACCTGTGTAGATGTGTCCAACCTTCAGGCAAGGTG
crRNA4n114-*Nrl*	ATTTCTACGGGTGTAGATGTGTCCAACCTTCAGGCAAGGTG
crRNA4n115-*Nrl*	ATTTCTACGATCGTAGATGTGTCCAACCTTCAGGCAAGGTG
crRNA4n116-*Nrl*	ATTTCTACCTTAGTAGATGTGTCCAACCTTCAGGCAAGGTG
crRNA4n117-*Nrl*	ATTTCTACACATGTAGATGTGTCCAACCTTCAGGCAAGGTG
crRNA4n118-*Nrl*	ATTTCTACAAGAGTAGATGTGTCCAACCTTCAGGCAAGGTG
crRNA4n119-*Nrl*	ATTTCTACTAAAGTAGATGTGTCCAACCTTCAGGCAAGGTG
crRNA4n120-*Nrl*	ATTTCTACCCAAGTAGATGTGTCCAACCTTCAGGCAAGGTG
crRNA4n121-*Nrl*	ATTTCTACAGCTGTAGATGTGTCCAACCTTCAGGCAAGGTG
crRNA4n122-*Nrl*	ATTTCTACGCGAGTAGATGTGTCCAACCTTCAGGCAAGGTG
crRNA4n123-*Nrl*	ATTTCTACTGGGGTAGATGTGTCCAACCTTCAGGCAAGGTG
crRNA4n124-*Nrl*	ATTTCTACCCTTGTAGATGTGTCCAACCTTCAGGCAAGGTG
crRNA4n125-*Nrl*	ATTTCTACACGGGTAGATGTGTCCAACCTTCAGGCAAGGTG
crRNA4n126-*Nrl*	ATTTCTACTCACGTAGATGTGTCCAACCTTCAGGCAAGGTG
crRNA4n127-*Nrl*	ATTTCTACACCCGTAGATGTGTCCAACCTTCAGGCAAGGTG
crRNA4n128-*Nrl*	ATTTCTACGCAAGTAGATGTGTCCAACCTTCAGGCAAGGTG
crRNA4n129-*Nrl*	ATTTCTACCAGTGTAGATGTGTCCAACCTTCAGGCAAGGTG
crRNA4n130-*Nrl*	ATTTCTACTTCTGTAGATGTGTCCAACCTTCAGGCAAGGTG
crRNA4n131-*Nrl*	ATTTCTACGTTCGTAGATGTGTCCAACCTTCAGGCAAGGTG
crRNA4n132-*Nrl*	ATTTCTACAGACGTAGATGTGTCCAACCTTCAGGCAAGGTG
crRNA4n133-*Nrl*	ATTTCTACGAACGTAGATGTGTCCAACCTTCAGGCAAGGTG
crRNA4n134-*Nrl*	ATTTCTACATCCGTAGATGTGTCCAACCTTCAGGCAAGGTG
crRNA4n135-*Nrl*	ATTTCTACGCTGGTAGATGTGTCCAACCTTCAGGCAAGGTG
crRNA4n136-*Nrl*	ATTTCTACATGAGTAGATGTGTCCAACCTTCAGGCAAGGTG
crRNA4n137-*Nrl*	ATTTCTACTTCGGTAGATGTGTCCAACCTTCAGGCAAGGTG
crRNA4n138-*Nrl*	ATTTCTACGCGCGTAGATGTGTCCAACCTTCAGGCAAGGTG
crRNA4n139-*Nrl*	ATTTCTACCGTCGTAGATGTGTCCAACCTTCAGGCAAGGTG
crRNA4n140-*Nrl*	ATTTCTACCACCGTAGATGTGTCCAACCTTCAGGCAAGGTG

crRNA4n141-*Nrl*	ATTTCTACACTGGTAGATGTGTCCAACCTTCAGGCAAGGTG
crRNA4n142-*Nrl*	ATTTCTACTCTGGTAGATGTGTCCAACCTTCAGGCAAGGTG
crRNA4n143-*Nrl*	ATTTCTACTCCCGTAGATGTGTCCAACCTTCAGGCAAGGTG
crRNA4n144-*Nrl*	ATTTCTACAAGTGTAGATGTGTCCAACCTTCAGGCAAGGTG
crRNA4n145-*Nrl*	ATTTCTACTATAGTAGATGTGTCCAACCTTCAGGCAAGGTG
crRNA4n146-*Nrl*	ATTTCTACAGCAGTAGATGTGTCCAACCTTCAGGCAAGGTG
crRNA4n147-*Nrl*	ATTTCTACAGGTGTAGATGTGTCCAACCTTCAGGCAAGGTG
crRNA4n148-*Nrl*	ATTTCTACTAAGGTAGATGTGTCCAACCTTCAGGCAAGGTG
crRNA4n149-*Nrl*	ATTTCTACTCCTGTAGATGTGTCCAACCTTCAGGCAAGGTG
crRNA4n150-*Nrl*	ATTTCTACAACAGTAGATGTGTCCAACCTTCAGGCAAGGTG
crRNA4n151-*Nrl*	ATTTCTACAGCCGTAGATGTGTCCAACCTTCAGGCAAGGTG
crRNA4n152-*Nrl*	ATTTCTACCAATGTAGATGTGTCCAACCTTCAGGCAAGGTG
crRNA4n153-*Nrl*	ATTTCTACGTCTGTAGATGTGTCCAACCTTCAGGCAAGGTG
crRNA4n154-*Nrl*	ATTTCTACAAAGGTAGATGTGTCCAACCTTCAGGCAAGGTG
crRNA4n155-*Nrl*	ATTTCTACGCTAGTAGATGTGTCCAACCTTCAGGCAAGGTG
crRNA4n156-*Nrl*	ATTTCTACTTGAGTAGATGTGTCCAACCTTCAGGCAAGGTG
crRNA4n157-*Nrl*	ATTTCTACGCGTGTAGATGTGTCCAACCTTCAGGCAAGGTG
crRNA4n158-*Nrl*	ATTTCTACACACGTAGATGTGTCCAACCTTCAGGCAAGGTG
crRNA4n159-*Nrl*	ATTTCTACTCGTGTAGATGTGTCCAACCTTCAGGCAAGGTG
crRNA4n160-*Nrl*	ATTTCTACACTTGTAGATGTGTCCAACCTTCAGGCAAGGTG
crRNA4n161-*Nrl*	ATTTCTACCATTGTAGATGTGTCCAACCTTCAGGCAAGGTG
crRNA4n162-*Nrl*	ATTTCTACACTCGTAGATGTGTCCAACCTTCAGGCAAGGTG
crRNA4n163-*Nrl*	ATTTCTACTTCAGTAGATGTGTCCAACCTTCAGGCAAGGTG
crRNA4n164-*Nrl*	ATTTCTACCTAGGTAGATGTGTCCAACCTTCAGGCAAGGTG
crRNA4n165-*Nrl*	ATTTCTACACGCGTAGATGTGTCCAACCTTCAGGCAAGGTG
crRNA4n166-*Nrl*	ATTTCTACCCAGGTAGATGTGTCCAACCTTCAGGCAAGGTG
crRNA4n167-*Nrl*	ATTTCTACGAGAGTAGATGTGTCCAACCTTCAGGCAAGGTG
crRNA4n168-*Nrl*	ATTTCTACCATCGTAGATGTGTCCAACCTTCAGGCAAGGTG
crRNA4n169-*Nrl*	ATTTCTACGTGCGTAGATGTGTCCAACCTTCAGGCAAGGTG
crRNA4n170-*Nrl*	ATTTCTACCCATGTAGATGTGTCCAACCTTCAGGCAAGGTG
crRNA4n171-*Nrl*	ATTTCTACATATGTAGATGTGTCCAACCTTCAGGCAAGGTG
crRNA4n172-*Nrl*	ATTTCTACTGGTGTAGATGTGTCCAACCTTCAGGCAAGGTG
crRNA4n173-*Nrl*	ATTTCTACTCAAGTAATGTGTCCAACCTTCAGGCAAGGTG
crRNA4n174-*Nrl*	ATTTCTACGGTTGTAGATGTGTCCAACCTTCAGGCAAGGTG
crRNA4n175-*Nrl*	ATTTCTACTCTAGTAGATGTGTCCAACCTTCAGGCAAGGTG
crRNA4n176-*Nrl*	ATTTCTACGGCCGTAGATGTGTCCAACCTTCAGGCAAGGTG
crRNA4n177-*Nrl*	ATTTCTACTAGGGTAGATGTGTCCAACCTTCAGGCAAGGTG
crRNA4n178-*Nrl*	ATTTCTACTGAGGTAGATGTGTCCAACCTTCAGGCAAGGTG
crRNA4n179-*Nrl*	ATTTCTACATCAGTAGATGTGTCCAACCTTCAGGCAAGGTG
crRNA4n180-*Nrl*	ATTTCTACGCACGTAGATGTGTCCAACCTTCAGGCAAGGTG
crRNA4n181-*Nrl*	ATTTCTACCGTAGTAGATGTGTCCAACCTTCAGGCAAGGTG
crRNA4n182-*Nrl*	ATTTCTACATAGGTAGATGTGTCCAACCTTCAGGCAAGGTG

crRNA4n183-*Nrl*	ATTTCTACCCTGGTAGATGTGTCCAACCTTCAGGCAAGGTG
crRNA4n184-*Nrl*	ATTTCTACTCAGGTAGATGTGTCCAACCTTCAGGCAAGGTG
crRNA4n185-*Nrl*	ATTTCTACCTCGGTAGATGTGTCCAACCTTCAGGCAAGGTG
crRNA4n186-*Nrl*	ATTTCTACGCATGTAGATGTGTCCAACCTTCAGGCAAGGTG
crRNA4n187-*Nrl*	ATTTCTACAGTCGTAGATGTGTCCAACCTTCAGGCAAGGTG
crRNA4n188-*Nrl*	ATTTCTACGACGGTAGATGTGTCCAACCTTCAGGCAAGGTG
crRNA4n189-*Nrl*	ATTTCTACCGTGGTAGATGTGTCCAACCTTCAGGCAAGGTG
crRNA4n190-*Nrl*	ATTTCTACCTTCGTAGATGTGTCCAACCTTCAGGCAAGGTG
crRNA4n191-*Nrl*	ATTTCTACAACTGTAGATGTGTCCAACCTTCAGGCAAGGTG
crRNA4n192-*Nrl*	ATTTCTACGTTGGTAGATGTGTCCAACCTTCAGGCAAGGTG
crRNA4n193-*Nrl*	ATTTCTACAGTGGTAGATGTGTCCAACCTTCAGGCAAGGTG
crRNA4n194-*Nrl*	ATTTCTACAAGCGTAGATGTGTCCAACCTTCAGGCAAGGTG
crRNA4n195-*Nrl*	ATTTCTACATTTGTAGATGTGTCCAACCTTCAGGCAAGGTG
crRNA4n196-*Nrl*	ATTTCTACCCCAGTAGATGTGTCCAACCTTCAGGCAAGGTG
crRNA4n197-*Nrl*	ATTTCTACGGGAGTAGATGTGTCCAACCTTCAGGCAAGGTG
crRNA4n198-*Nrl*	ATTTCTACCGAAGTAGATGTGTCCAACCTTCAGGCAAGGTG
crRNA4n199-*Nrl*	ATTTCTACGCCCGTAGATGTGTCCAACCTTCAGGCAAGGTG
crRNA4n200-*Nrl*	ATTTCTACGAATGTAGATGTGTCCAACCTTCAGGCAAGGTG
crRNA4n201-*Nrl*	ATTTCTACAAACGTAGATGTGTCCAACCTTCAGGCAAGGTG
crRNA4n202-*Nrl*	ATTTCTACCTACGTAGATGTGTCCAACCTTCAGGCAAGGTG
crRNA4n203-*Nrl*	ATTTCTACCAACGTAGATGTGTCCAACCTTCAGGCAAGGTG
crRNA4n204-*Nrl*	ATTTCTACTGGAGTAGATGTGTCCAACCTTCAGGCAAGGTG
crRNA4n205-*Nrl*	ATTTCTACATGCGTAGATGTGTCCAACCTTCAGGCAAGGTG
crRNA4n206-*Nrl*	ATTTCTACAACCGTAGATGTGTCCAACCTTCAGGCAAGGTG
crRNA4n207-*Nrl*	ATTTCTACATGTGTAGATGTGTCCAACCTTCAGGCAAGGTG
crRNA4n208-*Nrl*	ATTTCTACATGGGTAGATGTGTCCAACCTTCAGGCAAGGTG
crRNA4n209-*Nrl*	ATTTCTACGTGAGTAGATGTGTCCAACCTTCAGGCAAGGTG
crRNA4n210-*Nrl*	ATTTCTACATAAGTAGATGTGTCCAACCTTCAGGCAAGGTG
crRNA4n211-*Nrl*	ATTTCTACGGTCGTAGATGTGTCCAACCTTCAGGCAAGGTG
crRNA4n212-*Nrl*	ATTTCTACGGGGGTAGATGTGTCCAACCTTCAGGCAAGGTG
crRNA4n213-*Nrl*	ATTTCTACAGTAGTAGATGTGTCCAACCTTCAGGCAAGGTG
crRNA4n214-*Nrl*	ATTTCTACATCGGTAGATGTGTCCAACCTTCAGGCAAGGTG
crRNA4n215-*Nrl*	ATTTCTACTACGGTAGATGTGTCCAACCTTCAGGCAAGGTG
crRNA4n216-*Nrl*	ATTTCTACTGCCGTAGATGTGTCCAACCTTCAGGCAAGGTG
crRNA4n217-*Nrl*	ATTTCTACCCGGGTAGATGTGTCCAACCTTCAGGCAAGGTG
crRNA4n218-*Nrl*	ATTTCTACTACCGTAGATGTGTCCAACCTTCAGGCAAGGTG
crRNA4n219-*Nrl*	ATTTCTACTTTAGTAGATGTGTCCAACCTTCAGGCAAGGTG
crRNA4n220-*Nrl*	ATTTCTACCTGGGTAGATGTGTCCAACCTTCAGGCAAGGTG
crRNA4n221-*Nrl*	ATTTCTACCAAGGTAGATGTGTCCAACCTTCAGGCAAGGTG
crRNA4n222-*Nrl*	ATTTCTACCAGAGTAGATGTGTCCAACCTTCAGGCAAGGTG
crRNA4n223-*Nrl*	ATTTCTACTCGAGTAGATGTGTCCAACCTTCAGGCAAGGTG
crRNA4n224-*Nrl*	ATTTCTACATACGTAGATGTGTCCAACCTTCAGGCAAGGTG

crRNA4n225-*Nrl*	ATTTCTACCGACGTAGATGTGTCCAACCTTCAGGCAAGGTG
crRNA4n226-*Nrl*	ATTTCTACGGACGTAGATGTGTCCAACCTTCAGGCAAGGTG
crRNA4n227-*Nrl*	ATTTCTACCAAAGTAGATGTGTCCAACCTTCAGGCAAGGTG
crRNA4n228-*Nrl*	ATTTCTACGAAAGTAGATGTGTCCAACCTTCAGGCAAGGTG
crRNA4n229-*Nrl*	ATTTCTACACAGGTAGATGTGTCCAACCTTCAGGCAAGGTG
crRNA4n230-*Nrl*	ATTTCTACCGAGGTAGATGTGTCCAACCTTCAGGCAAGGTG
crRNA4n231-*Nrl*	ATTTCTACAGATGTAGATGTGTCCAACCTTCAGGCAAGGTG
crRNA4n232-*Nrl*	ATTTCTACTCTCGTAGATGTGTCCAACCTTCAGGCAAGGTG
crRNA4n233-*Nrl*	ATTTCTACGATTGTAGATGTGTCCAACCTTCAGGCAAGGTG
crRNA4n234-*Nrl*	ATTTCTACAGTTGTAGATGTGTCCAACCTTCAGGCAAGGTG
crRNA4n235-*Nrl*	ATTTCTACCTCCGTAGATGTGTCCAACCTTCAGGCAAGGTG
crRNA4n236-*Nrl*	ATTTCTACCGCCGTAGATGTGTCCAACCTTCAGGCAAGGTG
crRNA4n237-*Nrl*	ATTTCTACCTCAGTAGATGTGTCCAACCTTCAGGCAAGGTG
crRNA4n238-*Nrl*	ATTTCTACGCCAGTAGATGTGTCCAACCTTCAGGCAAGGTG
crRNA4n239-*Nrl*	ATTTCTACGGCAGTAGATGTGTCCAACCTTCAGGCAAGGTG
crRNA4n240-*Nrl*	ATTTCTACGTCGGTAGATGTGTCCAACCTTCAGGCAAGGTG
crRNA4n241-*Nrl*	ATTTCTACTCCGGTAGATGTGTCCAACCTTCAGGCAAGGTG
crRNA4n242-*Nrl*	ATTTCTACCGCGGTAGATGTGTCCAACCTTCAGGCAAGGTG
crRNA4n243-*Nrl*	ATTTCTACGGCGGTAGATGTGTCCAACCTTCAGGCAAGGTG
crRNA4n244-*Nrl*	ATTTCTACATCTGTAGATGTGTCCAACCTTCAGGCAAGGTG
crRNA4n245-*Nrl*	ATTTCTACGGCTGTAGATGTGTCCAACCTTCAGGCAAGGTG
crRNA4n246-*Nrl*	ATTTCTACTTAGGTAGATGTGTCCAACCTTCAGGCAAGGTG
crRNA4n247-*Nrl*	ATTTCTACCTGCGTAGATGTGTCCAACCTTCAGGCAAGGTG
crRNA4n248-*Nrl*	ATTTCTACTCGCGTAGATGTGTCCAACCTTCAGGCAAGGTG
crRNA4n249-*Nrl*	ATTTCTACCGGCGTAGATGTGTCCAACCTTCAGGCAAGGTG
crRNA4n250-*Nrl*	ATTTCTACTGGCGTAGATGTGTCCAACCTTCAGGCAAGGTG
crRNA4n251-*Nrl*	ATTTCTACAAGGGTAGATGTGTCCAACCTTCAGGCAAGGTG
crRNA4n252-*Nrl*	ATTTCTACCAGGGTAGATGTGTCCAACCTTCAGGCAAGGTG
crRNA4n253-*Nrl*	ATTTCTACGAGGGTAGATGTGTCCAACCTTCAGGCAAGGTG
crRNA4n254-*Nrl*	ATTTCTACTTGGGTAGATGTGTCCAACCTTCAGGCAAGGTG
crRNA4n255-*Nrl*	ATTTCTACAGGGGTAGATGTGTCCAACCTTCAGGCAAGGTG
crRNA4n178-*MeCP2*	ATTTCTACTGAGGTAGATCCTGCCTCTGCTGGCTCTGCAGA
crRNA4n213-*MeCP2*	ATTTCTACAGTAGTAGATCCTGCCTCTGCTGGCTCTGCAGA
crRNA4n234-*MeCP2*	ATTTCTACAGTTGTAGATCCTGCCTCTGCTGGCTCTGCAGA
crRNA4n100-*MeCP2*	ATTTCTACAATCGTAGATCCTGCCTCTGCTGGCTCTGCAGA
crRNA4n128-*MeCP2*	ATTTCTACGCAAGTAGATCCTGCCTCTGCTGGCTCTGCAGA
crRNA4n90-*MeCP2*	ATTTCTACGTTAGTAGATCCTGCCTCTGCTGGCTCTGCAGA
crRNA4n86-*MeCP2*	ATTTCTACTTACGTAGATCCTGCCTCTGCTGGCTCTGCAGA
crRNA4n103-*MeCP2*	ATTTCTACGCAGGTAGATCCTGCCTCTGCTGGCTCTGCAGA
crRNA4n130-*MeCP2*	ATTTCTACTTCTGTAGATCCTGCCTCTGCTGGCTCTGCAGA
crRNA4n250-*MeCP2*	ATTTCTACTGGCGTAGATCCTGCCTCTGCTGGCTCTGCAGA
crRNA4n216-*MeCP2*	ATTTCTACTGCCGTAGATCCTGCCTCTGCTGGCTCTGCAGA

crRNA4n10-*MeCP2*	ATTTCTACCGGAGTAGATCCTGCCTCTGCTGGCTCTGCAGA
crRNA4n110-*MeCP2*	ATTTCTACCCCCGTAGATCCTGCCTCTGCTGGCTCTGCAGA
crRNA4n141-*MeCP2*	ATTTCTACACTGGTAGATCCTGCCTCTGCTGGCTCTGCAGA
crRNA4n148-*MeCP2*	ATTTCTACTAAGGTAGATCCTGCCTCTGCTGGCTCTGCAGA
crRNA4n68-*MeCP2*	ATTTCTACCCCTGTAGATCCTGCCTCTGCTGGCTCTGCAGA
crRNA4n124-*MeCP2*	ATTTCTACCCTTGTAGATCCTGCCTCTGCTGGCTCTGCAGA
crRNA4n120-*MeCP2*	ATTTCTACCCAAGTAGATCCTGCCTCTGCTGGCTCTGCAGA
crRNA4n132-*MeCP2*	ATTTCTACAGACGTAGATCCTGCCTCTGCTGGCTCTGCAGA
crRNA4n156-*MeCP2*	ATTTCTACTTGAGTAGATCCTGCCTCTGCTGGCTCTGCAGA
crRNA4n93-*MeCP2*	ATTTCTACGCCGGTAGATCCTGCCTCTGCTGGCTCTGCAGA
crRNA4n232-*MeCP2*	ATTTCTACTCTCGTAGATCCTGCCTCTGCTGGCTCTGCAGA
crRNA4n96-*MeCP2*	ATTTCTACTATGGTAGATCCTGCCTCTGCTGGCTCTGCAGA
crRNA4n89-*MeCP2*	ATTTCTACCTCTGTAGATCCTGCCTCTGCTGGCTCTGCAGA
crRNA4n80-*MeCP2*	ATTTCTACTTTTGTAGATCCTGCCTCTGCTGGCTCTGCAGA
crRNA3n1-*MeCP2*	ATTTCTACACGGTAGATCCTGCCTCTGCTGGCTCTGCAGA
crRNA3n2-*MeCP2*	ATTTCTACAGCGTAGATCCTGCCTCTGCTGGCTCTGCAGA
crRNA3n3-*MeCP2*	ATTTCTACTGGGTAGATCCTGCCTCTGCTGGCTCTGCAGA
crRNA3n4-*MeCP2*	ATTTCTACCGTGTAGATCCTGCCTCTGCTGGCTCTGCAGA
crRNA3n5-*MeCP2*	ATTTCTACCATGTAGATCCTGCCTCTGCTGGCTCTGCAGA
crRNA3n5-*MeCP2*	ATTTCTACCTCGTAGATCCTGCCTCTGCTGGCTCTGCAGA
crRNA3n7-*MeCP2*	ATTTCTACCCTGTAGATCCTGCCTCTGCTGGCTCTGCAGA
crRNA3n8-*MeCP2*	ATTTCTACGTCGTAGATCCTGCCTCTGCTGGCTCTGCAGA
crRNA3n9-*MeCP2*	ATTTCTACGGCGTAGATCCTGCCTCTGCTGGCTCTGCAGA
crRNA3n10-*MeCP2*	ATTTCTACGTTGTAGATCCTGCCTCTGCTGGCTCTGCAGA
crRNA5n1-*MeCP2*	ATTTCTACAACCCGTAGATCCTGCCTCTGCTGGCTCTGCAGA
crRNA5n2-*MeCP2*	ATTTCTACGCGGGGTAGATCCTGCCTCTGCTGGCTCTGCAGA
crRNA5n3-*MeCP2*	ATTTCTACCTGACGTAGATCCTGCCTCTGCTGGCTCTGCAGA
crRNA5n4-*MeCP2*	ATTTCTACAACCAGTAGATCCTGCCTCTGCTGGCTCTGCAGA
crRNA5n5-*MeCP2*	ATTTCTACTATTTGTAGATCCTGCCTCTGCTGGCTCTGCAGA
crRNA5n6-*MeCP2*	ATTTCTACCACCCGTAGATCCTGCCTCTGCTGGCTCTGCAGA
crRNA5n7-*MeCP2*	ATTTCTACCGCATGTAGATCCTGCCTCTGCTGGCTCTGCAGA
crRNA5n8-*MeCP2*	ATTTCTACGCTAGGTAGATCCTGCCTCTGCTGGCTCTGCAGA
crRNA5n9-*MeCP2*	ATTTCTACGGTGTGTAGATCCTGCCTCTGCTGGCTCTGCAGA
crRNA5n10-*MeCP2*	ATTTCTACTCATTGTAGATCCTGCCTCTGCTGGCTCTGCAGA
crRNA5n11-*MeCP2*	ATTTCTACCCCCCGTAGATCCTGCCTCTGCTGGCTCTGCAGA
crRNA5n12-*MeCP2*	ATTTCTACCGCCGGTAGATCCTGCCTCTGCTGGCTCTGCAGA
crRNA5n13-*MeCP2*	ATTTCTACTGGTGGTAGATCCTGCCTCTGCTGGCTCTGCAGA
crRNA5n14-*MeCP2*	ATTTCTACTGCGCGTAGATCCTGCCTCTGCTGGCTCTGCAGA
crRNA5n15-*MeCP2*	ATTTCTACTCGCAGTAGATCCTGCCTCTGCTGGCTCTGCAGA
crRNA5n16-*MeCP2*	ATTTCTACGAGATGTAGATCCTGCCTCTGCTGGCTCTGCAGA
crRNA5n17-*MeCP2*	ATTTCTACACGTGGTAGATCCTGCCTCTGCTGGCTCTGCAGA
crRNA5n18-*MeCP2*	ATTTCTACAATTAGTAGATCCTGCCTCTGCTGGCTCTGCAGA

crRNA5n19-*MeCP2*	ATTTCTACACCGGGTAGATCCTGCCTCTGCTGGCTCTGCAGA
crRNA5n20-*MeCP2*	ATTTCTACTGCAAGTAGATCCTGCCTCTGCTGGCTCTGCAGA
crRNA5n21-*MeCP2*	ATTTCTACTAATTGTAGATCCTGCCTCTGCTGGCTCTGCAGA
crRNA5n22-*MeCP2*	ATTTCTACTAGTTGTAGATCCTGCCTCTGCTGGCTCTGCAGA
crRNA5n23-*MeCP2*	ATTTCTACTTATTGTAGATCCTGCCTCTGCTGGCTCTGCAGA
crRNA5n24-*MeCP2*	ATTTCTACTTTTTGTAGATCCTGCCTCTGCTGGCTCTGCAGA
crRNA5n25-*MeCP2*	ATTTCTACTGTTTGTAGATCCTGCCTCTGCTGGCTCTGCAGA

Table S6. Primer sequences used for PCR amplification.

Primer Name	Primer sequences (5'-3')	Length	Species
p11-F<br>p11-R	AGTGAGCGAGGAAGCGGAAGA<br>CAGGAGAGCGTTCACCGACAA	436 bp	p11-LacY-wtx
AAVS1-F<br>AAVS1-R	TTATATTGTTCCTCCGTGCGTCAGTT<br>CCCGAAGAGTGAGTTTGCCAAG	585 bp	*Homo sapiens*
CD34-F<br>CD34-R	TTGAAATGAGTTTGGTCAGGGATGG<br>AACTGTGTATTTCCGTGCTGATTCC	550 bp	
RNF2-F<br>RNF2-R	GGAGCTGTAGGCGATTATAGTTGAA<br>TTCTCAAACCCTGGAAAGCACTTT	403 bp	
DNMT1-F<br>DNMT1-R	CTCCTGCTCGGTGAATTTGG<br>TAGTTGATAAGCGAACCTCACAC	452 bp	
CCR5-F<br>CCR5-R	GCAGCTCTCATTTTCCATACAGT<br>GATCGGGTGTAAACTGAGCTTG	493 bp	
EXM1-F<br>EMX1-R	AGCCTCAGTCTTCCCATCAG<br>CCATTGCTTGTCCCTCTGTC	451 bp	
MeCP2-F<br>MeCP2-R	GCACTCAAGCTCACCTATACTCACT<br>TACCCAGACCTAATCCACCACCAA	593 bp	*Mus musculus*
Nrl-F<br>Nrl-R	GCCTCTCAGTGTTCTACACCTTCC<br>CCATAGAGACAGGACCCTGGTTCT	386 bp	
Rosa26-F<br>Rosa26-R	TCCAAATTCTTCACAGCCAAAGTCA<br>GGAGCCTGCCAAGTAACTACTCTT	405 bp	

Table S7. Frequency of Cas12a- and SpCas9-mediated targeted indel mutations at on-target sites in human 293FT cells. DNA sequences and indel frequency of ArCas12a, BsCas12a, HkCas12a, PrCas12a and SpCas9 target sites are shown. For Cas12a, crRNA scaffold 1 and 4n96 were used separately. Cas12a and SpCas9 PAMs are shown in red and blue, respectively. Indel frequency is assessed by T7EI assay with means and standard errors of the mean (s.e.m.) shown, n = 2.

Target sites	ArCas12a		BsCas12a		HkCas12a		PrCas12a		SpCas9
Guide RNA	4n96	1	4n96	1	4n96	1	4n96	1	sgRNA
CCTTTTATTTCCCTTCAGCT AAAATAAAGGAGGAGGAAGC	9.5 ± 0.8	6.2 ± 1.0	10.5 ± 0.9	6.7 ± 0.5	10.5 ± 0.6	4.7 ± 0.3	15.2 ± 1.1	12.2 ± 1.2	25.5 ± 1.5
GGAATTCTTTGGCCTGAATA ATTGCAGTAGCTCTAACAGG	8.3 ± 0.5	5.5 ± 0.5	16.5 ± 1.2	9.8 ± 1.1	12.1 ± 0.8	6.6 ± 0.8	10.2 ± 1.2	6.5 ± 0.4	20.2 ± 1.1
CGATGTCACCTCCAATGACT AGGGTGGGCAACCACAAACC	9.0 ± 1.1	5.4 ± 0.7	15.8 ± 1.3	10.0 ± 0.9	16.7 ± 1.2	9.8 ± 1.1	17.8 ± 0.7	11.5 ± 0.9	25.3 ± 1.4
TTCCTTTTATTTCCCTTCAG CTAAAATAAAGGAGGAGGAA	12.1 ± 1.2	8.5 ± 0.8	14.2 ± 1.1	9.9 ± 0.6	13.2 ± 1.1	8.8 ± 0.9	16.5 ± 1.2	10.6 ± 1.0	20.0 ± 1.1

Table S8. Off-target analysis by targeted deep sequencing in the human 293FT cells. Target and off-target sequences of the human *DNMT1* site 1, and indel frequency of BsCas12a, PrCas12a and SpCas9 are shown. For Cas12a, crRNA scaffold 1 and 4n96 were used separately. Cas12a and SpCas9 PAMs are underlined. Indel frequency is assessed by targeted deep sequencing.

Cas12a		PrCas12a		BsCas12a		Cas9		SpCas9
ID	On/Off-target sequence	crRNA4n96	crRNA1	crRNA4n96	crRNA1	ID	On/Off-target sequence	sgRNA
ON	TTCCCTTCAGCTAAAATAAAGGAGGA	12.62%	7.65%	9.90%	3.89%	ON	CCCTTCAGCTAAAATAAAGGAGG	21.76%
OT1	TTGCtTTCAGCTAAAcTAAAaGAGGA	0	0	0	0.021%	OT1	CCCTgaAGCTAAAATAAAtGGGG	0.046%
OT2	TTCCCTTCAGgTAAAATtAgGGAGGA	0	0.0086%	0	0	OT2	CCCTTCAGCTAAAgTcAAaGGGG	0.032%
OT3	TTAtCTTCAaCTAAAATAAAGaAaGA	0	0.0053%	0.0033%	0	OT3	CCCTgCAGCTAgAATAAAGcAGG	0.049%
OT4	TTCCCTcCAtCTAAAATgAAGGAaGA	0	0.038%	0	0.0029%	OT4	gCCcTCAGCTAAAATgAAGGGGG	0
OT5	TTGCtTTCAaCcAAAtTAAAGGAGGA	0	0.013%	0.0075%	0.0071%	OT5	CCCTTCAGgTAgAAcAAAGGAGG	0.0056%
OT6	TTGCCTcCAtCTAAAATcAAGGAGaA	0.020%	0	0.0074%	0	OT6	CCCTTCAGCTAAAATAcAGtGGG	0.032%
OT7	TTGCCTTCAGgTAAAATAAAGagaGA	0.0086%	0.0066%	0	0	OT7	gCCTTCAGtTAAcATAAAGGGGG	0.099%

OT8	TTTCCTTCAGCaAcAgTAAAGGAGaA	0.016%	0.026%	0.021%	0.019%	OT8	CCCTTCAGCTAAAAccgAGGTGG	0
OT9	TTGCCaTCAGCTAAAATcAAGGtaGA	0.0064%	0.0052%	0.0074%	0.015%	OT9	CCCTTCAGCTAAAAgAtAGGAGG	0.0028%
OT10	TTCagTTCAGCTAcAATAAAGaAGGA	0	0	0	0	OT10	CCCTTtAGCTgAAATAAAGtTGG	0.012%
OT11	TTCCCTTCAGCTAAAATAAtGaAttA	0	0	0	0	OT11	CCCTTCAGCTAcAATAgAaGGGG	0
OT12	TTTCCTTCAGtaAAgAaAAAGGAGGA	0	0	0	0	OT12	CaCTTaAGCTAAAATcAAGGTGG	0
OT13	TTCaCTTaAGCTAAAATcAAGGtGGA	0.027%	0.011%	0.011%	0.034%	OT13	CCCTTCActTAAAAaAAAGGAGG	0.014%
OT14	TTCCCTTCAcCTggAATAAAGcAGGA	0	0.010%	0.0049%	0	OT14	gCtTTCAGCTAAAATAAAtGGGG	0.013%
OT15	TTGCCTTCAGtaAAAtTAAAGGAaGA	0.013%	0	0.0045%	0	OT15	CCCTTCAGgTAAAATtAgGGAGG	0.0058%
OT16	TTTCCTTCAtCaAAAATAAAGGAaGg	0	0	0	0	-	-	-
OT17	TTACCcTCtGCTAAAAaAAAtGAGGA	0.0095%	0.0086%	0.011%	0.0086%	-	-	-
OT18	TTGCCTTCtGCTAAAATgAAGaAGGg	0	0.022%	0	0	-	-	-
OT19	TTAtaTgCAGCTAAAATAAAtGAGGA	0	0	0	0	-	-	-
OT20	TTGCtTTCAaCaAAAtTAAAGGAGGA	0	0	0.0064%	0	-	-	-

Table S9. Off-target analysis by whole genome sequencing (WGS) in human 293FT cells. Potential off-target sites with one to five mismatches to the targeting guide RNA were analyzed by WGS. Indel frequencies of human *DNMT1* and *CCR5* target sites and off-target sites were calculated. For Cas12a-mediated genome editing, crRNA scaffold 4n94 and 1 were used separately.

Gene	Target ID	Chromosome	ArCas12a		BsCas12a		HkCas12a		PrCas12a		AsCas12a		SpCas9
		Guide RNA	4n96	1	4n96	1	4n96	1	4n96	1	4n96	1	sgRNA
DNMT1	Target	Chr19	7.7%	6.3%	-	-	7.7%	5.6%	12.5%	4.2%	-	-	14.3%
	Off-target	Chr3	-	-	-	-	-	5.9%	-	-	-	-	-
		Chr5	-	-	-	-	-	-	-	-	-	-	6.3%
		Chr6	-	-	-	-	3.4%	-	-	-	-	-	-
CCR5	Target	Chr3	23.8%	9.1%	33.3%	14.3%	-	-	-	-	25%	12%	46.2%
	Off-target	Chr18	8.3%	-	-	-	-	-	-	-	-	-	-
