# Supplementary material for: Enhanced mammalian genome editing by new Cas12a orthologs with optimized crRNA scaffolds
Source: Genome Biol. 2019 Feb 5;20:15. doi: 10.1186/s13059-019-1620-8 (PMC6362571; doi:10.1186/s13059-019-1620-8)
Supplement: Supplementary file 5 — Supplementary Sequences. The humanized Cas12a coding sequences, protein sequences, U6-crRNA backbone sequences, mammalian and prokaryotic expression vector sequences used in this study. (PDF 183 kb) [file 13059_2019_1620_MOESM5_ESM.pdf]

Supplementary Sequences

Humanized Cas12a coding sequence derived from *Arcobacter butzleri* L348 contig000039 (GeneBank ID: JAIQ01000039.1)

ATGTTCAGCCTGGACTACTTCAGCCTGACCCTGAGCCAGCGCTACATCGACATCTACAACAC
CATGATCGGCGGCAACACCCTGGCCGACGGCACCAAGGTGCAGGGCATCAACGAGAACATCA
ACATCTACCGCCAGAAGAACAACATCGACCGCAAGAACCTGCCCACCCTGAAGCCCCTGCAC
AAGCAGCTGCTGAGCGACCGCGAGACCCTGAGCTGGATCCCCGAGGCCTTCAAGACCAAGGA
GGAGGTGGTGGGCGCCATCGAGGACTTCTACAAGAACAACATCATCAGCTTCAAGTGCTGCG
ACAACATCGTGGACATCACCAAGCAGTTCATCGACATCTTCAGCCTGAACGAGGACTACGAG
CTGAACAAGATCTTCATCAAGAACGACATCAGCATCACCAGCATCAGCCAGGACATCTTCAA
GGACTACCGCATCATCAAGGAGGCCCTGTGGCAGAAGCACATCAACGAGAACCCCAAGGCCG
CCAAGAGCAAGGACCTGACCGGCGACAAGGAGAAGTACTTCAGCCGCAAGAACAGCTTCTTC
AGCTTCGAGGAGATCATCAGCAGCCTGAAGCTGATGGGCCGCAAGATCGACCTGTTCAGCTA
CTTCAAGGACAACGTGGAGTACCGCGCCCACAGCATCGAGACCACCTTCATCAAGTGGCAGA
AGAACAAGAACGACAAGAAGACCACCAAGGAGCTGCTGGACAACATCCTGAACCTGCAGCGC
GTGCTGAAGCCCCTGTACCTGAAGGCCGAGGTGGAGAAGGACATCCTGTTCTACAGCATCTT
CGACATCTACTTCGAGAGCCTGAACGAGATCGTGAAGCTGTACAACAAGGTGCGCGACTTCG
AGAGCAAGAAGCCCTACAGCCTGGAGAAGTTCAAGCTGAACTTCCAGAACAGCACCCTGCTG
AGCGGCTGGGACGTGAACAAGGAGCCCGACAACACCAGCATCCTGCTGAAGAAGGACGGCCT
GTACTACCTGGGCATCATGGACAAGAAGCACAACCGCGTGTTCAAGAACCTGGAGAGCAGCA
AGGGCGGCTACGAGAAGATCGAGTACAAGCTGCTGAGCGGCCCCAACAAGATGCTGCCCAAG
GTGTTCTTCAGCAACAAGAGCATCGGCTACTACAACCCCAGCCCCGCCCTGCTGGAGAAGTA
CAAGAGCGGCGTGCACAAGAAGGGCGAGAGCTTCGACCTGAACTTCTGCCACGAGCTGATCG
ACTTCTTCAAGGCCAGCATCGACAAGCACGAGGACTGGAAGAACTTCAACTTCAAGTTCAGC
GACACCAGCGAGTACGCCGACATCAGCGGCTTCTACCGCGAGGTGGAGCAGCAGGGCTACAA
GATCACCTTCAAGAACATCGACGAGGAGTTCATCAACACCCTGATCAACGAGGGCAAGCTGT
ACCTGTTCCAGATCTACAACAAGGACTTCAGCACCTTCAGCAAGGGCACCAAGAACCTGCAC
ACCCTGTACTGGGAGATGATCTTCAACGAGGAGAACCTGAAGAACGTGGTGTACAAGCTGAA
CGGCGAGGCCGAGATCTTCTACCGCAAGAAGAGCATCGAGTACAGCGAGGACAAGATGAAGT
ACGGCCACCACTACGAGGAGCTGAAGGACAAGTTCAACTACCCCATCATCAAGGACAAGCGC
TTCAccatggACAAGTTCCAGTTCCACGTGCCCATCACCATGAACTTCAAGGCCACCGGCCG
CAGCTACATCAACGAGGAGGTGAACGACTTCCTGCGCCAGAACAGCAAGGACGTGAAGATCA
TCGGCATCAACCGCGGCGAGCGCCACCTGATCTACCTGACCATGATCAACGCCAAGGGCGAG
ATCATCCAGCAGTACAGCCTGAACGAGATCGTGAACAGCTACAACAACAAGAACTTCACCGT
GAACTACAACGAGAAGCTGAGCAAGAAGGAGGGCGAGCGCGCCATCGCCCGCGAGAACTGGG
GCGTGGTGGAGAACATCAAGGAGCTGAAGGAGGGCTACCTGAGCCACGCCATCCACACCATC
AGCAACCTGATCGTGGAGAACAACGCCATCGTGGTGCTGGAGGACCTGAACTTCGAGTTCAA
GCGCGAGCGCCTGAAGGTGGAGAAGAGCATCTACCAGAAGTTCGAGAAGATGCTGATCGACA
AGCTGAACTACCTGGTGGACAAGAAGAAGGACATCAACGAGAACGGCGGCCTGCTGAAGGCC
CTGCAGCTGACCAACAAGTTCGAGAGCTTCGAGAAGATCGGCAAGCAGAACGGCTTCCTGTT
CTTCGTGAACGCCTGGAACATCACCAAGATCTGCCCCGTGACCGGCTTCGTGAGCCTGTTCG
ACACCCGCTACCAGAGCGTGGACAAGGCCCGCGAGTTCTTCAGCAAGTTCGACAGCATCAAG
TACAACGAGGAGAAGGAGCACTACGAGTTCGTGTTCGACTACAGCAACTTCACCGACAAGGC

CAAGGACACCAAGACCAAGTGGACCGTGTGCAGCTACGGCACCCGCATCAAGACCTTCCGCA
ACAGCGAGAAGAACAACAACTGGGACAACAAGACCGTGAGCCCCACCGAGGACCTGAGCAAG
CTGCTGAAGAGCTGCGACCGCGACATCAAGGAGTTCATCATCAGCCAGGACAAGAAGGAGTT
CTTCGTGGAGCTGCTGGAGATCTTCAGCCTGATCGTGCAGATGAAGAACAGCATCATCAACA
GCGAGATCGACTACATCATCAGCCCCGTGGCCAACGAGAACGGCGAGTTCTTCGACAGCCGC
TTCGCCAACAGCAGCCTGCCCAAGAACGCCGACGCCAACGCCGCCTACAACACCGCCCGCAA
GGGCCTGATGCTGCTGGAGAAGATCCGCGACAGCGAGATCGGCAAGAAGATCGACATGAAGA
TCACCAACACCGAGTGGCTGAACTTCGTGCAGGAGCGC

AbCas12a protein sequence

MFSLDYFSLTLSQRYIDIYNTMIGGNTLADGTKVQGINENINIYRQKNNIDRKNLPTLKPLH
KQLLSDRETLSWIPEAFKTKEEVVGAIEDFYKNNIISFKCCDNIVDITKQFIDIFSLNEDYE
LNKIFIKNDISITSISQDIFKDYRIIKEALWQKHINENPKAAKSKDLTGDKEKYFSRKNSFF
SFEEIISSLKLMGRKIDLFSYFKDNVEYRAHSIETTFIKWQKNKNDKKTTKELLDNILNLQR
VLKPLYLKAEVEKDILFYSIFDIYFESLNEIVKLYNKVRDFESKKPYSLEKFKLNFQNSTLL
SGWDVNKEPDNTSILLKKDGLYYLGIMDKKHNRVFKNLESSKGGYEKIEYKLLSGPNKMLPK
VFFSNKSIGYYNPSPALLEKYKSGVHKKGESFDLNFCHELIDFFKASIDKHEDWKNFNFKFS
DTSEYADISGFYREVEQQGYKITFKNIDEEFINTLINEGKLYLFQIYNKDFSTFSKGTKNLH
TLYWEMIFNEENLKNVVYKLNGEAEIFYRKKSIEYSEDKMKYGHHYEELKDKFNYPIIKDKR
FTMDKFQFHVPITMNFKATGRSYINEEVNDFLRQNSKDVKIIGINRGERHLIYLTMINAKGE
IIQQYSLNEIVNSYNNKNFTVNYNEKLSKKEGERAIARENWGVVENIKELKEGYLSHAIHTI
SNLIVENNAIVVLEDLNFEFKRERLKVEKSIYQKFEKMLIDKLNYLVDKKKDINENGGLLKA
LQLTNKFESFEKIGKQNGFLFFVNAWNITKICPVTGFVSLFDTRYQSVDKAREFFSKFDSIK
YNEEKEHYEFVFDYSNFTDKAKDTKTKWTVCSYGTRIKTFRNSEKNNNWDNKTVSPTEDLSK
LLKSCDRDIKEFIISQDKKEFFVELLEIFSLIVQMKNSIINSEIDYIISPVANENGEFFDSR
FANSSLPKNADANAAYNTARKGLMLLEKIRDSEIGKKIDMKITNTEWLNFVQER

Humanized Cas12a coding sequence derived from *Agathobacter rectalis* strain 2789STDY5834884 (GeneBank ID: CZAJ01000001.1)

AACAACGGCACAAACAATTTCCAGAACTTCATTGGCATTTCATCACTCCAGAAGACCCTGAG
GAATGCCCTGACACCTACCGAGACAACCCAGCAATTCATTGTGAAGAACGGAATCATTAAGG
AGGACGAACTGAGAGGCGAGAACCGCCAGATCCTGAAAGACATCATGGACGACTACTATCGC
GGTTTCATCTCCGAGACACTCAGCAGCATTGATGATATTGATTGGACATCTCTGTTTGAGAA
GATGGAAATCCAGCTGAAGAATGGCGACAACAAGGACACTCTCATTAAGGAGCAAGCAGAGA
AGAGAAAGGCTATCTACAAGAAGTTTGCAGACGACGATCGCTTCAAGAATATGTTTAGCGCA
AAGCTGATAAGTGATATTCTCCCAGAGTTTGTGATTCACAACAACAACTATTCTGCCAGCGA
GAAGGAAGAGAAGACACAGGTCATCAAGCTCTTCTCTCGGTTTGCCACCTCATTCAAAGATT
ACTTCAAGAATCGGGCAAATTGTTTCTCTGCCGATGACATCTCATCTTCCTCCTGTCACAGA
ATCGTTAATGATAACGCCGAAATCTTCTTCTCAAATGCCCTGGTGTACCGGAGAATCGTGAA
GAATCTGAGTAACGACGACATCAATAAGATTAGCGGTGATATGAAGGACTCTCTGAAGAAGA
TGTCCCTGGAGAAGATATACAGCTATGAGAAGTACGGCGAGTTCATCACTCAAGAGGGAATT
AGCTTCTACAACGACATTTGTGGCAAGGTTAACTCATTCATGAATCTCTACTGCCAGAAGAA
CAAGGAGAATAAGAATCTGTACAAACTGCGCAAACTGCATAAGCAAATCCTGTGTATCGCCG
ACACCTCTTATGAGGTGCCCTACAAGTTTGAGTCTGACGAAGAGGTGTACCAGTCCGTGAAC

GGATTCCTGGACAACATCAGTTCTAAACACATCGTCGAGCGGCTGAGGAAGATCGGCGACAA
CTACAATGGCTATAACCTGGACAAGATATACATCGTCAGTAAGTTCTATGAAAGTGTGAGTC
AGAAGACCTACCGGGACTGGGAGACTATCAACACCGCACTCGAAATCCACTACAATAACATC
CTGCCTGGCAACGGTAAGAGCAAGGCCGACAAGGTGAAGAAGGCCGTCAAGAACGACCTCCA
GAAGAGCATCACCGAGATTAACGAACTGGTGAGTAACTACAAGCTCTGCCCAGACGATAACA
TTAAGGCAGAAACCTACATTCATGAGATTTCCCATATACTGAACAACTTTGAAGCTCAAGAG
CTGAAATACAATCCCGAGATTCATCTGGTGGAGAGCGAGCTGAAAGCATCCGAGCTGAAGAA
CGTTCTCGACGTCATCATGAACGCCTTCCACTGGTGTAGCGTGTTCATGACTGAGGAGCTGG
TCGATAAAGACAACAATTTCTACGCCGAGCTGGAAGAAATCTACGACGAGATATACCCTGTG
ATTAGCCTCTACAATCTGGTCCGGAACTATGTGACCCAGAAGCCCTATTCAACTAAGAAGAT
CAAGCTGAACTTTGGGATTCCTACCCTGGCCGACGGCTGGTCCAAGAGCAAAGAGTATTCCA
ATAACGCAATCATCCTGATGAGAGACAACCTGTATTACCTCGGTATCTTTAACGCTAAGAAT
AAGCCCGAGAAGAAGATCATCGAAGGAAATACATCCGAGAACAAGGGCGACTACAAGAAGAT
GATCTATAACCTGCTGCCTGGCCCAAACAAGATGATCCCTAAGGTGTTCCTGAGCAGCAAGA
CCGGAGTCGAGACTTACAAGCCAAGTGCCTACATACTGGAGGGCTATAAGCAGAACAAGCAC
CTGAAATCTAGCAAAGATTTCGACATCACTTTCTGTCGCGACCTGATCGACTATTTCAAGAA
TTGTATTGCCATCCACCCAGAGTGGAAGAATTTCGGATTCGACTTCTCTGACACCTCCACAT
ACGAGGACATCAGTGGCTTCTATAGAGAAGTGGAGCTGCAAGGTTACAAGATCGACTGGACC
TACATATCTGAGAAAGACATCGACCTGCTGCAAGAGAAAGGGCAGCTCTACCTCTTCCAAAT
CTACAACAAGGACTTTAGTAAGAAGTCTACAGGTAATGACAATCTGCACACTATGTACCTGA
AGAATCTCTTCTCTGAAGAGAACCTCAAAGACGTGGTGCTGAAACTGAACGGCGAAGCAGAA
ATCTTCTTTCGCAAATCATCCATTAAGAATCCTATCATACACAAGAAGGGTAGTATCCTGGT
GAACAGGACATACGAAGCCGAGGAGAAAGATCAGTTCGGCAACATTCAGATTGTGCGCAAGA
CTATTCCCGAGAATATCTACCAGGAGCTGTACAAATACTTCAACGATAAGTCTGATAAAGAG
CTGTCAGACGAAGCAGCCAAGCTGAAGAATGCTGTGGGACATCATGAAGCAGCTACTAACAT
CGTGAAAGACTATAGATACACATACGACAAGTATTTCCTGCACATGCCAATTACCATCAACT
TCAAAGCCAATAAGACTTCTTTCATTAACGACCGCATCCTCCAGTACATTGCAAAGGAGAAA
GACCTGCACGTGATCGGGATTGATCGCGGAGAACGGAACCTCATCTACGTTTCAGTCATCGA
CACATGCGGTAACATCGTCGAACAGAAGAGCTTCAACATTGTTAATGGGTATGATTACCAGA
TCAAACTCAAGCAACAGGAAGGCGCACGGCAGATTGCTCGCAAGGAGTGGAAGGAAATTGGC
AAGATCAAGGAAATCAAGGAAGGATACCTCAGCCTCGTCATTCATGAAATCAGCAAGATGGT
GATCAAGTATAACGCAATCATCGCTATGGAGGACCTGAGTTATGGCTTCAAGAAAGGCAGAT
TCAAGGTGGAGCGGCAAGTCTACCAGAAATTCGAAACAATGCTGATCAACAAGCTGAACTAC
CTGGTGTTCAAAGACATCAGCATAACCGAGAACGGAGGACTCCTGAAAGGCTACCAGCTCAC
ATACATCCCAGAGAAACTCAAGAATGTGGGCCACCAATGCGGCTGCATCTTCTACGTCCCTG
CCGCTTACACCAGCAAGATAGATCCCACTACAGGATTCGTGAACATATTCAAATTCAAGGAT
CTGACAGTGGACGCCAAGAGGGAGTTCATCAAGAAGTTTGATAGTATTCGCTATGACAGCGA
TAAGAATCTGTTCTGTTTCACCTTTGACTACAACAACTTCATTACCCAGAATACCGTTATGT
CCAAGTCTAGCTGGAGTGTGTATACCTACGGTGTTCGGATCAAGCGGAGGTTTGTCAATGGT
AGATTCTCAAACGAAAGCGACACCATCGACATCACAAAGGACATGGAGAAGACACTGGAAAT
GACTGACATAAACTGGAGAGATGGACACGACCTGCGGCAAGACATCATTGACTACGAGATCG
TTCAGCACATCTTTGAAATCTTCAAGCTGACTGTTCAGATGCGGAATAGTCTGAGCGAGCTG
GAGGACCGGAATTACGACCGCCTGATCTCACCAGTCCTGAACGAGAATAACATCTTCTACGA
TTCTGCCAAAGCAGGAGATGCCCTGCCAAAGGACGCTGATGCAAACGGTGCCTACTGCATCG

CCCTCAAAGGTCTGTACGAAATCAAGCAGATTACCGAGAATTGGAAGGAGGACGGGAAGTTC
AGCAGAGACAAGCTCAAGATCAGCAATAAGGACTGGTTCGATTTCATTCAGAACAAGCGCTA
CCTG

ArCas12a protein sequence

NNGTNNFQNFIGISSLQKTLRNALTPTETTQQFIVKNGIIKEDELRGENRQILKDIMDDYYR
GFISETLSSIDDIDWTSLFEKMEIQLKNGDNKDTLIKEQAEKRKAIYKKFADDDRFKNMFSA
KLISDILPEFVIHNNNYSASEKEEKTQVIKLFSRFATSFKDYFKNRANCFSADDISSSSCHR
IVNDNAEIFFSNALVYRRIVKNLSNDDINKISGDMKDSLKKMSLEKIYSYEKYGEFITQEGI
SFYNDICGKVNSFMNLYCQKNKENKNLYKLRKLHKQILCIADTSYEVPYKFESDEEVYQSVN
GFLDNISSKHIVERLRKIGDNYNGYNLDKIYIVSKFYESVSQKTYRDWETINTALEIHYNNI
LPGNGKSKADKVKKAVKNDLQKSITEINELVSNYKLCPDDNIKAETYIHEISHILNNFEAQE
LKYNPEIHLVESELKASELKNVLDVIMNAFHWCSVFMTEELVDKDNNFYAELEEIYDEIYPV
ISLYNLVRNYVTQKPYSTKKIKLNFGIPTLADGWSKSKEYSNNAIILMRDNLYYLGIFNAKN
KPEKKIIEGNTSENKGDYKKMIYNLLPGPNKMIPKVFLSSKTGVETYKPSAYILEGYKQNKH
LKSSKDFDITFCRDLIDYFKNCIAIHPEWKNFGFDFSDTSTYEDISGFYREVELQGYKIDWT
YISEKDIDLLQEKGQLYLFQIYNKDFSKKSTGNDNLHTMYLKNLFSEENLKDVVLKLNGEAE
IFFRKSSIKNPIIHKKGSILVNRTYEAEEKDQFGNIQIVRKTIPENIYQELYKYFNDKSDKE
LSDEAAKLKNAVGHHEAATNIVKDYRYTYDKYFLHMPITINFKANKTSFINDRILQYIAKEK
DLHVIGIDRGERNLIYVSVIDTCGNIVEQKSFNIVNGYDYQIKLKQQEGARQIARKEWKEIG
KIKEIKEGYLSLVIHEISKMVIKYNAIIAMEDLSYGFKKGRFKVERQVYQKFETMLINKLNY
LVFKDISITENGGLLKGYQLTYIPEKLKNVGHQCGCIFYVPAAYTSKIDPTTGFVNIFKFKD
LTVDAKREFIKKFDSIRYDSDKNLFCFTFDYNNFITQNTVMSKSSWSVYTYGVRIKRRFVNG
RFSNESDTIDITKDMEKTLEMTDINWRDGHDLRQDIIDYEIVQHIFEIFKLTVQMRNSLSEL
EDRNYDRLISPVLNENNIFYDSAKAGDALPKDADANGAYCIALKGLYEIKQITENWKEDGKF
SRDKLKISNKDWFDFIQNKRYL

Humanized Cas12a coding sequence derived from *Bacteroidetes oral* taxon 274 str. F0058 (GeneBank ID: NZ_GG774890.1)

AGGAAATTCAATGAGTTCGTGGGTCTGTATCCTATTAGTAAGACCCTCAGGTTCGAGCTGAA
ACCAATCGGCAAGACACTGGAGCATATCCAGAGAAACAAGCTCCTGGAGCATGATGCCGTTC
GCGCTGACGACTATGTCAAAGTGAAGAAGATCATTGACAAATACCATAAGTGTCTGATAGAT
GAGGCCCTGTCTGGATTCACCTTCGATACAGAAGCCGATGGGAGAAGCAATAACAGCCTGTC
TGAGTACTATCTGTACTACAATCTCAAGAAGAGAAATGAGCAGGAACAGAAGACTTTCAAGA
CAATCCAGAACAATCTGCGGAAGCAGATTGTCAACAAGCTGACCCAGAGTGAGAAGTATAAG
AGAATTGATAAGAAAGAACTCATCACCACTGATCTGCCAGACTTCCTGACTAATGAAAGCGA
GAAAGAACTGGTGGAGAAGTTCAAGAACTTTACTACCTACTTTACCGAATTTCACAAGAACC
GCAAGAATATGTACTCCAAGGAAGAGAAGTCCACCGCAATCGCTTTCCGCCTGATTAACGAG
AACCTGCCAAAGTTTGTCGATAACATCGCTGCTTTCGAGAAAGTTGTGTCCTCACCTCTCGC
AGAGAAGATCAATGCCCTGTACGAGGACTTTAAGGAGTATCTGAATGTGGAAGAAATCTCAC
GGGTGTTTAGACTCGACTATTACGATGAACTGCTGACACAGAAACAGATTGATCTGTACAAC
GCTATCGTCGGTGGTCGGACAGAGGAGGACAACAAGATCCAGATAAAGGGACTGAACCAGTA
TATCAACGAATACAATCAGCAGCAGACAGATCGGTCTAATCGGCTGCCAAAGCTGAAACCTC
TCTATAAGCAAATTCTCTCCGACAGAGAGAGCGTGTCATGGCTGCCTCCCAAGTTTGATAGC

GATAAGAATCTGCTGATTAAGATCAAAGAATGCTACGACGCCCTGTCCGAGAAGGAGAAAGT
GTTTGACAAGCTGGAAAGTATTCTCAAGAGCCTGTCAACCTATGACCTGTCTAAGATATACA
TTTCTAACGACTCTCAGCTGTCTTACATTAGCCAGAAGATGTTTGGACGGTGGGACATCATA
TCTAAGGCCATCAGGGAGGATTGTGCTAAGAGGAATCCTCAGAAATCTCGGGAATCCCTGGA
GAAGTTCGCCGAGAGGATAGATAAGAAACTCAAGACCATCGACTCCATCAGCATCGGCGATG
TGGATGAGTGCCTGGCCCAGCTGGGTGAAACCTACGTTAAGCGGGTGGAGGATTACTTTGTG
GCAATGGGCGAATCCGAGATCGACGATGAGCAGACAGATACCACCTCCTTCAAGAAGAACAT
AGAGGGAGCATACGAGTCCGTCAAGGAGCTGCTGAACAACGCTGATAACATTACAGACAATA
ACCTGATGCAGGACAAGGGCAATGTGGAGAAGATCAAGACCCTGCTGGATGCAATCAAGGAC
CTCCAGCGGTTCATTAAGCCACTCCTGGGTAAAGGTGACGAAGCAGACAAGGACGGCGTGTT
CTACGGTGAGTTTACATCCCTGTGGACCAAACTCGATCAGGTTACTCCTCTCTATAACATGG
TTCGGAATTACCTCACTTCAAAGCCTTATAGTACAAAGAAGATTAAGCTGAACTTTGAGAAC
AGCACTCTCATGGATGGATGGGATCTGAATAAGGAGCCAGATAACACTACCGTGATATTCTG
CAAAGATGGGCTGTATTACCTGGGCATTATGGGTAAGAAGTACAATAGAGTGTTTGTCGATA
GAGAGGACCTGCCTCACGACGGCGAGTGCTACGACAAGATGGAGTACAAACTGCTGCCAGGT
GCCAATAAGATGCTCCCTAAAGTGTTCTTCTCCGAAACTGGTATTCAACGGTTCCTCCCATC
CGAGGAACTCCTGGGCAAGTACGAAAGAGGCACACATAAGAAAGGAGCTGGGTTTGACCTGG
GAGACTGTAGAGCACTGATTGATTTCTTTAAGAAGAGCATTGAAAGGCACGATGATTGGAAG
AAGTTTGACTTCAAGTTCAGCGACACAAGCACATACCAGGACATAAGTGAGTTCTATAGAGA
AGTGGAGCAGCAGGGCTATAAGATGTCCTTTAGAAAGGTTTCTGTGGACTATATCAAGTCTC
TGGTGGAAGAAGGTAAGCTGTATCTGTTCCAGATATACAACAAAGACTTCTCCGCACATTCC
AAAGGGACACCTAACATGCACACTCTCTATTGGAAGATGCTGTTCGATGAGGAGAACCTGAA
GGACGTGGTGTATAAGCTGAATGGAGAAGCTGAGGTGTTCTTCCGGAAATCTAGCATCACAG
TGCAAAGCCCAACACACCCTGCTAATTCACCTATCAAGAACAAGAACAAGGATAATCAGAAG
AAGGAATCAAAGTTTGAGTACGATCTCATCAAGGACCGCAGGTATACCGTGGACAAGTTCCT
CTTTCACGTGCCTATAACCATGAATTTCAAGTCCGTCGGTGGCTCTAACATCAATCAGCTCG
TGAAGCGGCACATTCGGTCCGCAACCGACCTCCACATCATCGGCATAGATAGAGGAGAGCGG
CATCTGCTGTACCTGACCGTTATCGACAGCAGAGGTAACATCAAAGAACAGTTCAGTCTGAA
CGAGATAGTGAACGAGTATAACGGGAACACCTATCGGACCGATTACCACGAGCTGCTCGATA
CCAGAGAAGGCGAGAGAACAGAAGCTAGACGGAACTGGCAGACTATACAGAACATACGCGAG
CTGAAAGAGGGATACCTCTCCCAGGTGATTCACAAGATCAGCGAGCTGGCTATCAAATACAA
CGCCGTGATCGTGCTGGAGGATCTCAATTTCGGCTTTATGAGGTCACGCCAGAAAGTGGAGA
AGCAGGTGTATCAGAAATTCGAGAAGATGCTGATCGACAAGCTGAACTACCTGGTCGATAAG
AAGAAACCTGTCGCTGAAACCGGAGGGCTGCTGAGAGCCTACCAGCTGACCGGAGAATTTGA
GTCCTTTAAGACCCTGGGAAAGCAGAGCGGCATTCTGTTCTACGTTCCCGCTTGGAACACCA
GTAAGATTGATCCTGTGACTGGGTTTGTCAATCTCTTCGATACCCACTATGAGAACATTGAG
AAGGCTAAGGTGTTCTTTGACAAATTCAAGAGTATCAGGTACAATTCCGACAAGGATTGGTT
CGAATTTGTCGTGGACGACTATACAAGGTTCTCACCTAAGGCAGAGGGCACCAGGAGGGACT
GGACTATCTGCACCCAGGGAAAGCGCATTCAGATATGTCGGAACCACCAGCGCAATAACGAG
TGGGAGGGTCAAGAGATTGACCTGACCAAAGCATTCAAGGAGCACTTTGAAGCCTATGGCGT
TGACATCTCAAAGGACCTGAGGGAGCAGATCAATACTCAGAACAAGAAAGAGTTCTTCGAAG
AACTGCTGCGCCTGCTGCGGCTCACCCTGCAAATGAGGAACTCCATGCCAAGTTCTGACATC
GACTACCTGATCAGCCCAGTCGCCAACGACACCGGATGCTTCTTCGATTCAAGAAAGCAGGC
CGAGCTGAAAGAGAATGCAGTTCTCCCTATGAACGCTGATGCTAATGGTGCATACAACATCG

CTAGAAAGGGACTGCTGGCAATCCGCAAGATGAAACAAGAAGAGAACGACAGTGCTAAGATC
AGCCTCGCTATATCCAACAAGGAGTGGCTCAAGTTTGCTCAGACTAAGCCATATCTGGAGGA
C

BoCas12a protein sequence

RKFNEFVGLYPISKTLRFELKPIGKTLEHIQRNKLLEHDAVRADDYVKVKKIIDKYHKCLID
EALSGFTFDTEADGRSNNSLSEYYLYYNLKKRNEQEQKTFKTIQNNLRKQIVNKLTQSEKYK
RIDKKELITTDLPDFLTNESEKELVEKFKNFTTYFTEFHKNRKNMYSKEEKSTAIAFRLINE
NLPKFVDNIAAFEKVVSSPLAEKINALYEDFKEYLNVEEISRVFRLDYYDELLTQKQIDLYN
AIVGGRTEEDNKIQIKGLNQYINEYNQQQTDRSNRLPKLKPLYKQILSDRESVSWLPPKFDS
DKNLLIKIKECYDALSEKEKVFDKLESILKSLSTYDLSKIYISNDSQLSYISQKMFGRWDII
SKAIREDCAKRNPQKSRESLEKFAERIDKKLKTIDSISIGDVDECLAQLGETYVKRVEDYFV
AMGESEIDDEQTDTTSFKKNIEGAYESVKELLNNADNITDNNLMQDKGNVEKIKTLLDAIKD
LQRFIKPLLGKGDEADKDGVFYGEFTSLWTKLDQVTPLYNMVRNYLTSKPYSTKKIKLNFEN
STLMDGWDLNKEPDNTTVIFCKDGLYYLGIMGKKYNRVFVDREDLPHDGECYDKMEYKLLPG
ANKMLPKVFFSETGIQRFLPSEELLGKYERGTHKKGAGFDLGDCRALIDFFKKSIERHDDWK
KFDFKFSDTSTYQDISEFYREVEQQGYKMSFRKVSVDYIKSLVEEGKLYLFQIYNKDFSAHS
KGTPNMHTLYWKMLFDEENLKDVVYKLNGEAEVFFRKSSITVQSPTHPANSPIKNKNKDNQK
KESKFEYDLIKDRRYTVDKFLFHVPITMNFKSVGGSNINQLVKRHIRSATDLHIIGIDRGER
HLLYLTVIDSRGNIKEQFSLNEIVNEYNGNTYRTDYHELLDTREGERTEARRNWQTIQNIRE
LKEGYLSQVIHKISELAIKYNAVIVLEDLNFGFMRSRQKVEKQVYQKFEKMLIDKLNYLVDK
KKPVAETGGLLRAYQLTGEFESFKTLGKQSGILFYVPAWNTSKIDPVTGFVNLFDTHYENIE
KAKVFFDKFKSIRYNSDKDWFEFVVDDYTRFSPKAEGTRRDWTICTQGKRIQICRNHQRNNE
WEGQEIDLTKAFKEHFEAYGVDISKDLREQINTQNKKEFFEELLRLLRLTLQMRNSMPSSDI
DYLISPVANDTGCFFDSRKQAELKENAVLPMNADANGAYNIARKGLLAIRKMKQEENDSAKI
SLAISNKEWLKFAQTKPYLED

Humanized Cas12a coding sequence derived from *Butyrivibrio sp.* NC3005 (GeneBank ID: NZ_AUKC01000013.1)

ATGTACTACCAGAACCTGACCAAGAAGTACCCCGTGAGCAAGACCATCCGCAACGAGCTGAT
CCCCATCGGCAAGACCCTGGAGAACATCCGCAAGAACAACATCCTGGAGAGCGACGTGAAGC
GCAAGCAGGACTACGAGCACGTGAAGGGCATCATGGACGAGTACCACAAGCAGCTGATCAAC
GAGGCCCTGGACAACTACATGCTGCCCAGCCTGAACCAGGCCGCCGAGATCTACCTGAAGAA
GCACGTGGACGTGGAGGACCGCGAGGAGTTCAAGAAGACCCAGGACCTGCTGCGCCGCGAGG
TGACCGGCCGCCTGAAGGAGCACGAGAACTACACCAAGATCGGCAAGAAGGACATCCTGGAC
CTGCTGGAGAAGCTGCCCAGCATCAGCGAGGAGGACTACAACGCCCTGGAGAGCTTCCGCAA
CTTCTACACCTACTTCACCAGCTACAACAAGGTGCGCGAGAACCTGTACAGCGACGAGGAGA
AGAGCAGCACCGTGGCCTACCGCCTGATCAACGAGAACCTGCCCAAGTTCCTGGACAACATC
AAGAGCTACGCCTTCGTGAAGGCCGCCGGCGTGCTGGCCGACTGCATCGAGGAGGAGGAGCA
GGACGCCCTGTTCATGGTGGAGACCTTCAACATGACCCTGACCCAGGAGGGCATCGACATGT
ACAACTACCAGATCGGCAAGGTGAACAGCGCCATCAACCTGTACAACCAGAAGAACCACAAG
GTGGAGGAGTTCAAGAAGATCCCCAAGATGAAGGTGCTGTACAAGCAGATCCTGAGCGACCG
CGAGGAGGTGTTCATCGGCGAGTTCAAGGACGACGAGACCCTGCTGAGCAGCATCGGCGCCT
ACGGCAACGTGCTGATGACCTACCTGAAGAGCGAGAAGATCAACATCTTCTTCGACGCCCTG

CGCGAGAGCGAGGGCAAGAACGTGTACGTGAAGAACGACCTGAGCAAGACCACCATGAGCAA
CATCGTGTTCGGCAGCTGGAGCGCCTTCGACGAGCTGCTGAACCAGGAGTACGACCTGGCCA
ACGAGAACAAGAAGAAGGACGACAAGTACTTCGAGAAGCGCCAGAAGGAGCTGAAGAAGAAC
AAGAGCTACACCCTGGAGCAGATGAGCAACCTGAGCAAGGAGGACATCAGCCCCATCGAGAA
CTACATCGAGCGCATCAGCGAGGACATCGAGAAGATCTGCATCTACAACGGCGAGTTCGAGA
AGATCGTGGTGAACGAGCACGACAGCAGCCGCAAGCTGAGCAAGAACATCAAGGCCGTGAAG
GTGATCAAGGACTACCTGGACAGCATCAAGGAGCTGGAGCACGACATCAAGCTGATCAACGG
CAGCGGCCAGGAGCTGGAGAAGAACCTGGTGGTGTACGTGGGCCAGGAGGAGGCCCTGGAGC
AGCTGCGCCCCGTGGACAGCCTGTACAACCTGACCCGCAACTACCTGACCAAGAAGCCCTTC
AGCACCGAGAAGGTGAAGCTGAACTTCAACAAGAGCACCCTGCTGAACGGCTGGGACAAGAA
CAAGGAGACCGACAACCTGGGCATCCTGTTCTTCAAGGACGGCAAGTACTACCTGGGCATCA
TGAACACCACCGCCAACAAGGCCTTCGTGAACCCCCCCGCCGCCAAGACCGAGAACGTGTTC
AAGAAGGTGGACTACAAGCTGCTGCCCGGCAGCAACAAGATGCTGCCCAAGGTGTTCTTCGC
CAAGAGCAACATCGGCTACTACAACCCCAGCACCGAGCTGTACAGCAACTACAAGAAGGGCA
CCCACAAGAAgggcccCAGCTTCAGCATCGACGACTGCCACAACCTGATCGACTTCTTCAAG
GAGAGCATCAAGAAGCACGAGGACTGGAGCAAGTTCGGCTTCGAGTTCAGCGACACCGCCGA
CTACCGCGACATCAGCGAGTTCTACCGCGAGGTGGAGAAGCAGGGCTACAAGCTGACCTTCA
CCGACATCGACGAGAGCTACATCAACGACCTGATCGAGAAGAACGAGCTGTACCTGTTCCAG
ATCTACAACAAGGACTTCAGCGAGTACAGCAAGGGCAAGCTGAACCTGCACACCCTGTACTT
CATGATGCTGTTCGACCAGCGCAACCTGGACAACGTGGTGTACAAGCTGAACGGCGAGGCCG
AGGTGTTCTACCGCCCCGCCAGCATCGCCGAGAACGAGCTGGTGATCCACAAGGCCGGCGAG
GGCATCAAGAACAAGAACCCCAACCGCGCCAAGGTGAAGGAGACCAGCACCTTCAGCTACGA
CATCGTGAAGGACAAGCGCTACAGCAAGTACAAGTTCACCCTGCACATCCCCATCACCATGA
ACTTCGGCGTGGACGAGGTGCGCCGCTTCAACGACGTGATCAACAACGCCCTGCGCACCGAC
GACAACGTGAACGTGATCGGCATCGACCGCGGCGAGCGCAACCTGCTGTACGTGGTGGTGAT
CAACAGCGAGGGCAAGATCCTGGAGCAGATCAGCCTGAACAGCATCATCAACAAGGAGTACG
ACATCGAGACCAACTACCACGCCCTGCTGGACGAGCGCGAGGACGACCGCAACAAGGCCCGC
AAGGACTGGAACACCATCGAGAACATCAAGGAGCTGAAGACCGGCTACCTGAGCCAGGTGGT
GAACGTGGTGGCCAAGCTGGTGCTGAAGTACAACGCCATCATCTGCCTGGAGGACCTGAACT
TCGGCTTCAAGCGCGGCCGCCAGAAGGTGGAGAAGCAGGTGTACCAGAAGTTCGAGAAGATG
CTGATCGAGAAGCTGAACTACCTGGTGATCGACAAGAGCCGCGAGCAGGTGAGCCCCGAGAA
GATGGGCGGCGCCCTGAACGCCCTGCAGCTGACCAGCAAGTTCAAGAGCTTCGCCGAGCTGG
GCAAGCAGAGCGGCATCATCTACTACGTGCCCGCCTACCTGACCAGCAAGATCGACCCCACC
ACCGGCTTCGTGAACCTGTTCTACATCAAGTACGAGAACATCGAGAAGGCCAAGCAGTTCTT
CGACGGCTTCGACTTCATCCGCTTCAACAAGAAGGACGACATGTTCGAGTTCAGCTTCGACT
ACAAGAGCTTCACCCAGAAGGCCTGCGGCATCCGCAGCAAGTGGATCGTGTACACCAACGGC
GAGCGCATCATCAAGTACCCCAACCCCGAGAAGAACAACCTGTTCGACGAGAAGGTGATCAA
CGTGACCGACGAGATCAAGGGCCTGTTCAAGCAGTACCGCATCCCCTACGAGAACGGCGAGG
ACATCAAGGAGATCATCATCAGCAAGGCCGAGGCCGACTTCTACAAGCGCCTGTTCCGCCTG
CTGCACCAGACCCTGCAGATGCGCAACAGCACCAGCGACGGCACCCGCGACTACATCATCAG
CCCCGTGAAGAACGACCGCGGCGAGTTCTTCTGCAGCGAGTTCAGCGAGGGCACCATGCCCA
AGGACGCCGACGCCAACGGCGCCTACAACATCGCCCGCAAGGGCCTGTGGGTGCTGGAGCAG
ATCCGCCAGAAGGACGAGGGCGAGAAGGTGAACCTGAGCATGACCAACGCCGAGTGGCTGAA
GTACGCCCAGCTGCACCTGCTG

BsCas12a protein sequence

MYYQNLTKKYPVSKTIRNELIPIGKTLENIRKNNILESDVKRKQDYEHVKGIMDEYHKQLIN
EALDNYMLPSLNQAAEIYLKKHVDVEDREEFKKTQDLLRREVTGRLKEHENYTKIGKKDILD
LLEKLPSISEEDYNALESFRNFYTYFTSYNKVRENLYSDEEKSSTVAYRLINENLPKFLDNI
KSYAFVKAAGVLADCIEEEEQDALFMVETFNMTLTQEGIDMYNYQIGKVNSAINLYNQKNHK
VEEFKKIPKMKVLYKQILSDREEVFIGEFKDDETLLSSIGAYGNVLMTYLKSEKINIFFDAL
RESEGKNVYVKNDLSKTTMSNIVFGSWSAFDELLNQEYDLANENKKKDDKYFEKRQKELKKN
KSYTLEQMSNLSKEDISPIENYIERISEDIEKICIYNGEFEKIVVNEHDSSRKLSKNIKAVK
VIKDYLDSIKELEHDIKLINGSGQELEKNLVVYVGQEEALEQLRPVDSLYNLTRNYLTKKPF
STEKVKLNFNKSTLLNGWDKNKETDNLGILFFKDGKYYLGIMNTTANKAFVNPPAAKTENVF
KKVDYKLLPGSNKMLPKVFFAKSNIGYYNPSTELYSNYKKGTHKKGPSFSIDDCHNLIDFFK
ESIKKHEDWSKFGFEFSDTADYRDISEFYREVEKQGYKLTFTDIDESYINDLIEKNELYLFQ
IYNKDFSEYSKGKLNLHTLYFMMLFDQRNLDNVVYKLNGEAEVFYRPASIAENELVIHKAGE
GIKNKNPNRAKVKETSTFSYDIVKDKRYSKYKFTLHIPITMNFGVDEVRRFNDVINNALRTD
DNVNVIGIDRGERNLLYVVVINSEGKILEQISLNSIINKEYDIETNYHALLDEREDDRNKAR
KDWNTIENIKELKTGYLSQVVNVVAKLVLKYNAIICLEDLNFGFKRGRQKVEKQVYQKFEKM
LIEKLNYLVIDKSREQVSPEKMGGALNALQLTSKFKSFAELGKQSGIIYYVPAYLTSKIDPT
TGFVNLFYIKYENIEKAKQFFDGFDFIRFNKKDDMFEFSFDYKSFTQKACGIRSKWIVYTNG
ERIIKYPNPEKNNLFDEKVINVTDEIKGLFKQYRIPYENGEDIKEIIISKAEADFYKRLFRL
LHQTLQMRNSTSDGTRDYIISPVKNDRGEFFCSEFSEGTMPKDADANGAYNIARKGLWVLEQ
IRQKDEGEKVNLSMTNAEWLKYAQLHLL

Humanized Cas12a coding sequence derived from Candidate division WS6 bacterium GW2011_GWA2_37_6 US52_C0007 (GeneBank ID: LBTH01000007.1)

ATGAAGAACGTGTTCGGCGGCTTCACCAACCTGTACAGCCTGACCAAGACCCTGCGCTTCGA
GCTGAAGCCCACCAGCAAGACCCAGAAGCTGATGAAGCGCAACAACGTGATCCAGACCGACG
AGGAGATCGACAAGCTGTACCACGACGAGATGAAGCCCATCCTGGACGAGATCCACCGCCGC
TTCATCAACGACGCCCTGGCCCAGAAGATCTTCATCAGCGCCAGCCTGGACAACTTCCTGAA
GGTGGTGAAGAACTACAAGGTGGAGAGCGCCAAGAAGAACATCAAGCAGAACCAGGTGAAGC
TGCTGCAGAAGGAGATCACCATCAAGACCCTGGGCCTGCGCCGCGAGGTGGTGAGCGGCTTC
ATCACCGTGAGCAAGAAGTGGAAGGACAAGTACGTGGGCCTGGGCATCAAGCTGAAGGGCGA
CGGCTACAAGGTGCTGACCGAGCAGGCCGTGCTGGACATCCTGAAGATCGAGTTCCCCAACA
AGGCCAAGTACATCGACAAGTTCCGCGGCTTCTGGACCTACTTCAGCGGCTTCAACGAGAAC
CGCAAGAACTACTACAGCGAGGAGGACAAGGCCACCAGCATCGCCAACCGCATCGTGAACGA
GAACCTGAGCCGCTACATCGACAACATCATCGCCTTCGAGGAGATCCTGCAGAAGATCCCCA
ACCTGAAGAAGTTCAAGCAGGACCTGGACATCACCAGCTACAACTACTACCTGAACCAGGCC
GGCATCGACAAGTACAACAAGATCATCGGCGGCTACATCGTGGACAAGGACAAGAAGATCCA
GGGCATCAACGAGAAGGTGAACCTGTACACCCAGCAGACCAAGAAGAAGCTGCCCAAGCTGA
AGTTCCTGTTCAAGCAGATCGGCAGCGAGCGCAAGGGCTTCGGCATCTTCGAGATCAAGGAG
GGCAAGGAGTGGGAGCAGCTGGGCGACCTGTTCAAGCTGCAGCGCACCAAGATCAACAGCAA
CGGCCGCGAGAAGGGCCTGTTCGACAGCCTGCGCACCATGTACCGCGAGTTCTTCGACGAGA
TCAAGCGCGACAGCAACAGCCAGGCCCGCTACAGCCTGGACAAGATCTACTTCAACAAGGCC
AGCGTGAACACCATCAGCAACAGCTGGTTCACCAACTGGAACAAGTTCGCCGAGCTGCTGAA

CATCAAGGAGGACAAGAAGAACGGCGAGAAGAAGATCCCCGAGCAGATCAGCATCGAGGACA
TCAAGGACAGCCTGAGCATCATCCCCAAGGAGAACCTGGAGGAGCTGTTCAAGCTGACCAAC
CGCGAGAAGCACGACCGCACCCGCTTCTTCGGCAGCAACGCCTGGGTGACCTTCCTGAACAT
CTGGCAGAACGAGATCGAGGAGAGCTTCAACAAGCTGGAGGAGAAGGAGAAGGACTTCAAGA
AGAACGCCGCCATCAAGTTCCAGAAGAACAACCTGGTGCAGAAGAACTACATCAAGGAGGTG
TGCGACCgcatgcTGGCCATCGAGCGCATGGCCAAGTACCACCTGCCCAAGGACAGCAACCT
GAGCCGCGAGGAGGACTTCTACTGGATCATCGACAACCTGAGCGAGCAGCGCGAGATCTACA
AGTACTACAACGCCTTCCGCAACTACATCAGCAAGAAGCCCTACAACAAGAGCAAGATGAAG
CTGAACTTCGAGAACGGCAACCTGCTGGGCGGCTGGAGCGACGGCCAGGAGCGCAACAAGGC
CGGCGTGATCCTGCGCAACGGCAACAAGTACTACCTGGGCGTGCTGATCAACCGCGGCATCT
TCCGCACCGACAAGATCAACAACGAGATCTACCGCACCGGCAGCAGCAAGTGGGAGCGCCTG
ATCCTGAGCAACCTGAAGTTCCAGACCCTGGCCGGCAAGGGCTTCCTGGGCAAGCACGGCGT
GAGCTACGGCAACATGAACCCCGAGAAGAGCGTGCCCAGCCTGCAGAAGTTCATCCGCGAGA
ACTACCTGAAGAAGTACCCCCAGCTGACCGAGGTGAGCAACACCAAGTTCCTGAGCAAGAAG
GACTTCGACGCCGCCATCAAGGAGGCCCTGAAGGAGTGCTTCACCATGAACTTCATCAACAT
CGCCGAGAACAAGCTGCTGGAGGCCGAGGACAAGGGCGACCTGTACCTGTTCGAGATCACCA
ACAAGGACTTCAGCGGCAAGAAGAGCGGCAAGGACAACATCCACACCATCTACTGGAAGTAC
CTGTTCAGCGAGAGCAACTGCAAGAGCCCCATCATCGGCCTGAACGGCGGCGCCGAGATCTT
CTTCCGCGAGGGCCAGAAGGACAAGCTGCACACCAAGCTGGACAAGAAGGGCAAGAAGGTGT
TCGACGCCAAGCGCTACAGCGAGGACAAGCTGTTCTTCCACGTGAGCATCACCATCAACTAC
GGCAAGCCCAAGAACATCAAGTTCCGCGACATCATCAACCAGCTGATCACCAGCATGAACGT
GAACATCATCGGCATCGACCGCGGCGAGAAGCACCTGCTGTACTACAGCGTGATCGACAGCA
ACGGCATCATCCTGAAGCAGGGCAGCCTGAACAAGATCCGCGTGGGCGACAAGGAGGTGGAC
TTCAACAAGAAGCTGACCGAGCGCGCCAACGAGATGAAGAAGGCCCGCCAGAGCTGGGAGCA
GATCGGCAACATCAAGAACTTCAAGGAGGGCTACCTGAGCCAGGCCATCcacgagATCTACC
AGCTGATGATCAAGTACAACGCCATCATCGTGCTGGAGGACCTGAACACCGAGTTCAAGGCC
AAGCGCCTGAGCAAGGTGGAGAAGAGCGTGTACAAGAAGTTCGAGCTGAAGCTGGCCCGCAA
GCTGAACCACCTGATCCTGAAGGACCGCAACACCAACGAGATCGGCGGCGTGCTGAAGGCCT
ACCAGCTGACCCCCACCATCGGCGGCGGCGACGTGAGCAAGTTCGAGAAGGCCAAGCAGTGG
GGCATGATGTTCTACGTGCGCGCCAACTACACCAGCACCACCGACCCCGTGACCGGCTGGCG
CAAGCACCTGTACATCAGCAACTTCAGCAACAACAGCGTGATCAAGAGCTTCTTCGACCCCA
CCAACCGCGACACCGGCATCGAGATCTTCTACAGCGGCAAGTACCGCAGCTGGGGCTTCCGC
TACGTGCAGAAGGAGACCGGCAAGAAGTGGGAGCTGTTCGCCACCAAGGAGCTGGAGCGCTT
CAAGTACAACCAGACCACCAAGCTGTGCGAGAAGATCAACCTGTACGACAAGTTCGAGGAGC
TGTTCAAGGGCATCGACAAGAGCGCCGACATCTACAGCCAGCTGTGCAACGTGCTGGACTTC
CGCTGGAAGAGCCTGGTGTACCTGTGGAACCTGCTGAACCAGATCCGCAACGTGGACAAGAA
CGCCGAGGGCAACAAGAACGACTTCATCCAGAGCCCCGTGTACCCCTTCTTCGACAGCCGCA
AGACCGACGGCAAGACCGAGCCCATCAACGGCGACGCCAACGGCGCCCTGAACATCGCCCGC
AAGGGCCTGATGCTGGTGGAGCGCATCAAGAACAACCCCGAGAAGTACGAGCAGCTGATCCG
CGACACCGAGTGGGACGCCTGGATCCAGAACTTCAACAAGGTGAAC

C6Cas12a protein sequence

MKNVFGGFTNLYSLTKTLRFELKPTSKTQKLMKRNNVIQTDEEIDKLYHDEMKPILDEIHRR
FINDALAQKIFISASLDNFLKVVKNYKVESAKKNIKQNQVKLLQKEITIKTLGLRREVVSGF

ITVSKKWKDKYVGLGIKLKGDGYKVLTEQAVLDILKIEFPNKAKYIDKFRGFWTYFSGFNEN
RKNYYSEEDKATSIANRIVNENLSRYIDNIIAFEEILQKIPNLKKFKQDLDITSYNYYLNQA
GIDKYNKIIGGYIVDKDKKIQGINEKVNLYTQQTKKKLPKLKFLFKQIGSERKGFGIFEIKE
GKEWEQLGDLFKLQRTKINSNGREKGLFDSLRTMYREFFDEIKRDSNSQARYSLDKIYFNKA
SVNTISNSWFTNWNKFAELLNIKEDKKNGEKKIPEQISIEDIKDSLSIIPKENLEELFKLTN
REKHDRTRFFGSNAWVTFLNIWQNEIEESFNKLEEKEKDFKKNAAIKFQKNNLVQKNYIKEV
CDRMLAIERMAKYHLPKDSNLSREEDFYWIIDNLSEQREIYKYYNAFRNYISKKPYNKSKMK
LNFENGNLLGGWSDGQERNKAGVILRNGNKYYLGVLINRGIFRTDKINNEIYRTGSSKWERL
ILSNLKFQTLAGKGFLGKHGVSYGNMNPEKSVPSLQKFIRENYLKKYPQLTEVSNTKFLSKK
DFDAAIKEALKECFTMNFINIAENKLLEAEDKGDLYLFEITNKDFSGKKSGKDNIHTIYWKY
LFSESNCKSPIIGLNGGAEIFFREGQKDKLHTKLDKKGKKVFDAKRYSEDKLFFHVSITINY
GKPKNIKFRDIINQLITSMNVNIIGIDRGEKHLLYYSVIDSNGIILKQGSLNKIRVGDKEVD
FNKKLTERANEMKKARQSWEQIGNIKNFKEGYLSQAIHEIYQLMIKYNAIIVLEDLNTEFKA
KRLSKVEKSVYKKFELKLARKLNHLILKDRNTNEIGGVLKAYQLTPTIGGGDVSKFEKAKQW
GMMFYVRANYTSTTDPVTGWRKHLYISNFSNNSVIKSFFDPTNRDTGIEIFYSGKYRSWGFR
YVQKETGKKWELFATKELERFKYNQTTKLCEKINLYDKFEELFKGIDKSADIYSQLCNVLDF
RWKSLVYLWNLLNQIRNVDKNAEGNKNDFIQSPVYPFFDSRKTDGKTEPINGDANGALNIAR
KGLMLVERIKNNPEKYEQLIRDTEWDAWIQNFNKVN

Humanized Cas12a coding sequence derived from *Helcococcus kunzii* ATCC 51366 (GeneBank ID: JH601088.1/AGEI01000022.1)

ATGTTCGAGAAGCTGAGCAACATCGTGAGCATCAGCAAGACCATCCGCTTCAAGCTGATCCC
CGTGGGCAAGACCCTGGAGAACATCGAGAAGCTGGGCAAGCTGGAGAAGGACTTCGAGCGCA
GCGACTTCTACCCCATCCTGAAGAACATCAGCGACGACTACTACCGCCAGTACATCAAGGAG
AAGCTGAGCGACCTGAACCTGGACTGGCAGAAGCTGTACGACGCCCACGAGCTGCTGGACAG
CAGCAAGAAGGAGAGCCAGAAGAACCTGGAGATGATCCAGGCCCAGTACCGCAAGGTGCTGT
TCAACATCCTGAGCGGCGAGCTGGACAAGAGCGGCGAGAAGAACAGCAAGGACCTGATCAAG
AACAACAAGGCCCTGTACGGCAAGCTGTTCAAGAAGCAGTTCATCCTGGAGGTGCTGCCCGA
CTTCGTGAACAACAACGACAGCTACAGCGAGGAGGACCTGGAGGGCCTGAACCTGTACAGCA
AGTTCACCACCCGCCTGAAGAACTTCTGGGAGACCCGCAAGAACGTGTTCACCGACAAGGAC
ATCGTGACCGCCATCCCCTTCCGCGCCGTGAACGAGAACTTCGGCTTCTACTACGACAACAT
CAAGATCTTCAACAAGAACATCGAGTACCTGGAGAACAAGATCCCCAACCTGGAGAACGAGC
TGAAGGAGGCCGACATCCTGGACGACAACCGCAGCGTGAAGGACTACTTCACCCCCAACGGC
TTCAACTACGTGATCACCCAGGACGGCATCGACGTGTACCAGGCCATCCGCGGCGGCTTCAC
CAAGGAGAACGGCGAGAAGGTGCAGGGCATCAACGAGATCCTGAACCTGACCCAGCAGCAGC
TGCGCCGCAAGCCCGAGACCAAGAACGTGAAGCTGGGCGTGCTGACCAAGCTGCGCAAGCAG
ATCCTGGAGTACAGCGAGAGCACCAGCTTCCTGATCGACCAGATCGAGGACGACAACGACCT
GGTGGACCGCATCAACAAGTTCAACGTGAGCTTCTTCGAGAGCACCGAGGTGAGCCCCAGCC
TGTTCGAGCAGATCGAGCGCCTGTACAACGCCCTGAAGAGCATCAAGAAGGAGGAGGTGTAC
ATCGACGCCCGCAACACCCAGAAGTTCAGCCAGATGCTGTTCGGCCAGTGGGACGTGATCCG
CCGCGGCTACACCGTGAAGATCACCGAGGGCAGCAAGGAGGAGAAGAAGAAGTACAAGGAGT
ACCTGGAGCTGGACGAGACCAGCAAGGCCAAGCGCTACCTGAACATCCGCGAGATCGAGGAG
CTGGTGAACCTGGTGGAGGGCTTCGAGGAGGTGGACGTGTTCAGCGTGCTGCTGGAGAAGTT
CAAGATGAACAACATCGAGCGCAGCGAGTTCGAGGCCCCCATCTACGGCAGCCCCATCAAGC

TGGAGGCCATCAAGGAGTACCTGGAGAAGCACCTGGAGGAGTACCACAAGTGGAAGCTGCTG
CTGATCGGCAACGACGACCTGGACACCGACGAGACCTTCTACCCCCTGCTGAACGAGGTGAT
CAGCGACTACTACATCATCCCCCTGTACAACCTGACCCGCAACTACCTGACCCGCAAGCACA
GCGACAAGGACAAGATCAAGGTGAACTTCGACTTCCCCACCCTGGCCGACGGCTGGAGCGAG
AGCAAGATCAGCGACAACCGCAGCATCATCCTGCGCAAGGGCGGCTACTACTACCTGGGCAT
CCTGATCGACAACAAGCTGCTGATCAACAAGAAGAACAAGAGCAAGAAGATCTACGAGATCC
TGATCTACAACCAGATCCCCGAGTTCAGCAAGAGCATCCCCAACTACCCCTTCACCAAGAAG
GTGAAGGAGCACTTCAAGAACAACGTGAGCGACTTCCAGCTGATCGACGGCTACGTGAGCCC
CCTGATCATCACCAAGGAGATCTACGACATCAAGAAGGAGAAGAAGTACAAGAAGGACTTCT
ACAAGGACAACAACACCAACAAGAACTACCTGTACACCATCTACAAGTGGATCGAGTTCTGC
AAGCAGTTCCTGTACAAGTACAAgggcccCAACAAGGAGAGCTACAAGGAGATGTACGACTT
CAGCACCCTGAAGGACACCAGCCTGTACGTGAACCTGAACGACTTCTACGCCGACGTGAACA
GCTGCGCCTACCGCGTGCTGTTCAACAAGATCGACGAGAACACCATCGACAACGCCGTGGAG
GACGGCAAGCTGCTGCTGTTCCAGATCTACAACAAGGACTTCAGCCCCGAGAGCAAGGGCAA
GAAGAACCTGCACACCCTGTACTGGCTGAGCATGTTCAGCGAGGAGAACCTGCGCACCCGCA
AGCTGAAGCTGAACGGCCAGGCCGAGATCTTCTACCGCAAGAAGCTGGAGAAGAAGCCCATC
ATCCACAAGGAGGGCAGCATCCTGCTGAACAAGATCGACAAGGAGGGCAACACCATCCCCGA
GAACATCTACCACGAGTGCTACCGCTACCTGAACAAGAAGATCGGCCGCGAGGACCTGAGCG
ACGAGGCCATCGCCCTGTTCAACAAGGACGTGCTGAAGTACAAGGAGGCCCGCTTCGACATC
ATCAAGGACCGCCGCTACAGCGAGAGCCAGTTCTTCTTCCACGTGCCCATCACCTTCAACTG
GGACATCAAGACCAACAAGAACGTGAACCAGATCGTGCAGGGCATGATCAAGGACGGCGAGA
TCAAGCACATCATCGGCATCGACCGCGGCGAGCGCCACCTGCTGTACTACAGCGTGATCGAC
CTGGAGGGCAACATCGTGGAGCAGGGCAGCCTGAACACCCTGGAGCAGAACCGCTTCGACAA
CAGCACCGTGAAGGTGGACTACCAGAACAAGCTGCGCACCCGCGAGGAGGACCGCGACCGCG
CCCGCAAGAACTGGACCAACATCAACAAGATCAAGGAGCTGAAGGACGGCTACCTGAGCCAC
GTGGTGCACAAGCTGAGCCGCCTGATCATCAAGTACGAGGCCATCGTGATCATGGAGAACCT
GAACCAGGGCTTCAAGCGCGGCCGCTTCAAGGTGGAGCGCCAGGTGTACCAGAAGTTCGAGC
TGGCCCTGATGAACAAGCTGAGCGCCCTGAGCTTCAAGGAGAAGTACGACGAGCGCAAGAAC
CTGGAGCCCAGCGGCATCCTGAACCCCATCCAGGCCTGCTACCCCGTGGACGCCTACCAGGA
GCTGCAGGGCCAGAACGGCATCGTGTTCTACCTGCCCGCCGCCTACACCAGCGTGATCGACC
CCGTGACCGGCTTCACCAACCTGTTCCGCCTGAAGAGCATCAACAGCAGCAAGTACGAGGAG
TTCATCAAGAAGTTCAAGAACATCTACTTCGACAACGAGGAGGAGGACTTCAAGTTCATCTT
CAACTACAAGGACTTCGCCAAGGCCAACCTGGTGATCCTGAACAACATCAAGAGCAAGGACT
GGAAGATCAGCACCCGCGGCGAGCGCATCAGCTACAACAGCAAGAAGAAGGAGTACTTCTAC
GTGCAGCCCACCGAGTTCCTGATCAACAAGCTGAAGGAGCTGAACATCGACTACGAGAACAT
CGACATCATCCCCCTGATCGACAACCTGGAGGAGAAGGCCAAGCGCAAGATCCTGAAGGCCC
TGTTCGACACCTTCAAGTACAGCGTGCAGCTGCGCAACTACGACTTCGAGAACGACTACATC
ATCAGCCCCACCGCCGACGACAACGGCAACTACTACAACAGCAACGAGATCGACATCGACAA
GACCAACCTGCCCAACAACGGCGACGCCAACGGCGCCTTCAACATCGCCCGCAAGGGCCTGC
TGCTGAAGGACCGCATCGTGAACAGCAACGAGAGCAAGGTGGACCTGAAGATCAAGAACGAG
GACTGGATCAACTTCATCATCAGC

HkCas12a protein sequence

MFEKLSNIVSISKTIRFKLIPVGKTLENIEKLGKLEKDFERSDFYPILKNISDDYYRQYIKE
KLSDLNLDWQKLYDAHELLDSSKKESQKNLEMIQAQYRKVLFNILSGELDKSGEKNSKDLIK
NNKALYGKLFKKQFILEVLPDFVNNNDSYSEEDLEGLNLYSKFTTRLKNFWETRKNVFTDKD
IVTAIPFRAVNENFGFYYDNIKIFNKNIEYLENKIPNLENELKEADILDDNRSVKDYFTPNG
FNYVITQDGIDVYQAIRGGFTKENGEKVQGINEILNLTQQQLRRKPETKNVKLGVLTKLRKQ
ILEYSESTSFLIDQIEDDNDLVDRINKFNVSFFESTEVSPSLFEQIERLYNALKSIKKEEVY
IDARNTQKFSQMLFGQWDVIRRGYTVKITEGSKEEKKKYKEYLELDETSKAKRYLNIREIEE
LVNLVEGFEEVDVFSVLLEKFKMNNIERSEFEAPIYGSPIKLEAIKEYLEKHLEEYHKWKLL
LIGNDDLDTDETFYPLLNEVISDYYIIPLYNLTRNYLTRKHSDKDKIKVNFDFPTLADGWSE
SKISDNRSIILRKGGYYYLGILIDNKLLINKKNKSKKIYEILIYNQIPEFSKSIPNYPFTKK
VKEHFKNNVSDFQLIDGYVSPLIITKEIYDIKKEKKYKKDFYKDNNTNKNYLYTIYKWIEFC
KQFLYKYKGPNKESYKEMYDFSTLKDTSLYVNLNDFYADVNSCAYRVLFNKIDENTIDNAVE
DGKLLLFQIYNKDFSPESKGKKNLHTLYWLSMFSEENLRTRKLKLNGQAEIFYRKKLEKKPI
IHKEGSILLNKIDKEGNTIPENIYHECYRYLNKKIGREDLSDEAIALFNKDVLKYKEARFDI
IKDRRYSESQFFFHVPITFNWDIKTNKNVNQIVQGMIKDGEIKHIIGIDRGERHLLYYSVID
LEGNIVEQGSLNTLEQNRFDNSTVKVDYQNKLRTREEDRDRARKNWTNINKIKELKDGYLSH
VVHKLSRLIIKYEAIVIMENLNQGFKRGRFKVERQVYQKFELALMNKLSALSFKEKYDERKN
LEPSGILNPIQACYPVDAYQELQGQNGIVFYLPAAYTSVIDPVTGFTNLFRLKSINSSKYEE
FIKKFKNIYFDNEEEDFKFIFNYKDFAKANLVILNNIKSKDWKISTRGERISYNSKKKEYFY
VQPTEFLINKLKELNIDYENIDIIPLIDNLEEKAKRKILKALFDTFKYSVQLRNYDFENDYI
ISPTADDNGNYYNSNEIDIDKTNLPNNGDANGAFNIARKGLLLKDRIVNSNESKVDLKIKNE
DWINFIIS

Humanized Cas12a coding sequence derived from *Lachnospira pectinoschiza* strain 2789STDY5834836 (GeneBank ID: CZAK01000004.)

ATGATCATGAACAACGTGACCGGCGACTTCAGCGAGTTCGTGGCCATCAGCAAGGTGCAGAA
GACCCTGCGCAACGAGCTGCGCCCCACCCCCCTGACCATGAAGCACATCAAGCAGAAGGGCA
TCATCACCGAGGACGAGTACAAGACCCAGCAGAGCCTGGAGCTGAAGCGCATCGCCGACGGC
TACTACCGCGACTACATCACCCACAAGCTGAACGACACCAACAACCTGGACTTCCGCAACCT
GTTCGAGGCCATCGAGGAGAAGTACAAGAAGAACGACAAGGACAACCGCGACAAGCTGGACC
TGGTGGAGAAGAGCAAGCGCGGCGAGATCGCCAAGCTGCTGAGCGCCGACGACAACTTCAAG
AGCATGTTCGAGGCCAAGCTGATCACCCAGCTGCTGCCCGTGTACGTGGAGCAGAACTACAT
CGGCGAGGACAAGGAGAAGGCCCTGGAGACCATCGCCCTGTTCAAGGGCTTCACCACCTACT
TCACCGACTACTTCAACATCCGCAAGAACATGTTCAAGGAGAACGGCGGCGCCAGCAGCATC
TGCTACCGCATCGTGAACGTGAACGCCAGCATCTTCTACGACAACCTGAAGACCTTCATGTG
CATCAAGGAGAAGGCCGAGACCGAGATCGCCCTGATCGAGGAGGAGCTGACCGAGCTGCTGG
ACAGCTGGCGCCTGGAGCACATCTTCAGCGAGGACTACTACAACGAGCTGCTGGCCCAGAAG
GGCATCGACTACTACAACCAGATCTGCGGCGACGTGAACAAGCACATGAACCTGTACTGCCA
GCAGAACAAGCTGAAGGCCAACGTGTTCAAGATGACCAAGCTGCAGAAGCAGATCATGGGCA
TCAGCGAGAAGGCCTTCGAGATCCCCCCCATGTACCAGAACGACGAGGAGGTGTACGCCGCC
TTCAACGGCTTCATCAGCCGCCTGGAGGAGGTGAAGCTGATCGACCGCCTGGGCAACGTGCT
GCAGAACAGCAACATCTACGACACCGCCAAGATCTACATCAACGCCCGCTGCTACACCAACG
TGAGCAGCTACGTGTACGGCGGCTGGGGCGTGATCGAGAGCGCCATCGAGCGCTACTGGTAC
AACACCATCGCCGGCAAGGGCCAGAGCAAGGCCAAGAAGATCGAGAAGGCCAAGAAGGACAA

CAAGTTCATGAGCGTGAAGGAGCTGGACAGCATCGTGAGCGACTACGAGCCCGACTACTTCA
ACGCCAGCAACATGGACGACGACAACAGCGGCCGCGCCTTCAGCGGCCACGGCGTGCTGGGC
TACTTCAACAAGATGAGCAAGCTGCTGGCCAACATGAGCCTGCACACCATCACCTACGACAG
CGGCGACAGCCTGATCGAGAACAAGGAGACCGCCCTGAACATCAAGAAGGACCTGGACGACA
TCATGAGCATCTACCACTGGCTGCAGACCTTCATCATCGACGAGGTGGTGGAGAAGGACAAC
GCCTTCTACGCCGAGCTGGAGGACATCTACTACGAGCTGGAGAACGTGGTGACCCTGTACGA
CCGCATCCGCAACTACGTGACCCGCAAGCCCTACAGCACCCAGAAGTTCaagcttAACTTCG
CCAGCCCCACCCTGGCCAGCGGCTGGAGCCGCAGCAAGGAGTTCGACAACAACGCCATCATC
CTGCTGCGCAACAACAAGTACTACATCGCCATCTTCAACGTGAACAACAAGCCCGACAAGCA
GATCATCAAGGGCAGCGAGGAGCAGCGCCTGAGCACCGACTACAAGAAGATGGTGTACAACC
TGCTGCCCGGCCCCAACAAGATGCTGCCCTGGGTGTTCATCAAGAGCAACACCGGCAAGCGC
GACTACAACCCCAGCAGCTACATCCTGGAGGGCTACGAGAAGAACCGCCACATCAAGAGCAG
CGGCAACTTCGACATCAACTACTGCCACGACCTGATCGACTACTACAAGGCCTGCATCAACA
AGCACCCCGAGTGGAAGAACTACGGCTTCAAGTTCAAGGAGACCACCCAGTACAACGACATC
GGCCAGTTCTACAAGGACGTGGAGAAGCAGGGCTACAGCATCAGCTGGGCCTACATCAGCGA
GGCCGACATCAACCGCCTGGACGAGGAGGGCAAGATCTACCTGTTCGAGATCTACAACAAGG
ACCTGAGCAGCCACAGCACCGGCAAGGACAACCTGCACACCATGTACCTGAAGAACATCTTC
AGCGAGGACAACCTGAAGAACATCTGCATCGAGCTGAACGGCAACGCCGAGCTGTTCTACCG
CAAGAGCAGCATGAAGCGCAACATCACCCACAAGAAGGACACCGTGCTGGTGAACAAGACCT
ACATCAACGAGGCCGGCGTGCGCGTGAGCCTGACCGACGAGGACTACATCAAGGTGTACAAC
TACTACAACAACGACTACGTGATCGACGTGGAGAAGGACAAGAAGCTGGTGGAGATCCTGGA
GCGCATCGGCCACCGCAAGAACCCCATCGACATCATCAAGGACAAGCGCTACACCGAGGACA
AGTACTTCCTGCACTTCCCCATCACCATCAACTACGGCGTGGACGACGAGAACATCAACGCC
AAGATGATCGAGTACATCGCCAAGCACAACAACATGAACGTGATCGGCATCGACCGCGGCGA
GCGCAACCTGATCTACATCAGCGTGATCAACAACAAGGGCAACATCATCGAGCAGAAGAGCT
TCAACCTGGTGAACAACTACGACTACAAGAACAAGCTGAAGAACATGGAGAAGACCCGCGAC
AACGCCCGCAAGAACTGGCAGGAGATCGGCAAGATCAAGGACGTGAAGAACGGCTACCTGAG
CGGCGTGATCAGCAAGATCGCCCGCATGGTGGTGGACTACAACGCCATCATCGTGATGGAGG
ACCTGAACCGCGGCTTCAAGCGCGGCCGCTTCAAGGTGGAGCGCCAGGTGTACCAGAAGTTC
GAGAACATGCTGATCAGCAAGCTGAACTACCTGGTGTTCAAGGAGAAGAAGGCCGACGAGAA
CGGCGGCATCCTGAAGGGCTACCAGCTGACCTACCTGCCCAAGAGCGCCCTGCAGATCGGCA
AGCAGTGCGGCTGCATCTTCTACGTGCCCGCCGCCTACACCAGCAAGATCGACCCCGCCACC
GGCTTCATCAACATCTTCGACTTCAAGAAGTACAGCGGCAGCGCCATCAACGCCAAGGTGAA
GGACAAGAAGGAGTTCCTGATGAGCATGAACAGCATCCGCTACGTGAACGAGGGCAGCGCCG
AGTACGAGAAGATCGGCCACCGCCAGCTGTTCGCCTTCAGCTTCGACTACAACAACTTCAAG
ACCTACAACGTGAGCATCCCCGTGAACGAGTGGACCACCTACACCTACGGCGAGCGCATCAA
GAAGCTGTACAAGGACGGCCGCTGGAGCGGCAGCGAGGTGCTGAACCTGACCGAGGACCTGA
TCGAGCTGATGGAGCAGTACGGCATCGAGTACAAGGACGGCCACGACATCCGCGAGGACATC
AGCCACATGGACGAGATGCGCAACGCCGACTTCATCTGCAACCTGTTCGAGAAGTTCAAGTA
CACCGTGCAGCTGCGCAACAGCAAGAGCGAGGCCGAGGGCGACGACTACGACCGCCTGGTGA
GCCCCGTGCTGAACAGCCACAACGGCTTCTTCGACAGCAGCGACTACAAGGAGAACGAGAAG
AGCGACGACATCATCGACGACAAGCAGATCATGCCCAAGGACGCCGACGCCAACGGCGCCTA
CTGCATCGCCCTGAAGGGCCTGTACGAGATCAACAAGATCAAGGAGAACTGGAGCGACGACA

AGAAGCTGAAGGAGAGCGAGCTGTACATCGGCGTGACCGAGTGGCTGGACTACATCCAGAAC
CGCCGCTTCGAG

LpCas12a protein sequence

MIMNNVTGDFSEFVAISKVQKTLRNELRPTPLTMKHIKQKGIITEDEYKTQQSLELKRIADG
YYRDYITHKLNDTNNLDFRNLFEAIEEKYKKNDKDNRDKLDLVEKSKRGEIAKLLSADDNFK
SMFEAKLITQLLPVYVEQNYIGEDKEKALETIALFKGFTTYFTDYFNIRKNMFKENGGASSI
CYRIVNVNASIFYDNLKTFMCIKEKAETEIALIEEELTELLDSWRLEHIFSEDYYNELLAQK
GIDYYNQICGDVNKHMNLYCQQNKLKANVFKMTKLQKQIMGISEKAFEIPPMYQNDEEVYAA
FNGFISRLEEVKLIDRLGNVLQNSNIYDTAKIYINARCYTNVSSYVYGGWGVIESAIERYWY
NTIAGKGQSKAKKIEKAKKDNKFMSVKELDSIVSDYEPDYFNASNMDDDNSGRAFSGHGVLG
YFNKMSKLLANMSLHTITYDSGDSLIENKETALNIKKDLDDIMSIYHWLQTFIIDEVVEKDN
AFYAELEDIYYELENVVTLYDRIRNYVTRKPYSTQKFKLNFASPTLASGWSRSKEFDNNAII
LLRNNKYYIAIFNVNNKPDKQIIKGSEEQRLSTDYKKMVYNLLPGPNKMLPWVFIKSNTGKR
DYNPSSYILEGYEKNRHIKSSGNFDINYCHDLIDYYKACINKHPEWKNYGFKFKETTQYNDI
GQFYKDVEKQGYSISWAYISEADINRLDEEGKIYLFEIYNKDLSSHSTGKDNLHTMYLKNIF
SEDNLKNICIELNGNAELFYRKSSMKRNITHKKDTVLVNKTYINEAGVRVSLTDEDYIKVYN
YYNNDYVIDVEKDKKLVEILERIGHRKNPIDIIKDKRYTEDKYFLHFPITINYGVDDENINA
KMIEYIAKHNNMNVIGIDRGERNLIYISVINNKGNIIEQKSFNLVNNYDYKNKLKNMEKTRD
NARKNWQEIGKIKDVKNGYLSGVISKIARMVVDYNAIIVMEDLNRGFKRGRFKVERQVYQKF
ENMLISKLNYLVFKEKKADENGGILKGYQLTYLPKSALQIGKQCGCIFYVPAAYTSKIDPAT
GFINIFDFKKYSGSAINAKVKDKKEFLMSMNSIRYVNEGSAEYEKIGHRQLFAFSFDYNNFK
TYNVSIPVNEWTTYTYGERIKKLYKDGRWSGSEVLNLTEDLIELMEQYGIEYKDGHDIREDI
SHMDEMRNADFICNLFEKFKYTVQLRNSKSEAEGDDYDRLVSPVLNSHNGFFDSSDYKENEK
SDDIIDDKQIMPKDADANGAYCIALKGLYEINKIKENWSDDKKLKESELYIGVTEWLDYIQN
RRFE

Humanized Cas12a coding sequence derived from *Oribacterium sp*. NK2B42 (GeneBank ID: NZ_KE384190.1)

ATGGAGACCGAGATCCTGAAGTACGACTTCTTCGAGCGCGAGGGCAAGTACATGTACTACGA
CGGCCTGACCAAGCAGTACGCCCTGAGCAAGACCATCCGCAACGAGCTGGTGCCCATCGGCA
AGACCCTGGACAACATCAAGAAGAACCGCATCCTGGAGGCCGACATCAAGCGCAAGAGCGAC
TACGAGCACGTGAAGAAGCTGATGGACATGTACCACAAGAAGATCATCAACGAGGCCCTGGA
CAACTTCAAGCTGAGCGTGCTGGAGGACGCCGCCGACATCTACTTCAACAAGCAGAACGACG
AGCGCGACATCGACGCCTTCCTGAAGATCCAGGACAAGCTGCGCAAGGAGATCGTGGAGCAG
CTGAAGGGCCACACCGACTACAGCAAGGTGGGCAACAAGGACTTCCTGGGCCTGCTGAAGGC
CGCCAGCACCGAGGAGGACCGCATCCTGATCGAGAGCTTCGACAACTTCTACACCTACTTCA
CCAGCTACAACAAGGTGCGCAGCAACCTGTACAGCGCCGAGGACAAGAGCAGCACCGTGGCC
TACCGCCTGATCAACGAGAACCTGCCCAAGTTCTTCGACAACATCAAGGCCTACCGCACCGT
GCGCAACGCCGGCGTGATCAGCGGCGACATGAGCATCGTGGAGCAGGACGAGCTGTTCGAGG
TGGACACCTTCAACCACACCCTGACCCAGTACGGCATCGACACCTACAACCACATGATCGGC
CAGCTGAACAGCGCCATCAACCTGTACAACCAGAAGATGCACGGCGCCGGCAGCTTCAAGAA
GCTGCCCAAGATGAAGGAGCTGTACAAGCAGCTGCTGACCGAGCGCGAGGAGGAGTTCATCG
AGGAGTACACCGACGACGAGGTGCTGATCACCAGCGTGCACAACTACGTGAGCTACCTGATC

GACTACCTGAACAGCGACAAGGTGGAGAGCTTCTTCGACACCCTGCGCAAGAGCGACGGCAA
GGAGGTGTTCATCAAGAACGACGTGAGCAAGACCACCATGAGCAACATCCTGTTCGACAACT
GGAGCACCATCGACGACCTGATCAACCACGAGTACGACAGCGCCCCCGAGAACGTGAAGAAG
ACCAAGGACGACAagtactTCGAGAAGCGCCAGAAGGACCTGAAGAAGAACAAGAGCTACAG
CCTGAGCAAGATCGCCGCCCTGTGCCGCGACACCACCATCCTGGAGAAGTACATCCGCCGCC
TGGTGGACGACATCGAGAAGATCTACACCAGCAACAACGTGTTCAGCGACATCGTGCTGAGC
AAGCACGACCGCAGCAAGAAGCTGAGCAAGAACACCAACGCCGTGCAGGCCATCAAGAACAT
GCTGGACAGCATCAAGGACTTCGAGCACGACGTGATGCTGATCAACGGCAGCGGCCAGGAGA
TCAAGAAGAACCTGAACGTGTACAGCGAGCAGGAGGCCCTGGCCGGCATCCTGCGCCAGGTG
GACCACATCTACAACCTGACCCGCAACTACCTGACCAAGAAGCCCTTCAGCACCGAGAAGAT
CAAGCTGAACTTCAACCGCCCCACCTTCCTGGACGGCTGGGACAAGAACAAGGAGGAGGCCA
ACCTGGGCATCCTGCTGATCAAGGACAACCGCTACTACCTGGGCATCATGAACACCAGCAGC
AACAAGGCCTTCGTGAACCCCCCCAAGGCCATCAGCAACGACATCTACAAGAAGGTGGACTA
CAAGCTGCTGCCCGGCCCCAACAAGATGCTGCCCAAGGTGTTCTTCGCCACCAAGAACATCG
CCTACTACGCCCCCAGCGAGGAGCTGCTGAGCAAGTACCGCAAGGGCACCCACAAGAAGGGC
GACAGCTTCAGCATCGACGACTGCCGCAACCTGATCGACTTCTTCAAGAGCAGCATCAACAA
GAACACCGACTGGAGCACCTTCGGCTTCAACTTCAGCGACACCAACAGCTACAACGACATCA
GCGACTTCTACCGCGAGGTGGAGAAGCAGGGCTACAAGCTGAGCTTCACCGACATCGACGCC
TGCTACATCAAGGACCTGGTGGACAACAACGAGCTGTACCTGTTCCAGATCTACAACAAGGA
CTTCAGCCCCTACAGCAAGGGCAAGCTGAACCTGCACACCCTGTACTTCAAGATGCTGTTCG
ACCAGCGCAACCTGGACAACGTGGTGTACAAGCTGAACGGCGAGGCCGAGGTGTTCTACCGC
CCCGCCAGCATCGAGAGCGACGAGCAGATCATCCACAAGAGCGGCCAGAACATCAAGAACAA
GAACCAGAAGCGCAGCAACTGCAAGAAGACCAGCACCTTCGACTACGACATCGTGAAGGACC
GCCGCTACTGCAAGGACAAGTTCATGCTGCACCTGCCCATCACCGTGAACTTCGGCACCAAC
GAGAGCGGCAAGTTCAACGAGCTGGTGAACAACGCCATCCGCGCCGACAAGGACGTGAACGT
GATCGGCATCGAccgcggCGAGCGCAACCTGCTGTACGTGGTGGTGGTGGACCCCTGCGGCA
AGATCATCGAGCAGATCAGCCTGAACACCATCGTGGACAAGGAGTACGACATCGAGACCGAC
TACCACCAGCTGCTGGACGAGAAGGAGGGCAGCCGCGACAAGGCCCGCAAGGACTGGAACAC
CATCGAGAACATCAAGGAGCTGAAGGAGGGCTACCTGAGCCAGGTGGTGAACATCATCGCCA
AGCTGGTGCTGAAGTACGACGCCATCATCTGCCTGGAGGACCTGAACTTCGGCTTCAAGCGC
GGCCGCCAGAAGGTGGAGAAGCAGGTGTACCAGAAGTTCGAGAAGATGCTGATCGACAAGAT
GAACTACCTGGTGCTGGACAAGAGCCGCAAGCAGGAGAGCCCCCAGAAGCCCGGCGGCGCCC
TGAACGCCCTGCAGCTGACCAGCGCCTTCAAGAGCTTCAAGGAGCTGGGCAAGCAGACCGGC
ATCATCTACTACGTGCCCGCCTACCTGACCAGCAAGATCGACCCCACCACCGGCTTCGCCAA
CCTGTTCTACATCAAGTACGAGAGCGTGGACAAGGCCCGCGACTTCTTCAGCAAGTTCGACT
TCATCCGCTACAACCAGATGGACAACTACTTCGAGTTCGGCTTCGACTACAAGAGCTTCACC
GAGCGCGCCAGCGGCTGCAAGAGCAAGTGGATCGCCTGCACCAACGGCGAGCGCATCGTGAA
GTACCGCAACAGCGACAAGAACAACAGCTTCGACGACAAGACCGTGATCCTGACCGACGAGT
ACCGCAGCCTGTTCGACAAGTACCTGCAGAACTACATCGACGAGGACGACCTGAAGGACCAG
ATCCTGCAGATCGACAGCGCCGACTTCTACAAGAACCTGATCAAGCTGTTCCAGCTGACCCT
GCAGATGCGCAACAGCAGCAGCGACGGCAAGCGCGACTACATCATCAGCCCCGTGAAGAACT
ACCGCGAGGAGTTCTTCTGCAGCGAGTTCAGCGACGACACCTTCCCCCGCGACGCCGACGCC
AACGGCGCCTACAACATCGCCCGCAAGGGCCTGTGGGTGATCAAGCAGATCCGCGAGACCAA

GAGCGGCACCAAGATCAACCTGGCCATGAGCAACAGCGAGTGGCTGGAGTACGCCCAGTGCA
ACCTGCTG

OsCas12a protein sequence

METEILKYDFFEREGKYMYYDGLTKQYALSKTIRNELVPIGKTLDNIKKNRILEADIKRKSD
YEHVKKLMDMYHKKIINEALDNFKLSVLEDAADIYFNKQNDERDIDAFLKIQDKLRKEIVEQ
LKGHTDYSKVGNKDFLGLLKAASTEEDRILIESFDNFYTYFTSYNKVRSNLYSAEDKSSTVA
YRLINENLPKFFDNIKAYRTVRNAGVISGDMSIVEQDELFEVDTFNHTLTQYGIDTYNHMIG
QLNSAINLYNQKMHGAGSFKKLPKMKELYKQLLTEREEEFIEEYTDDEVLITSVHNYVSYLI
DYLNSDKVESFFDTLRKSDGKEVFIKNDVSKTTMSNILFDNWSTIDDLINHEYDSAPENVKK
TKDDKYFEKRQKDLKKNKSYSLSKIAALCRDTTILEKYIRRLVDDIEKIYTSNNVFSDIVLS
KHDRSKKLSKNTNAVQAIKNMLDSIKDFEHDVMLINGSGQEIKKNLNVYSEQEALAGILRQV
DHIYNLTRNYLTKKPFSTEKIKLNFNRPTFLDGWDKNKEEANLGILLIKDNRYYLGIMNTSS
NKAFVNPPKAISNDIYKKVDYKLLPGPNKMLPKVFFATKNIAYYAPSEELLSKYRKGTHKKG
DSFSIDDCRNLIDFFKSSINKNTDWSTFGFNFSDTNSYNDISDFYREVEKQGYKLSFTDIDA
CYIKDLVDNNELYLFQIYNKDFSPYSKGKLNLHTLYFKMLFDQRNLDNVVYKLNGEAEVFYR
PASIESDEQIIHKSGQNIKNKNQKRSNCKKTSTFDYDIVKDRRYCKDKFMLHLPITVNFGTN
ESGKFNELVNNAIRADKDVNVIGIDRGERNLLYVVVVDPCGKIIEQISLNTIVDKEYDIETD
YHQLLDEKEGSRDKARKDWNTIENIKELKEGYLSQVVNIIAKLVLKYDAIICLEDLNFGFKR
GRQKVEKQVYQKFEKMLIDKMNYLVLDKSRKQESPQKPGGALNALQLTSAFKSFKELGKQTG
IIYYVPAYLTSKIDPTTGFANLFYIKYESVDKARDFFSKFDFIRYNQMDNYFEFGFDYKSFT
ERASGCKSKWIACTNGERIVKYRNSDKNNSFDDKTVILTDEYRSLFDKYLQNYIDEDDLKDQ
ILQIDSADFYKNLIKLFQLTLQMRNSSSDGKRDYIISPVKNYREEFFCSEFSDDTFPRDADA
NGAYNIARKGLWVIKQIRETKSGTKINLAMSNSEWLEYAQCNLL

Humanized Cas12a coding sequence derived from *Pseudobutyrivibrio ruminis* CF1b (GeneBank ID: NZ_KE384121.1)

ATGATCATCGGCCGCGACTTCAACATGTACTACCAGAACCTGACCAAGATGTACCCCATCAG
CAAGACCCTGCGCAACGAGCTGATCCCCGTGGGCAAGACCCTGGAGAACATCCGCAAGAACG
GCATCCTGGAGGCCGACATCCAGCGCAAGGCCGACTACGAGCACGTGAAGAAGCTGATGGAC
AACTACCACAAGCAGCTGATCAACGAGGCCCTGCAGGGCGTGCACCTGAGCGACCTGAGCGA
CGCCTACGACCTGTACTTCAACCTGAGCAAGGAGAAGAACAGCGTGGACGCCTTCAGCAAGT
GCCAGGACAAGCTGCGCAAGGAGATCGTGAGCCTGCTGAAGAACCACGAGAACTTCCCCAAG
ATCGGCAACAAGGAGATCATCAAGCTGCTGCAGAGCCTGTACGACAACGACACCGACTACAA
GGCCCTGGACAGCTTCAGCAACTTCTACACCTACTTCAGCAGCTACAACGAGGTGCGCAAGA
ACCTGTACAGCGACGAGGAGAAGAGCAGCACCGTGGCCTACCGCCTGATCAACGAGAACCTG
CCCAAGTTCCTGGACAACATCAAGGCCTACGCCATCGCCAAGAAGGCCGGCGTGCGCGCCGA
GGGCCTGAGCGAGGAGGACCAGGACTGCCTGTTCATCATCGAGACCTTCGAGCGCACCCTGA
CCCAGGACGGCATCGACAACTACAACGCCGCCATCGGCAAGCTGAACACCGCCATCAACCTG
TTCAACCAGCAGAACAAGAAGCAGGAGGGCTTCCGCAAGGTGCCCCAGATGAAGTGCCTGTA
CAAGCAGATCCTGAGCGACCGCGAGGAGGCCTTCATCGACGAGTTCAGCGACGACGAGGACC
TGATCACCAACATCGAGAGCTTCGCCGAGAACATGAACGTGTTCCTGAACAGCGAGATCATC
ACCGACTTCAAGATCGCCCTGGTGGAGAGCGACGGCAGCCTGGTGTACATCAAGAACGACGT
GAGCAAGACCAGCTTCAGCAACATCGTGTTCGGCAGCTGGAACGCCATCGACGAGAAGCTGA

GCGACGAGTACGACCTGGCCAACAGCAAGAAGAAGAAGGACGAGAagtactACGAGAAGCGC
CAGAAGGAGCTGAAGAAGAACAAGAGCTACGACCTGGAGACCATCATCGGCCTGTTCGACGA
CAACAGCGACGTGATCGGCAAGTACATCGAGAAGCTGGAGAGCGACATCACCGCCATCGCCG
AGGCCAAGAACGACTTCGACGAGATCGTGCTGCGCAAGCACGACAAGAACAAGAGCCTGCGC
AAGAACACCAACGCCGTGGAGGCCATCAAGAGCTACCTGGACACCGTGAAGGACTTCGAGCG
CGACATCAAGCTGATCAACGGCAGCGGCCAGGAGGTGGAGAAGAACCTGGTGGTGTACGCCG
AGCAGGAGAACATCCTGGCCGAGATCAAGAACGTGGACAGCCTGTACAACATGAGCCGCAAC
TACCTGACCCAGAAGCCCTTCAGCACCGAGAAGTTCAAGCTGAACTTCAACCGCGCCACCCT
GCTGAACGGCTGGGACAAGAACAAGGAGACCGACAACCTGGGCATCCTGTTCGAGAAGGACG
GCATGTACTACCTGGGCATCATGAACACCAAGGCCAACAAGATCTTCGTGAACATCCCCAAG
GCCACCAGCAACGACGTGTACCACAAGGTGAACTACAAGCTGCTGCCCGGCCCCAACAAGAT
GCTGCCCAAGGTGTTCTTCGCCCAGAGCAACCTGGACTACTACAAGCCCAGCGAGGAGCTGC
TGGCCAAGTACAAGGCCGGCACCCACAAGAAGGGCGACAACTTCAGCCTGGAGGACTGCCAC
GCCCTGATCGACTTCTTCAAGGCCAGCATCGAGAAGCACCCCGACTGGAGCAGCTTCGGCTT
CGAGTTCAGCGAGACCTGCACCTACGAGGACCTGAGCGGCTTCTACCGCGAGGTGGAGAAGC
AGGGCTACAAGATCACCTACACCGACGTGGACGCCGACTACATCACCAGCCTGGTGGAGCGC
GACGAGCTGTACCTGTTCCAGATCTACAACAAGGACTTCAGCCCCTACAGCAAGGGCAACCT
GAACCTGCACACCATCTACCTGCAGATGCTGTTCGACCAGCGCAACCTGAACAACGTGGTGT
ACAAGCTGAACGGCGAGGCCGAGGTGTTCTACCGCCCCGCCAGCATCAACGACGAGGAGGTG
ATCATCCACAAGGCCGGCGAGGAGATCAAGAACAAGAACAGCAAGCGCGCCGTGGACAAGCC
CACCAGCAAGTTCGGCTACGACATCATCAAGGACCGCCGCTACAGCAAGGACAAGTTCATGC
TGCACATCCCCGTGACCATGAACTTCGGCGTGGACGAGACCCGCCGCTTCAACGACGTGGTG
AACGACGCCCTGCGCAACGACGAGAAGGTGCGCGTGATCGGCATCGACCGCGGCGAGCGCAA
CCTGCTGTACGTGGTGGTGGTGGACACCGACGGCACCATCCTGGAGCAGATCAGCCTGAACA
GCATCATCAACAACGAGTACAGCATCGAGACCGACTACCACAAGCTGCTGGACGAGAAGGAG
GGCGACCGCGACCGCGCCCGCAAGAACTGGACCACCATCGAGAACATCAAGGAGCTGAAGGA
GGGCTACCTGAGCCAGGTGGTGAACGTGATCGCCAAGCTGGTGCTGAAGTACAACGCCATCA
TCTGCCTGGAGGACCTGAACTTCGGCTTCAAGCgcggccgcCAGAAGGTGGAGAAGCAGGTG
TACCAGAAGTTCGAGAAGATGCTGATCGACAAGCTGAACTACCTGGTGATCGACAAGAGCCG
CAAGCAGGACAAGCCCGAGGAGTTCGGCGGCGCCCTGAACGCCCTGCAGCTGACCAGCAAGT
TCACCAGCTTCAAGGACATGGGCAAGCAGACCGGCATCATCTACTACGTGCCCGCCTACCTG
ACCAGCAAGATCGACCCCACCACCGGCTTCGCCAACCTGTTCTACGTGAAGTACGAGAACGT
GGAGAAGGCCAAGGAGTTCTTCAGCCGCTTCGACAGCATCAGCTACAACAACGAGAGCGGCT
ACTTCGAGTTCGCCTTCGACTACAAGAAGTTCACCGACCGCGCCTGCGGCGCCCGCAGCCAG
TGGACCGTGTGCACCTACGGCGAGCGCATCATCAAGTTCCGCAACACCGAGAAGAACAACAG
CTTCGACGACAAGACCATCGTGCTGAGCGAGGAGTTCAAGGAGCTGTTCAGCATCTACGGCA
TCAGCTACGAGGACGGCGCCGAGCTGAAGAACAAGATCATGAGCGTGGACGAGGCCGACTTC
TTCCGCAGCCTGACCCGCCTGTTCCAGCAGACCATGCAGATGCGCAACAGCAGCAACGACGT
GACCCGCGACTACATCATCAGCCCCATCATGAACGACCGCGGCGAGTTCTTCAACAGCGAGG
CCTGCGACGCCAGCAAGCCCAAGGACGCCGACGCCAACGGCGCCTTCAACATCGCCCGCAAG
GGCCTGTGGGTGCTGGAGCAGATCCGCAACACCCCCAGCGGCGACAAGCTGAACCTGGCCAT
GAGCAACGCCGAGTGGCTGGAGTACGCCCAGCGCAACCAGATC

PrCas12a protein sequence

MIIGRDFNMYYQNLTKMYPISKTLRNELIPVGKTLENIRKNGILEADIQRKADYEHVKKLMD
NYHKQLINEALQGVHLSDLSDAYDLYFNLSKEKNSVDAFSKCQDKLRKEIVSLLKNHENFPK
IGNKEIIKLLQSLYDNDTDYKALDSFSNFYTYFSSYNEVRKNLYSDEEKSSTVAYRLINENL
PKFLDNIKAYAIAKKAGVRAEGLSEEDQDCLFIIETFERTLTQDGIDNYNAAIGKLNTAINL
FNQQNKKQEGFRKVPQMKCLYKQILSDREEAFIDEFSDDEDLITNIESFAENMNVFLNSEII
TDFKIALVESDGSLVYIKNDVSKTSFSNIVFGSWNAIDEKLSDEYDLANSKKKKDEKYYEKR
QKELKKNKSYDLETIIGLFDDNSDVIGKYIEKLESDITAIAEAKNDFDEIVLRKHDKNKSLR
KNTNAVEAIKSYLDTVKDFERDIKLINGSGQEVEKNLVVYAEQENILAEIKNVDSLYNMSRN
YLTQKPFSTEKFKLNFNRATLLNGWDKNKETDNLGILFEKDGMYYLGIMNTKANKIFVNIPK
ATSNDVYHKVNYKLLPGPNKMLPKVFFAQSNLDYYKPSEELLAKYKAGTHKKGDNFSLEDCH
ALIDFFKASIEKHPDWSSFGFEFSETCTYEDLSGFYREVEKQGYKITYTDVDADYITSLVER
DELYLFQIYNKDFSPYSKGNLNLHTIYLQMLFDQRNLNNVVYKLNGEAEVFYRPASINDEEV
IIHKAGEEIKNKNSKRAVDKPTSKFGYDIIKDRRYSKDKFMLHIPVTMNFGVDETRRFNDVV
NDALRNDEKVRVIGIDRGERNLLYVVVVDTDGTILEQISLNSIINNEYSIETDYHKLLDEKE
GDRDRARKNWTTIENIKELKEGYLSQVVNVIAKLVLKYNAIICLEDLNFGFKRGRQKVEKQV
YQKFEKMLIDKLNYLVIDKSRKQDKPEEFGGALNALQLTSKFTSFKDMGKQTGIIYYVPAYL
TSKIDPTTGFANLFYVKYENVEKAKEFFSRFDSISYNNESGYFEFAFDYKKFTDRACGARSQ
WTVCTYGERIIKFRNTEKNNSFDDKTIVLSEEFKELFSIYGISYEDGAELKNKIMSVDEADF
FRSLTRLFQQTMQMRNSSNDVTRDYIISPIMNDRGEFFNSEACDASKPKDADANGAFNIARK
GLWVLEQIRNTPSGDKLNLAMSNAEWLEYAQRNQI

Humanized Cas12a coding sequence derived from *Proteocatella sphenisci* DSM 23131 (GeneBank ID: NZ_KE384028.1)

GAGAACTTCAAGAACCTGTACCCCATCAACAAGACCCTGCGCTTCGAGCTGCGCCCCTACGG
CAAGACCCTGGAGAACTTCAAGAAGAGCGGCCTGCTGGAGAAGGACGCCTTCAAGGCCAACA
GCCGCCGCAGCATGCAGGCCATCATCGACGAGAAGTTCAAGGAGACCATCGAGGAGCGCCTG
AAGTACACCGAGTTCAGCGAGTGCGACCTGGGCAACATGACCAGCAAGGACAAGAAGATCAC
CGACAAGGCCGCCACCAACCTGAAGAAGCAGGTGATCCTGAGCTTCGACGACGAGATCTTCA
ACAACTACCTGAAGCCCGACAAGAACATCGACGCCCTGTTCAAGAACGACCCCAGCAACCCC
GTGATCAGCACCTTCAAGGGCTTCACCACCTACTTCGTGAACTTCTTCGAGATCCGCAAGCA
CATCTTCAAGGGCGAGAGCAGCGGCAGCATGGCCTACCGCATCATCGACGAGAACCTGACCA
CCTACCTGAACAACATCGAGAAGATCAAGAAGCTGCCCGAGGAGCTGAAGAGCCAGCTGGAG
GGCATCGACCAGATCGACAAGCTGAACAACTACAACGAGTTCATCACCCAGAGCGGCATCAC
CCACTACAACGAGATCATCGGCGGCATCAGCAAGAGCGAGAACGTGAAGATCCAGGGCATCA
ACGAGGGCATCAACCTGTACTGCCAGAAGAACAAGGTGAAGCTGCCCCGCCTGACCCCCCTG
TACAAGATGATCCTGAGCGACCGCGTGAGCAACAGCTTCGTGCTGGACACCATCGAGAACGA
CACCGAGCTGATCGAGATGATCAGCGACCTGATCAACAAGACCGAGATCAGCCAGGACGTGA
TCATGAGCGACATCCAGAACATCTTCATCAAGTACAAGCAGCTGGGCAACCTGCCCGGCATC
AGCTACAGCAGCATCGTGAACGCCATCTGCAGCGACTACGACAACAACTTCGGCGACGGCAA
GCGCAAGAAGAGCTACGAGAACGACCGCAAGAAGCACCTGGAGACCAACGTGTACAGCATCA
ACTACATCAGCGAGCTGCTGACCGACACCGACGTGAGCAGCAACATCAAGATGCGCTACAAG
GAGCTGGAGCAGAACTACCAGGTGTGCAAGGAGAACTTCAACGCCACCAACTGGATGAACAT
CAAGAACATCAAGCAGAGCGAGAAGACCAACCTGATCAAGGACCTGCTGGACATCCTGAAGA
GCATCCAGCGCTTCTACGACCTGTTCGACATCGTGGACGAGGACAAGAACCCCAGCGCCGAG

TTCTACACCTGGCTGAGCAAGAACGCCGAGAAGCTtGACTTCGAGTTCAACAGCGTGTACAA
CAAGAGCCGCAACTACCTGACCCGCAAGCAGTACAGCGACAAGAAGATCAAGCTGAACTTCG
ACAGCCCCACCCTGGCCAAGGGCTGGGACGCCAACAAGGAGATCGACAACAGCACCATCATC
ATGCGCAAGTTCAACAACGACCGCGGCGACTACGACTACTTCCTGGGCATCTGGAACAAGAG
CACCCCCGCCAACGAGAAGATCATCCCCCTGGAGGACAACGGCCTGTTCGAGAAGATGCAGT
ACAAGCTGTACCCCGACCCCAGCAAGATGCTGCCCAAGCAGTTCCTGAGCAAGATCTGGAAG
GCCAAGCACCCCACCACCCCCGAGTTCGACAAGAAGTACAAGGAGGGCCGCCACAAGAAGGG
CCCCGACTTCGAGAAGGAGTTCCTGCACGAGCTGATCGACTGCTTCAAGCACGGCCTGGTGA
ACCACGACGAGAAGTACCAGGACGTGTTCGGCTTCAACCTGCGCAACACCGAGGACTACAAC
AGCTACACCGAGTTCCTGGAGGACGTGGAGCGCTGCAACTACAACCTGAGCTTCAACAAGAT
CGCCGACACCAGCAACCTGATCAACGACGGCAAGCTGTACGTGTTCCAGATCTGGAGCAAGG
ACTTCAGCATCGACAGCAAGGGCACCAAGAACCTGAACACCATCTACTTCGAGAGCCTGTTC
AGCGAGGAGAACATGATCGAGAAGATGTTCAAGCTGAGCGGCGAGGCCGAGATCTTCTACCG
CCCCGCCAGCCTGAACTACTGCGAGGACATCATCAAGAAGGGCCACCACCACGCCGAGCTGA
AGGACAAGTTCGACTACCCCATCATCAAGGACAAGCGCTACAGCCAGGACAAGTTCTTCTTC
CACGTGCCCATGGTGATCAACTACAAGAGCGAGAAGCTGAACAGCAAGAGCCTGAACAACCG
CACCAACGAGAACCTGGGCCAGTTCACCCACATCATCGGCATCGACCGCGGCGAGCGCCACC
TGATCTACCTGACCGTGGTGGACGTGAGCACCGGCGAGATCGTGGAGCAGAAGCACCTGGAC
GAGATCATCAACACCGACACCAAGGGCGTGGAGCACAAGACCCACTACCTGAACAAGCTGGA
GGAGAAGAGCAAGACCCGCGACAACGAGCGCAAGAGCTGGGAGGCCATCGAGACCATCAAGG
AGCTGAAGGAGGGCTACATCAGCCACGTGATCAACGAGATCCAGAAGCTGCAGGAGAAGTAC
AACGCCCTGATCGTGATGGAGAACCTGAACTACGGCTTCAAGAACAGCCGCATCAAGGTGGA
GAAGCAGGTGTACCAGAAGTTCGAGACCGCCCTGATCAAGAAGTTCAACTACATCATCGACA
AGAAGGACCCCGAGACCTACATCCACGGCTACCAGCTGACCAACCCCATCACCACCCTGGAC
AAGATCGGCAACCAGAGCGGCATCGTGCTGTACATCCCCGCCTGGAACACCAGCAAGATCGA
CCCCGTGACCGGCTTCGTGAACCTGCTGTACGCCGACGACCTGAAGTACAAGAACCAGGAGC
AGGCCAAGAGCTTCATCCAGAAGATCGACAACATCTACTTCGAGAACGGCGAGTTCAAGTTC
GACATCGACTTCAGCAAGTGGAACAACCGCTACAGCATCAGCAAGACCAAGTGGACCCTGAC
CAGCTACGGCACCCGCATCCAGACCTTCCGCAACCCCCAGAAGAACAACAAGTGGGACAGCG
CCGAGTACGACCTGACCGAGGAGTTCAAGCTGATCCTGAACATCGACGGCACCCTGAAGAGC
CAGGACGTGGAGACCTACAAGAAGTTCATGAGCCTGTTCAAGCTGATGCTGCAGCTGCGCAA
CAGCGTGACCGGCACCGACATCGACTACATGATCAGCCCCGTGACCGACAAGACCGGCACCC
ACTTCGACAGCCGCGAGAACATCAAGAACCTGCCCGCCGACGCCGACGCCAACGGCGCCTAC
AACATCGCCCGCAAGGGCATCATGGCCATCGAGAACATCATGAACGGCATCAGCGACCCCCT
GAAGATCAGCAACGAGGACTACCTGAAGTACATCCAGAACCAGCAGGAG

PsCas12a protein sequence

ENFKNLYPINKTLRFELRPYGKTLENFKKSGLLEKDAFKANSRRSMQAIIDEKFKETIEERL
KYTEFSECDLGNMTSKDKKITDKAATNLKKQVILSFDDEIFNNYLKPDKNIDALFKNDPSNP
VISTFKGFTTYFVNFFEIRKHIFKGESSGSMAYRIIDENLTTYLNNIEKIKKLPEELKSQLE
GIDQIDKLNNYNEFITQSGITHYNEIIGGISKSENVKIQGINEGINLYCQKNKVKLPRLTPL
YKMILSDRVSNSFVLDTIENDTELIEMISDLINKTEISQDVIMSDIQNIFIKYKQLGNLPGI
SYSSIVNAICSDYDNNFGDGKRKKSYENDRKKHLETNVYSINYISELLTDTDVSSNIKMRYK
ELEQNYQVCKENFNATNWMNIKNIKQSEKTNLIKDLLDILKSIQRFYDLFDIVDEDKNPSAE

FYTWLSKNAEKLDFEFNSVYNKSRNYLTRKQYSDKKIKLNFDSPTLAKGWDANKEIDNSTII
MRKFNNDRGDYDYFLGIWNKSTPANEKIIPLEDNGLFEKMQYKLYPDPSKMLPKQFLSKIWK
AKHPTTPEFDKKYKEGRHKKGPDFEKEFLHELIDCFKHGLVNHDEKYQDVFGFNLRNTEDYN
SYTEFLEDVERCNYNLSFNKIADTSNLINDGKLYVFQIWSKDFSIDSKGTKNLNTIYFESLF
SEENMIEKMFKLSGEAEIFYRPASLNYCEDIIKKGHHHAELKDKFDYPIIKDKRYSQDKFFF
HVPMVINYKSEKLNSKSLNNRTNENLGQFTHIIGIDRGERHLIYLTVVDVSTGEIVEQKHLD
EIINTDTKGVEHKTHYLNKLEEKSKTRDNERKSWEAIETIKELKEGYISHVINEIQKLQEKY
NALIVMENLNYGFKNSRIKVEKQVYQKFETALIKKFNYIIDKKDPETYIHGYQLTNPITTLD
KIGNQSGIVLYIPAWNTSKIDPVTGFVNLLYADDLKYKNQEQAKSFIQKIDNIYFENGEFKF
DIDFSKWNNRYSISKTKWTLTSYGTRIQTFRNPQKNNKWDSAEYDLTEEFKLILNIDGTLKS
QDVETYKKFMSLFKLMLQLRNSVTGTDIDYMISPVTDKTGTHFDSRENIKNLPADADANGAY
NIARKGIMAIENIMNGISDPLKISNEDYLKYIQNQQE

Humanized Cas12a coding sequence derived from *Pseudobutyrivibrio xylanivorans* strain DSM 10317 (GeneBank ID: FMWK01000002.1)

ATGATCATCGGCCGCGACTTCAACATGTACTACCAGAACCTGACCAAGATGTACCCCATCAG
CAAGACCCTGCGCAACGAGCTGATCCCCGTGGGCAAGACCCTGGAGAACATCCGCAAGAACG
GCATCCTGGAGGCCGACATCCAGCGCAAGGCCGACTACGAGCACGTGAAGAAGCTGATGGAC
AACTACCACAAGCAGCTGATCAACGAGGCCCTGCAGGGCGTGCACCTGAGCGACCTGAGCGA
CGCCTACGACCTGTACTTCAACCTGAGCAAGGAGAAGAACAGCGTGGACGCCTTCAGCAAGT
GCCAGGACAAGCTGCGCAAGGAGATCGTGAGCTTCCTGAAGAACCACGAGAACTTCCCCAAG
ATCGGCAACAAGGAGATCATCAAGCTGATCCAGAGCCTGAACGACAACGACGCCGACAACAA
CGCCCTGGACAGCTTCAGCAACTTCTACACCTACTTCAGCAGCTACAACGAGGTGCGCAAGA
ACCTGTACAGCGACGAGGAGAAGAGCAGCACCGTGGCCTACCGCCTGATCAACGAGAACCTG
CCCAAGAGCCTGGACAACATCAAGGCCTACGCCATCGCCAAGAAGGCCGGCGTGCGCGCCGA
GGGCCTGAGCGAGGAGGAGCAGGACTGCCTGTTCATCATCGAGACCTTCGAGCGCACCCTGA
CCCAGGACGGCATCGACAACTACAACGCCGACATCGGCAAGCTGAACACCGCCATCAACCTG
TACAACCAGCAGAACAAGAAGCAGGAGGGCTTCCGCAAGGTGCCCCAGATGAAGTGCCTGTA
CAAGCAGATCCTGAGCGACCGCGAGGAGGCCTTCATCGACGAGTTCAGCGACGACGAGGACC
TGATCACCAACATCGAGAGCTTCGCCGAGAACATGAACGTGTTCCTGAACAGCGAGATCATC
ACCGACTTCAAGAACGCCCTGGTGGAGAGCGACGGCAGCCTGGTGTACATCAAGAACGACGT
GAGCAAGACCCTGTTCAGCAACATCGTGTTCGGCAGCTGGAACGCCATCGACGAGAAGCTGA
GCGACGAGTACGACCTGGCCAACAGCAAGAAGAAGAAGGACGAGAagtactACGAGAAGCGC
CAGAAGGAGCTGAAGAAGAACAAGAGCTACGACCTGGAGACCATCATCGGCCTGTTCGACGA
CAGCATCGACGTGATCGGCAAGTACATCGAGAAGCTGGAGAGCGACATCACCGCCATCGCCG
AGGCCAAGAACGACTTCGACGAGATCGTGCTGCGCAAGCACGACAAGAACAAGAGCCTGCGC
AAGAACACCAACGCCGTGGAGGCCATCAAGAGCTACCTGGACACCGTGAAGGACTTCGAGCG
CGACATCAAGCTGATCAACGGCAGCGGCCAGGAGGTGGAGAAGAACCTGGTGGTGTACGCCG
AGCAGGAGAACATCCTGGCCGAGATCAAGAACGTGGACAGCCTGTACAACATGAGCCGCAAC
TACCTGACCCAGAAGCCCTTCAGCACCGAGAAGTTCAAGCTGAACTTCGAGAACCCCACCCT
GCTGAACGGCTGGGACCGCAACAAGGAGAAGGACTACCTGGGCATCCTGTTCGAGAAGGAGG
GCATGTACTACCTGGGCATCATCAACAACAACCACCGCAAGATCTTCGAGAACGAGAAGCTG
TGCACCGGCAAGGAGAGCTGCTTCAACAAGATCGTGTACAAGCAGATCAGCAACGCCGCCAA
GTACCTGAGCAGCAAGCAGATCAACCCCCAGAACCCCCCCAAGGAGATCGCCGAGATCCTGC

TGAAGCGCAAGGCCGACAGCAGCAGCCTGAGCCGCAAGGAGACCGAGCTGTTCATCGACTAC
CTGAAGGACGACTTCCTGGTGAACTACCCCATGATCATCAACAGCGACGGCGAGAACTTCTT
CAACTTCCACTTCAAGCAGGCCAAGGACTACGGCAGCCTGCAGGAGTTCTTCAAGGAGGTGG
AGCACCAGGCCTACAGCCTGAAGACCCGCCCCATCGACGACAGCTACATCTACCGCATGATC
GACGAGGGCAAGCTGTACCTGTTCCAGATCCACAACAAGGACTTCAGCCCCTACAGCAAGGG
CAACCTGAACCTGCACACCATCTACCTGCAGATGCTGTTCGACCAGCGCAACCTGAACAACG
TGGTGTACAAGCTGAACGGCGAGGCCGAGGTGTTCTACCGCCCCGCCAGCATCAACGACGAG
GAGGTGATCATCCACAAGGCCGGCGAGGAGATCAAGAACAAGAACAGCAAGCGCGCCGTGGA
CAAGCCCACCAGCAAGTTCGGCTACGACATCATCAAGGACCGCCGCTACAGCAAGGACAAGT
TCATGCTGCACATCCCCGTGACCATGAACTTCGGCGTGGACGAGACCCGCCGCTTCAACGAC
GTGGTGAACGACGCCCTGCGCAACGACGAGAAGGTGCGCGTGATCGGCATCGACCGCGGCGA
GCGCAACCTGCTGTACGTGGTGGTGGACACCGACGGCACCATCCTGGAGCAGATCAGCC
TGAACAGCATCATCAACAACGAGTACAGCATCGAGACCGACTACCACAAGCTGCTGGACGAG
AAGGAGGGCGACCGCGACCGCGCCCGCAAGAACTGGACCACCATCGAGAACATCAAGGAGCT
GAAGGAGGGCTACCTGAGCCAGGTGGTGAACGTGATCGCCAAGCTGGTGCTGAAGTACAACG
CCATCATCTGCCTGGAGGACCTGAACTTCGGCTTCAAGCgcggccgcCAGAAGGTGGAGAAG
CAGGTGTACCAGAAGTTCGAGAAGATGCTGATCGACAAGCTGAACTACCTGGTGATCGACAA
GAGCCGCAAGCAGGAGAAGCCCGAGGAGTTCGGCGGCGCCCTGAACGCCCTGCAGCTGACCA
GCAAGTTCACCAGCTTCAAGGACATGGGCAAGCAGACCGGCATCATCTACTACGTGCCCGCC
TACCTGACCAGCAAGATCGACCCCACCACCGGCTTCGCCAACCTGTTCTACGTGAAGTACGA
GAACGTGGAGAAGGCCAAGGAGTTCTTCAGCCGCTTCGACAGCATCAGCTACAACAACGAGA
GCGGCTACTTCGAGTTCGCCTTCGACTACAAGAAGTTCACCGACCGCGCCTGCGGCGCCCGC
AGCCAGTGGACCGTGTGCACCTACGGCGAGCGCATCATCAAGTACCGCAACGCCGACAAGAA
CAACAGCTTCGACGACAAGACCATCGTGCTGAGCGAGGAGTTCAAGGAGCTGTTCAGCATCT
ACGGCATCAGCTACGAGGACGGCGCCGAGCTGAAGAACAAGATCATGAGCGTGGACGAGGCC
GACTTCTTCCGCTGCCTGACCGGCCTGCTGCAGAAGACCCTGCAGATGCGCAACAGCAGCAA
CGACGGCACCCGCGACTACATCATCAGCCCCATCATGAACGACCGCGGCGAGTTCTTCAACA
GCGAGGCCTGCGACGCCAGCAAGCCCAAGGACGCCGACGCCAACGGCGCCTTCAACATCGCC
CGCAAGGGCCTGTGGGTGCTGGAGCAGATCCGCAACACCCCCAGCGGCGACAAGCTGAACCT
GGCCATGAGCAACGCCGAGTGGCTGGAGTACGCCCAGCGCAACCAGATC

PxCas12a protein sequence

MIIGRDFNMYYQNLTKMYPISKTLRNELIPVGKTLENIRKNGILEADIQRKADYEHVKKLMD
NYHKQLINEALQGVHLSDLSDAYDLYFNLSKEKNSVDAFSKCQDKLRKEIVSFLKNHENFPK
IGNKEIIKLIQSLNDNDADNNALDSFSNFYTYFSSYNEVRKNLYSDEEKSSTVAYRLINENL
PKSLDNIKAYAIAKKAGVRAEGLSEEEQDCLFIIETFERTLTQDGIDNYNADIGKLNTAINL
YNQQNKKQEGFRKVPQMKCLYKQILSDREEAFIDEFSDDEDLITNIESFAENMNVFLNSEII
TDFKNALVESDGSLVYIKNDVSKTLFSNIVFGSWNAIDEKLSDEYDLANSKKKKDEKYYEKR
QKELKKNKSYDLETIIGLFDDSIDVIGKYIEKLESDITAIAEAKNDFDEIVLRKHDKNKSLR
KNTNAVEAIKSYLDTVKDFERDIKLINGSGQEVEKNLVVYAEQENILAEIKNVDSLYNMSRN
YLTQKPFSTEKFKLNFENPTLLNGWDRNKEKDYLGILFEKEGMYYLGIINNNHRKIFENEKL
CTGKESCFNKIVYKQISNAAKYLSSKQINPQNPPKEIAEILLKRKADSSSLSRKETELFIDY
LKDDFLVNYPMIINSDGENFFNFHFKQAKDYGSLQEFFKEVEHQAYSLKTRPIDDSYIYRMI
DEGKLYLFQIHNKDFSPYSKGNLNLHTIYLQMLFDQRNLNNVVYKLNGEAEVFYRPASINDE

EVIIHKAGEEIKNKNSKRAVDKPTSKFGYDIIKDRRYSKDKFMLHIPVTMNFGVDETRRFND
VVNDALRNDEKVRVIGIDRGERNLLYVVVVDTDGTILEQISLNSIINNEYSIETDYHKLLDE
KEGDRDRARKNWTTIENIKELKEGYLSQVVNVIAKLVLKYNAIICLEDLNFGFKRGRQKVEK
QVYQKFEKMLIDKLNYLVIDKSRKQEKPEEFGGALNALQLTSKFTSFKDMGKQTGIIYYVPA
YLTSKIDPTTGFANLFYVKYENVEKAKEFFSRFDSISYNNESGYFEFAFDYKKFTDRACGAR
SQWTVCTYGERIIKYRNADKNNSFDDKTIVLSEEFKELFSIYGISYEDGAELKNKIMSVDEA
DFFRCLTGLLQKTLQMRNSSNDGTRDYIISPIMNDRGEFFNSEACDASKPKDADANGAFNIA
RKGLWVLEQIRNTPSGDKLNLAMSNAEWLEYAQRNQI

Humanized Cas12a coding sequence derived from *Sneathia amnii* strain SN35 (GeneBank ID: CP011280.1)

ATGAATGATATTGAAGGATTAAAAGAAGAATTCTTAAAAATTAGTTTAGAAAATTTTGAAGG
TATATATATATCAAACAAGAAATTAAACGAGATTAGTAATAGAAAATTTGGGGATTATAATT
CAATTAATATGATGATTAAACAGTCTATGAATGAAAAAGGTATATTAAGTAAGAAAGAAATT
AATGAATTAATTCCAGATTTAGAAAATATAAACAAACCAAAAGTTAAAAGTTTTAATTTAAG
CTTTATTTTTGAAAATTTAACAAAAGAACATAAAGAACTTATTATAGATTACATAAGAGAAA
ATATATGTAATGTCATAGAAAATGTAAAAATAACTATAGAAAAATATAGAAATATTGATAAT
AAAATAGAATTTAAAAATAATGCAGAAAAAGTTTCTAAAATAAAAGAAATGTTAGAATCAAT
AAATGAATTGTGTAAACTTATAAAAGAATTTAATACTGATGAAATTGAAAAAAATAATGAAT
TCTATAATATATTAAATAAAAATTTTGAAATTTTTGAAAGTTCATATAAAGTTTTAAATAAA
GTAAGAAATTTTGTTACAAAAAAAGAAGTTATAGAAAACAAGATGAAATTAAATTTTTCTAA
CTATCAACTTGGAAATGGATGGCATAAAAATAAAGAAAAAGATTGTAGTATTATTTTGTTTA
GAAAGAGAAATAATGAGAGATGGATATATTATTTAGGTATTTTAAAACATGGAACTAAAATA
AAAGAAAATGACTATTTGTCAAGTGTAGATACAGGATTTTATAAAATGGATTATTATGCACA
AAATTCATTAAGTAAAATGATACCTAAATGCAGTATTACCGTAAAAAATGTAAAAAATGCTC
CAGAAGATGAAAGTGTTATTTTAAATGATAGCAAAAAATTCAATGAACCTTTAGAAATAACA
CCAGAAATAAGAAAGTTGTATGGAAATAATGAACATATAAAAGGAGATAAGTTTAAAAAAGA
ATCTTTAGTAAAATGGATAGATTTTTGTAAAGAATTTTTACTTAAATATAAATCATTTGAGA
AGGCAAAGAAAGAAATTTTAAAGTTAAAAGAAAGTAATTTGTATGAAAATTTAGAGGAATTT
TATTCTGATGCAGAGGAAAAAGCTTACTTTTTAGAATTTATAAATATAGATGAAGATAAAAT
AAAAAAACTTGTTAAAGAAAAAAATCTATATCTATTCCAAATATATAATAAAGATTTTTCTG
CGTACAGTACAGGGAATAAAAATTTACATACTATGTATTTTGAAGAATTATTTACAGATGAA
AATTTAAAAAAACCAGTTTTTAAATTAAATGGAAATACGGAAGTCTTTTATAGAATAGCTTC
ATCTAAACCTAAAATAGTTCACAATAAAGGAGAAAAATTAGTAAATAAAACATATTTAGATG
ATGGCATAATAAAAACTATACCAGATAGTGTATATGAAGAAATTTCTGAAAAAGTAAAAAAT
AATGAAGATTATTCAAAATTACTAGAAGAAAATAATATAAAAAATCTTGAAATAAAAGTAGC
TACTCATGAAATAGTAAAGGATAAAAGATATTTTGAAAATAAATTTTTATTTTATCTACCAA
TAACTTTAAATAAAAAAGTATCAAATAAAAATACTAATAAAAATATTAATAAAAATGTTATA
GATGAAATAAAGGATTGTAATGAATATAATGTGATAGGTATAGATAGGGGTGAAAGAAATTT
AATAAGTCTTTGTATAATAAATCAAAATGGAGAAATTATACTACAAAAAGAAATGAATATTA
TTCAGTCATCAGATAAATATAATGTCGATTATAATGAAAAATTAGAAATAAAATCTAAAGAA
AGAGATAATGCTAAGAAAAATTGGAGTGAAATTGGAAAAATAAAGGATTTGAAATCAGGGTA
TTTATCAGCAGTAGTTCATGAAATAGTAAAGCTCGCTATTGAGTACAATGCCGTCATAATAT
TAGAAGATTTAAATAATGGCTTTAAAAATTCAAGAAAAAAAGTTGATAAACAAATATATCAA

AAATTTGAAAGAGCTTTAATTGAAAAATTGCAATTCTTAATATTTAAAAATTATGATAAAAA
TGAAAAGGGAGGACTTAGAAATGCATTTCAATTAACACCAGAATTGAAAAATATTACTAAAG
TTGCATCTCAACAAGGTATAATAATTTATACTAATCCAGCATATACTTCTAAAATAGATCCT
ACTACAGGTTATGCGAATATTATAAAAAAATCAAATAATAACGAAGAATCTATAGTAAAAGC
TATTGATAAAATATCTTATGATAAAGAAAAAGATATGTTCTATTTTGATATTAATTTATCAA
ATAGTAGTTTTAATTTGACAGTAAAAAATGTTTTAAAGAAAGAATGGAGAATATATACTAAT
GGTGAAAGAATAATATATAAAGATAGGAAATATATTACATTAAACATAACTCAAGAAATGAA
AGATATTTTAAGTAAATGTGGTATTGATTATTTAAATATTGATAATCTAAAACAAGATATAT
TAAAAAATAAACTACATAAAAAAGTTTATTATATATTTGAATTAGCAAATAAAATGAGAAAT
GAAAATAAAGATGTTGATTATATTATATCTCCAGTATTAAATAAAGATGGAAAATTCTTTAT
GACACAAGAAATTAATGAGCTAACTCCGAAAGATGCCGATTTAAATGGTGCATATAATATTG
CACTAAAAGGTAAATTAATGATTGATAATTTAAACAAAAAAGAAAAATTTGTATTTTTAAGT
AATGAAGATTGGTTAAACTTTATTCAAGGAAGA

SaCas12a protein sequence

MNDIEGLKEEFLKISLENFEGIYISNKKLNEISNRKFGDYNSINMMIKQSMNEKGILSKKEI
NELIPDLENINKPKVKSFNLSFIFENLTKEHKELIIDYIRENICNVIENVKITIEKYRNIDN
KIEFKNNAEKVSKIKEMLESINELCKLIKEFNTDEIEKNNEFYNILNKNFEIFESSYKVLNK
VRNFVTKKEVIENKMKLNFSNYQLGNGWHKNKEKDCSIILFRKRNNERWIYYLGILKHGTKI
KENDYLSSVDTGFYKMDYYAQNSLSKMIPKCSITVKNVKNAPEDESVILNDSKKFNEPLEIT
PEIRKLYGNNEHIKGDKFKKESLVKWIDFCKEFLLKYKSFEKAKKEILKLKESNLYENLEEF
YSDAEEKAYFLEFINIDEDKIKKLVKEKNLYLFQIYNKDFSAYSTGNKNLHTMYFEELFTDE
NLKKPVFKLNGNTEVFYRIASSKPKIVHNKGEKLVNKTYLDDGIIKTIPDSVYEEISEKVKN
NEDYSKLLEENNIKNLEIKVATHEIVKDKRYFENKFLFYLPITLNKKVSNKNTNKNINKNVI
DEIKDCNEYNVIGIDRGERNLISLCIINQNGEIILQKEMNIIQSSDKYNVDYNEKLEIKSKE
RDNAKKNWSEIGKIKDLKSGYLSAVVHEIVKLAIEYNAVIILEDLNNGFKNSRKKVDKQIYQ
KFERALIEKLQFLIFKNYDKNEKGGLRNAFQLTPELKNITKVASQQGIIIYTNPAYTSKIDP
TTGYANIIKKSNNNEESIVKAIDKISYDKEKDMFYFDINLSNSSFNLTVKNVLKKEWRIYTN
GERIIYKDRKYITLNITQEMKDILSKCGIDYLNIDNLKQDILKNKLHKKVYYIFELANKMRN
ENKDVDYIISPVLNKDGKFFMTQEINELTPKDADLNGAYNIALKGKLMIDNLNKKEKFVFLS
NEDWLNFIQGR

Humanized Cas12a coding sequence derived from *Acidaminococcus sp*. BV3L6 (GeneBank ID: AWUR01000016.1)

ATGACACAGTTCGAGGGCTTTACCAACCTGTATCAGGTGAGCAAGACACTGCGGTTTGAGCT
GATCCCACAGGGCAAGACCCTGAAGCACATCCAGGAGCAGGGCTTCATCGAGGAGGACAAGG
CCCGCAATGATCACTACAAGGAGCTGAAGCCCATCATCGATCGGATCTACAAGACCTATGCC
GACCAGTGCCTGCAGCTGGTGCAGCTGGATTGGGAGAACCTGAGCGCCGCCATCGACTCCTA
TAGAAAGGAGAAAACCGAGGAGACAAGGAACGCCCTGATCGAGGAGCAGGCCACATATCGCA
ATGCCATCCACGACTACTTCATCGGCCGGACAGACAACCTGACCGATGCCATCAATAAGAGA
CACGCCGAGATCTACAAGGGCCTGTTCAAGGCCGAGCTGTTTAATGGCAAGGTGCTGAAGCA
GCTGGGCACCGTGACCACAACCGAGCACGAGAACGCCCTGCTGCGGAGCTTCGACAAGTTTA
CAACCTACTTCTCCGGCTTTTATGAGAACAGGAAGAACGTGTTCAGCGCCGAGGATATCAGC
ACAGCCATCCCACACCGCATCGTGCAGGACAACTTCCCCAAGTTTAAGGAGAATTGTCACAT

CTTCACACGCCTGATCACCGCCGTGCCCAGCCTGCGGGAGCACTTTGAGAACGTGAAGAAGG
CCATCGGCATCTTCGTGAGCACCTCCATCGAGGAGGTGTTTTCCTTCCCTTTTTATAACCAG
CTGCTGACACAGACCCAGATCGACCTGTATAACCAGCTGCTGGGAGGAATCTCTCGGGAGGC
AGGCACCGAGAAGATCAAGGGCCTGAACGAGGTGCTGAATCTGGCCATCCAGAAGAATGATG
AGACAGCCCACATCATCGCCTCCCTGCCACACAGATTCATCCCCCTGTTTAAGCAGATCCTG
TCCGATAGGAACACCCTGTCTTTCATCCTGGAGGAGTTTAAGAGCGACGAGGAAGTGATCCA
GTCCTTCTGCAAGTACAAGACACTGCTGAGAAACGAGAACGTGCTGGAGACAGCCGAGGCCC
TGTTTAACGAGCTGAACAGCATCGACCTGACACACATCTTCATCAGCCACAAGAAGCTGGAG
ACAATCAGCAGCGCCCTGTGCGACCACTGGGATACACTGAGGAATGCCCTGTATGAGCGGAG
AATCTCCGAGCTGACAGGCAAGATCACCAAGTCTGCCAAGGAGAAGGTGCAGCGCAGCCTGA
AGCACGAGGATATCAACCTGCAGGAGATCATCTCTGCCGCAGGCAAGGAGCTGAGCGAGGCC
TTCAAGCAGAAAACCAGCGAGATCCTGTCCCACGCACACGCCGCCCTGGATCAGCCACTGCC
TACAACCCTGAAGAAGCAGGAGGAGAAGGAGATCCTGAAGTCTCAGCTGGACAGCCTGCTGG
GCCTGTACCACCTGCTGGACTGGTTTGCCGTGGATGAGTCCAACGAGGTGGACCCCGAGTTC
TCTGCCCGGCTGACCGGCATCAAGCTGGAGATGGAGCCTTCTCTGAGCTTCTACAACAAGGC
CAGAAATTATGCCACCAAGAAGCCCTACTCCGTGGAGAAGTTCAAGCTGAACTTTCAGATGC
CTACACTGGCCTCTGGCTGGGACGTGAATAAGGAGAAGAACAATGGCGCCATCCTGTTTGTG
AAGAACGGCCTGTACTATCTGGGCATCATGCCAAAGCAGAAGGGCAGGTATAAGGCCCTGAG
CTTCGAGCCCACAGAGAAAACCAGCGAGGGCTTTGATAAGATGTACTATGACTACTTCCCTG
ATGCCGCCAAGATGATCCCAAAGTGCAGCACCCAGCTGAAGGCCGTGACAGCCCACTTTCAG
ACCCACACAACCCCCATCCTGCTGTCCAACAATTTCATCGAGCCTCTGGAGATCACAAAGGA
GATCTACGACCTGAACAATCCTGAGAAGGAGCCAAAGAAGTTTCAGACAGCCTACGCCAAGA
AAACCGGCGACCAGAAGGGCTACAGAGAGGCCCTGTGCAAGTGGATCGACTTCACAAGGGAT
TTTCTGTCCAAGTATACCAAGACAACCTCTATCGATCTGTCTAGCCTGCGGCCATCCTCTCA
GTATAAGGACCTGGGCGAGTACTATGCCGAGCTGAATCCCCTGCTGTACCACATCAGCTTCC
AGAGAATCGCCGAGAAGGAGATCATGGATGCCGTGGAGACAGGCAAGCTGTACCTGTTCCAG
ATCTATAACAAGGACTTTGCCAAGGGCCACCACGGCAAGCCTAATCTGCACACACTGTATTG
GACCGGCCTGTTTTCTCCAGAGAACCTGGCCAAGACAAGCATCAAGCTGAATGGCCAGGCCG
AGCTGTTCTACCGCCCTAAGTCCAGGATGAAGAGGATGGCACACCGGCTGGGAGAGAAGATG
CTGAACAAGAAGCTGAAGGATCAGAAAACCCCAATCCCCGACACCCTGTACCAGGAGCTGTA
CGACTATGTGAATCACAGACTGTCCCACGACCTGTCTGATGAGGCCAGGGCCCTGCTGCCCA
ACGTGATCACCAAGGAGGTGTCTCACGAGATCATCAAGGATAGGCGCTTTACCAGCGACAAG
TTCTTTTTCCACGTGCCTATCACACTGAACTATCAGGCCGCCAATTCCCCATCTAAGTTCAA
CCAGAGGGTGAATGCCTACCTGAAGGAGCACCCCGAGACACCTATCATCGGCATCGATCGGG
GCGAGAGAAACCTGATCTATATCACAGTGATCGACTCCACCGGCAAGATCCTGGAGCAGCGG
AGCCTGAACACCATCCAGCAGTTTGATTACCAGAAGAAGCTGGACAACAGGGAGAAGGAGAG
GGTGGCAGCAAGGCAGGCCTGGTCTGTGGTGGGCACAATCAAGGATCTGAAGCAGGGCTATC
TGAGCCAGGTCATCCACGAGATCGTGGACCTGATGATCCACTACCAGGCCGTGGTGGTGCTG
GAGAACCTGAATTTCGGCTTTAAGAGCAAGAGGACCGGCATCGCCGAGAAGGCCGTGTACCA
GCAGTTCGAGAAGATGCTGATCGATAAGCTGAATTGCCTGGTGCTGAAGGACTATCCAGCAG
AGAAAGTGGGAGGCGTGCTGAACCCATACCAGCTGACAGACCAGTTCACCTCCTTTGCCAAG
ATGGGCACCCAGTCTGGCTTCCTGTTTTACGTGCCTGCCCCATATACATCTAAGATCGATCC
CCTGACCGGCTTCGTGGACCCCTTCGTGTGGAAAACCATCAAGAATCACGAGAGCCGCAAGC
ACTTCCTGGAGGGCTTCGACTTTCTGCACTACGACGTGAAAACCGGCGACTTCATCCTGCAC

TTTAAGATGAACAGAAATCTGTCCTTCCAGAGGGGCCTGCCCGGCTTTATGCCTGCATGGGA
TATCGTGTTCGAGAAGAACGAGACACAGTTTGACGCCAAGGGCACCCCTTTCATCGCCGGCA
AGAGAATCGTGCCAGTGATCGAGAATCACAGATTCACCGGCAGATACCGGGACCTGTATCCT
GCCAACGAGCTGATCGCCCTGCTGGAGGAGAAGGGCATCGTGTTCAGGGATGGCTCCAACAT
CCTGCCAAAGCTGCTGGAGAATGACGATTCTCACGCCATCGACACCATGGTGGCCCTGATCC
GCAGCGTGCTGCAGATGCGGAACTCCAATGCCGCCACAGGCGAGGACTATATCAACAGCCCC
GTGCGCGATCTGAATGGCGTGTGCTTCGACTCCCGGTTTCAGAACCCAGAGTGGCCCATGGA
CGCCGATGCCAATGGCGCCTACCACATCGCCCTGAAGGGCCAGCTGCTGCTGAATCACCTGA
AGGAGAGCAAGGATCTGAAGCTGCAGAACGGCATCTCCAATCAGGACTGGCTGGCCTACATC
CAGGAGCTGCGCAAC

AsCas12a protein sequence

MTQFEGFTNLYQVSKTLRFELIPQGKTLKHIQEQGFIEEDKARNDHYKELKPIIDRIYKTYA
DQCLQLVQLDWENLSAAIDSYRKEKTEETRNALIEEQATYRNAIHDYFIGRTDNLTDAINKR
HAEIYKGLFKAELFNGKVLKQLGTVTTTEHENALLRSFDKFTTYFSGFYENRKNVFSAEDIS
TAIPHRIVQDNFPKFKENCHIFTRLITAVPSLREHFENVKKAIGIFVSTSIEEVFSFPFYNQ
LLTQTQIDLYNQLLGGISREAGTEKIKGLNEVLNLAIQKNDETAHIIASLPHRFIPLFKQIL
SDRNTLSFILEEFKSDEEVIQSFCKYKTLLRNENVLETAEALFNELNSIDLTHIFISHKKLE
TISSALCDHWDTLRNALYERRISELTGKITKSAKEKVQRSLKHEDINLQEIISAAGKELSEA
FKQKTSEILSHAHAALDQPLPTTLKKQEEKEILKSQLDSLLGLYHLLDWFAVDESNEVDPEF
SARLTGIKLEMEPSLSFYNKARNYATKKPYSVEKFKLNFQMPTLASGWDVNKEKNNGAILFV
KNGLYYLGIMPKQKGRYKALSFEPTEKTSEGFDKMYYDYFPDAAKMIPKCSTQLKAVTAHFQ
THTTPILLSNNFIEPLEITKEIYDLNNPEKEPKKFQTAYAKKTGDQKGYREALCKWIDFTRD
FLSKYTKTTSIDLSSLRPSSQYKDLGEYYAELNPLLYHISFQRIAEKEIMDAVETGKLYLFQ
IYNKDFAKGHHGKPNLHTLYWTGLFSPENLAKTSIKLNGQAELFYRPKSRMKRMAHRLGEKM
LNKKLKDQKTPIPDTLYQELYDYVNHRLSHDLSDEARALLPNVITKEVSHEIIKDRRFTSDK
FFFHVPITLNYQAANSPSKFNQRVNAYLKEHPETPIIGIDRGERNLIYITVIDSTGKILEQR
SLNTIQQFDYQKKLDNREKERVAARQAWSVVGTIKDLKQGYLSQVIHEIVDLMIHYQAVVVL
ENLNFGFKSKRTGIAEKAVYQQFEKMLIDKLNCLVLKDYPAEKVGGVLNPYQLTDQFTSFAK
MGTQSGFLFYVPAPYTSKIDPLTGFVDPFVWKTIKNHESRKHFLEGFDFLHYDVKTGDFILH
FKMNRNLSFQRGLPGFMPAWDIVFEKNETQFDAKGTPFIAGKRIVPVIENHRFTGRYRDLYP
ANELIALLEEKGIVFRDGSNILPKLLENDDSHAIDTMVALIRSVLQMRNSNAATGEDYINSP
VRDLNGVCFDSRFQNPEWPMDADANGAYHIALKGQLLLNHLKESKDLKLQNGISNQDWLAYI
QELRN

pCAG-2AeGFP backbone partial sequence

(CAG-NLS-*XmaI*-*NheI*-NLS-3xHA-T2A-eGFP-SV40**)**

gacattgattattgactagttattaatagtaatcaattacggggtcattagttcatagccca
tatatggagttccGCGTTACATAACTTACGGTAAATGGCCCGCCTGGCTGACCGCCCAACGA
CCCCCGCCCATTGACGTCAATAATGACGTATGTTCCCATAGTAACGCCAATAGGGACTTTCC
ATTGACGTCAATGGGTGGACTATTTACGGTAAACTGCCCACTTGGCAGTACATCAAGTGTAT
CATATGCCAAGTACGCCCCCTATTGACGTCAATGACGGTAAATGGCCCGCCTGGCATTATGC
CCAGTACATGACCTTATGGACTTTCCTACTTGGCAGTACATCTACGTATTAGTCATCGCTA
TTACCATGGGTCGAGGTGAGCCCCACGTTCTGCTTCACTCTCCCCATCTCCCCCCCCTCCCC

ACCCCCAATTTTGTATTTATTTATTTTTTAATTATTTTGTGCAGCGATGGGGGCGGGGGGG
GGGGGGCGCGCGCCAGGCGGGGCGGGGCGGGGCGAGGGGCGGGGCGGGGCGAGGCGGAGAGG
TGCGGCGGCAGCCAATCAGAGCGGCGCGCTCCGAAAGTTTCCTTTTATGGCGAGGCGGCGGC
GGCGGCGGCCCTATAAAAAGCGAAGCGCGCGGCGGGCGGGAGTCGCTGCGTTGCCTTCGCCC
CGTGCCCCGCTCCGCGCCGCCTCGCGCCGCCCGCCCCGGCTCTGACTGACCGCGTTACTCCC
ACAGGTGAGCGGGCGGGACGGCCCTTCTCCTCCGGGCTGTAATTAGCGCTTGGTTTAATGAC
GGCTCGTTTCTTTTCTGTGGCTGCGTGAAAGCCTTAAAGGGCTCCGGGAGGGCCCTTTGTGC
GGGGGGGAGCGGCTCGGGGGGTGCGTGCGTGTGTGTGTGCGTGGGGAGCGCCGCGTGCGGCC
CGCGCTGCCCGGCGGCTGTGAGCGCTGCGGGCGCGGCGCGGGGCTTTGTGCGCTCCGCGTGT
GCGCGAGGGGAGCGCGGCCGGGGGCGGTGCCCCGCGGTGCGGGGGGGCTGCGAGGGGAACAA
AGGCTGCGTGCGGGGTGTGTGCGTGGGGGGGTGAGCAGGGGGTGTGGGCGCGGCGGTCGGGC
TGTAACCCCCCCCTGCACCCCCCTCCCCGAGTTGCTGAGCACGGCCCGGCTTCGGGTGCGGG
GCTCCGTACGGGGCGTGGCGCGGGGCTCGCCGTGCCGGGCGGGGGGTGGCGGCAGGTGGGGG
TGCCGGGCGGGGCGGGGCCGCCTCGGGCCGGGGAGGGCTCGGGGGAGGGGCGCGGCGGCCCC
CGGAGCGCCGGCGGCTGTCGAGGCGCGGCGAGCCGCAGCCATTGCCTTTTATGGTAATCGTG
CGAGAGGGCGCAGGGACTTCCTTTGTCCCAAATCTGTGCGGAGCCGAAATCTGGGAGGCGCC
GCCGCACCCCCTCTAGCGGGCGCGGGGCGAAGCGGTGCGGCGCCGGCAGGAAGGAAATGGGC
GGGGAGGGCCTTCGTGCGTCGCCGCGCCGCCGTCCCCTTCTCCATCTCCAGCCTCGGGGCTG
TCCGCAGGGGGACGGCTGCCTTCGGGGGGGACGGGGCAGGGCGGGGTTCGGCTTCTGGCGTG
TGACCGGCGGCTCTAGcGCCTCTGCTAACCATGTTCATGCCTTCTTCTTTTTCCTACAGctc
ctgggcaacgtgctggttattgtgctgtctcatcattttggcaaaGCTAGTGAATTCTAATA
CGACTCACTATAGGCCGCCACCATGCCCAAGAAGAAGAGGAAGGTT*cccgggctagc*CCAA
AGAAGAAGAGGAAAGTCtctagaTACCCTTATGATGTTCCAGATTATGCCGGATACCCATAC
GATGTCCCTGACTATGCAGGCTCCTACCCTTATGACGTCCCAGACTACGCCggatccAGGTC
CGGCGGCGGAGAGGGCAGAGGAAGTCTTCTAACATGCGGTGACGTGGAGGAGAATCCCGGCC
CAATGGTGAGCAAGGGCGAGGAGCTGTTCACCGGGGTGGTGCCCATCCTGGTCGAGCTGGAC
GGCGACGTAAACGGCCACAAGTTCAGCGTGTCCGGCGAGGGCGAGGGCGATGCCACCTACGG
CAAGCTGACCCTGAAGTTCATCTGCACCACCGGCAAGCTGCCCGTGCCCTGGCCCACCCTCG
TGACCACCCTGACCTACGGCGTGCAGTGCTTCAGCCGCTACCCCGACCACATGAAGCAGCAC
GACTTCTTCAAGTCCGCCATGCCCGAAGGCTACGTCCAGGAGCGCACCATCTTCTTCAAGGA
CGACGGCAACTACAAGACCCGCGCCGAGGTGAAGTTCGAGGGCGACACCCTGGTGAACCGCA
TCGAGCTGAAGGGCATCGACTTCAAGGAGGACGGCAACATCCTGGGGCACAAGCTGGAGTAC
AACTACAACAGCCACAACGTCTATATCATGGCCGACAAGCAGAAGAACGGCATCAAGGTGAA
CTTCAAGATCCGCCACAACATCGAGGACGGCAGCGTGCAGCTCGCCGACCACTACCAGCAGA
ACACCCCCATCGGCGACGGCCCCGTGCTGCTGCCCGACAACCACTACCTGAGCACCCAGTCC
GCCCTGAGCAAAGACCCCAACGAGAAGCGCGATCACATGGTCCTGCTGGAGTTCGTGACCGC
CGCCGGGATCACTCTCGGCATGGACGAGCTGTACAAGtaactgcagcgcggggatctcatgc
tggagttcttcgcccaccccaacttgtttattgcagcttataatggttacaaataaagcaat
agcatcacaaatttcacaaataaagcatttttttcactgcattctagttgtggtttgtccaa
actcatcaatgtatctta

BPK2104-*ccdB* backbone partial sequence
(*lac*I-T7-*lac*O-NLS-*XmaI*-*SpeI*-His$_{10}$-terminator)

TCACTGCCCGCTTTCCAGTCGGGAAACCTGTCGTGCCAGCTGCATTAATGAATCGGCCAACG
CGCGGGGAGAGGCGGTTTGCGTATTGGGCGCCAGGGTGGTTTTTCTTTTCACCAGTGAGACG
GGCAACAGCTGATTGCCCTTCACCGCCTGGCCCTGAGAGAGTTGCAGCAAGCGGTCCACGCT
GGTTTGCCCCAGCAGGCGAAAATCCTGTTTGATGGTGGTTAACGGCGGGATATAACATGAGC
TGTCTTCGGTATCGTCGTATCCCACTACCGAGATGTCCGCACCAACGCGCAGCCCGGACTCG
GTAATGGCGCGCATTGCGCCCAGCGCCATCTGATCGTTGGCAACCAGCATCGCAGTGGGAAC
GATGCCCTCATTCAGCATTTGCATGGTTTGTTGAAAACCGGACATGGCACTCCAGTCGCCTT
CCCGTTCCGCTATCGGCTGAATTTGATTGCGAGTGAGATATTTATGCCAGCCAGCCAGACGC
AGACGCGCCGAGACAGAACTTAATGGGCCCGCTAACAGCGCGATTTGCTGGTGACCCAATGC
GACCAGATGCTCCACGCCCAGTCGCGTACCGTCTTCATGGGAGAAAATAATACTGTTGATGG
GTGTCTGGTCAGAGACATCAAGAAATAACGCCGGAACATTAGTGCAGGCAGCTTCCACAGCA
ATGGCATCCTGGTCATCCAGCGGATAGTTAATGATCAGCCCACTGACGCGTTGCGCGAGAAG
ATTGTGCACCGCCGCTTTACAGGCTTCGACGCCGCTTCGTTCTACCATCGACACCACCACGC
TGGCACCCAGTTGATCGGCGCGAGATTTAATCGCCGCGACAATTTGCGACGGCGCGTGCAGG
GCCAGACTGGAGGTGGCAACGCCAATCAGCAACGACTGTTTGCCCGCCAGTTGTTGTGCCAC
GCGGTTGGGAATGTAATTCAGCTCCGCCATCGCCGCTTCCACTTTTTCCCGCGTTTTCGCAG
AAACGTGGCTGGCCTGGTTCACCACGCGGGAAACGGTCTGATAAGAGACACCGGCATACTCT
GCGACATCGTATAACGTTACTGGTTTCACATTCACCACCCTGAATTGACTCTCTTCCGGGCG
CTATCATGCCATACCGCGAAAGGTTTTGCGCCATTCGATGGTGTCCGGGATCTCGACGCTCT
CCCTTATGCGACTCCTGCATTAGGAAATTAATACGACTCACTATAGGGGAATTGTGAGCGGA
TAACAATTCCCCTGTAGAAATAATTTTGTTTAACTTTAATAAGGAGATATCATATGCCCAAG
AAGAAGAGGAAGGTT*cccgggctagt*CATCACCATCACCACCATCATCACCATCACTAGGC
GGCCGCATAATGCTTAAGTCGAACAGAAAGTAATCGTATTGTACACGGCCGCATAATCGAAA
TTAATacgactcactataggGAATTCGGTACCtgagaataactagcaTAACCCCTTGGGGCC
TCTAAACGGGTCTTGAGGGGTTTTTTGCTGAAACCTCAGGCATTT

pUC19-U6-crRNA1 backbone partial sequence
(U6-tRNA-crRNA1-*BasI*-*BasI*-tRNA-terminator)
TGTAAAACGACGGCCAGTGAATTCGAGGGCCTATTTCCCATGATTCCTTCATATTTGCATAT
ACGATACAAGGCTGTTAGAGAGATAATTGGAATTAATTTGACTGTAAACACAAAGATATTAG
TACAAAATACGTGACGTAGAAAGTAATAATTTCTTGGGTAGTTTGCAGTTTTAAAATTATGT
TTTAAAATGGACTATCATATGCTTACCGTAACTTGAAAGTATTTCGATTTCTTGGCTTTATA
TATCTTGTGGAAAGGACGAAACACCGGAACAAAGCACCAGTGGTCTAGTGGTGGAATAGTAC
CCTGCCACGGTACAGACCCGGGTTCGATTCCCGGCTGGTGCAATTTCTACTATTGTAGATA*G*
*AGACC*GAGAGAG*GGTCTC*AAACAAAGCACCAGTGGTCTAGTGGTGGAATAGTACCCTGCCAC
GGTACAGACCCGGGTTCGATTCCCGGCTGGTGCANNNNNNNNNNNNNNNNNNNNNNNNNNNN
NNNNNNNNNNNNNNNNNNNNNNNNNNNNNNNNNNNNNNNNNNNNNNNNNNN*AAGCTT*GGCGT
AATCATGGTCATAGCTGTTTCCTG

pUC19-U6-crRNA2 backbone partial sequence
(U6-tRNA-crRNA2-*BasI*-*BasI*-tRNA-terminator)
TGTAAAACGACGGCCAGTGAATTCGAGGGCCTATTTCCCATGATTCCTTCATATTTGCATAT
ACGATACAAGGCTGTTAGAGAGATAATTGGAATTAATTTGACTGTAAACACAAAGATATTAG
TACAAAATACGTGACGTAGAAAGTAATAATTTCTTGGGTAGTTTGCAGTTTTAAAATTATGT

TTTAAAATGGACTATCATATGCTTACCGTAACTTGAAAGTATTTCGATTTCTTGGCTTTATA
TATCTTGTGGAAAGGACGAAACACCGGAACAAAGCACCAGTGGTCTAGTGGTGGAATAGTAC
CCTGCCACGGTACAGACCCGGGTTCGATTCCCGGCTGGTGCAATTTCTACTGTTGTAGATA*G*
*AGACC*GAGAGAG*GGTCTC*AAACAAAGCACCAGTGGTCTAGTGGTGGAATAGTACCCTGCCAC
GGTACAGACCCGGGTTCGATTCCCGGCTGGTGCANNNNNNNNNNNNNNNNNNNNNNNNNNNN
NNNNNNNNNNNNNNNNNNNNNNNNNNNNNNNNNNNNNNNNNNNNNNNNNNN*AAGCTT*GGCGT
AATCATGGTCATAGCTGTTTCCTG

pUC19-U6-crRNA5 backbone partial sequence
(U6-tRNA-crRNA5-*BasI*-*BasI*-tRNA-terminator**)**

TGTAAAACGACGGCCAGTGAATTCGAGGGCCTATTTCCCATGATTCCTTCATATTTGCATAT
ACGATACAAGGCTGTTAGAGAGATAATTGGAATTAATTTGACTGTAAACACAAAGATATTAG
TACAAAATACGTGACGTAGAAAGTAATAATTTCTTGGGTAGTTTGCAGTTTTAAAATTATGT
TTTAAAATGGACTATCATATGCTTACCGTAACTTGAAAGTATTTCGATTTCTTGGCTTTATA
TATCTTGTGGAAAGGACGAAACACCGGAACAAAGCACCAGTGGTCTAGTGGTGGAATAGTAC
CCTGCCACGGTACAGACCCGGGTTCGATTCCCGGCTGGTGCAATTTCTACTGTGTGTAGATA
*GAGACC*GAGAGAG*GGTCTC*AAACAAAGCACCAGTGGTCTAGTGGTGGAATAGTACCCTGCCA
CGGTACAGACCCGGGTTCGATTCCCGGCTGGTGCANNNNNNNNNNNNNNNNNNNNNNNNNNN
NNNNNNNNNNNNNNNNNNNNNNNNNNNNNNNNNNNNNNNNNNNNNNNNNNNN*AAGCTT*GGCG
TAATCATGGTCATAGCTGTTTCCTG

pUC19-U6-crRNA6 backbone partial sequence
(U6-tRNA-crRNA6-*BasI*-*BasI*-tRNA-terminator**)**

TGTAAAACGACGGCCAGTGAATTCGAGGGCCTATTTCCCATGATTCCTTCATATTTGCATAT
ACGATACAAGGCTGTTAGAGAGATAATTGGAATTAATTTGACTGTAAACACAAAGATATTAG
TACAAAATACGTGACGTAGAAAGTAATAATTTCTTGGGTAGTTTGCAGTTTTAAAATTATGT
TTTAAAATGGACTATCATATGCTTACCGTAACTTGAAAGTATTTCGATTTCTTGGCTTTATA
TATCTTGTGGAAAGGACGAAACACCGGAACAAAGCACCAGTGGTCTAGTGGTGGAATAGTAC
CCTGCCACGGTACAGACCCGGGTTCGATTCCCGGCTGGTGCAATTTCTACTACTGTAGATA*G*
*AGACC*GAGAGAG*GGTCTC*AAACAAAGCACCAGTGGTCTAGTGGTGGAATAGTACCCTGCCAC
GGTACAGACCCGGGTTCGATTCCCGGCTGGTGCANNNNNNNNNNNNNNNNNNNNNNNNNNNN
NNNNNNNNNNNNNNNNNNNNNNNNNNNNNNNNNNNNNNNNNNNNNNNNNNN*AAGCTT*GGCGT
AATCATGGTCATAGCTGTTTCCTG

pUC19-U6-crRNA7 backbone partial sequence
(U6-tRNA-crRNA7-*BasI*-*BasI*-tRNA-terminator**)**

TGTAAAACGACGGCCAGTGAATTCGAGGGCCTATTTCCCATGATTCCTTCATATTTGCATAT
ACGATACAAGGCTGTTAGAGAGATAATTGGAATTAATTTGACTGTAAACACAAAGATATTAG
TACAAAATACGTGACGTAGAAAGTAATAATTTCTTGGGTAGTTTGCAGTTTTAAAATTATGT
TTTAAAATGGACTATCATATGCTTACCGTAACTTGAAAGTATTTCGATTTCTTGGCTTTATA
TATCTTGTGGAAAGGACGAAACACCGGAACAAAGCACCAGTGGTCTAGTGGTGGAATAGTAC
CCTGCCACGGTACAGACCCGGGTTCGATTCCCGGCTGGTGCAATTTCTACTTTGGTAGATA*G*
*AGACC*GAGAGAG*GGTCTC*AAACAAAGCACCAGTGGTCTAGTGGTGGAATAGTACCCTGCCAC
GGTACAGACCCGGGTTCGATTCCCGGCTGGTGCANNNNNNNNNNNNNNNNNNNNNNNNNNNN

NNNNNNNNNNNNNNNNNNNNNNNNNNNNNNNNNNNNNNNNNNNNNN*AAGCTT*GGCGT
AATCATGGTCATAGCTGTTTCCTG

pUC19-U6-crRNA4n96 backbone partial sequence

(U6-tRNA-crRNA4n96-*BasI*-*BasI*-tRNA-terminator**)**

TGTAAAACGACGGCCAGTGAATTCGAGGGCCTATTTCCCATGATTCCTTCATATTTGCATAT
ACGATACAAGGCTGTTAGAGAGATAATTGGAATTAATTTGACTGTAAACACAAAGATATTAG
TACAAAATACGTGACGTAGAAAGTAATAATTTCTTGGGTAGTTTGCAGTTTTAAAATTATGT
TTTAAAATGGACTATCATATGCTTACCGTAACTTGAAAGTATTTCGATTTCTTGGCTTTATA
TATCTTGTGGAAAGGACGAAACACCGGAACAAAGCACCAGTGGTCTAGTGGTGGAATAGTAC
CCTGCCACGGTACAGACCCGGGTTCGATTCCCGGCTGGTGCAATTTCTACTATGGTAGATA*G*
*AGACC*GAGAGAG*GGTCTC*AAACAAAGCACCAGTGGTCTAGTGGTGGAATAGTACCCTGCCAC
GGTACAGACCCGGGTTCGATTCCCGGCTGGTGCANNNNNNNNNNNNNNNNNNNNNNN
NNNNNNNNNNNNNNNNNNNNNNNNNNNNNNNNNNNNNNNNNNNNNN*AAGCTT*GGCGT
AATCATGGTCATAGCTGTTTCCTG

pUC19-U6-tRNA backbone partial sequence

(U6-tRNA-*BasI*-*BasI*-tRNA-terminator**)**

TGTAAAACGACGGCCAGTGAATTCGAGGGCCTATTTCCCATGATTCCTTCATATTTGCATAT
ACGATACAAGGCTGTTAGAGAGATAATTGGAATTAATTTGACTGTAAACACAAAGATATTAG
TACAAAATACGTGACGTAGAAAGTAATAATTTCTTGGGTAGTTTGCAGTTTTAAAATTATGT
TTTAAAATGGACTATCATATGCTTACCGTAACTTGAAAGTATTTCGATTTCTTGGCTTTATA
TATCTTGTGGAAAGGACGAAACACCGGAACAAAGCACCAGTGGTCTAGTGGTGGAATAGTAC
CCTGCCACGGTACAGACCCGGGTTCGATTCCCGGCTGGTGCAA*GAGACC*GAGAGAG*GGTCTC*
AAACAAAGCACCAGTGGTCTAGTGGTGGAATAGTACCCTGCCACGGTACAGACCCGGGTTCG
ATTCCCGGCTGGTGCAttttttttAAGCTTGGCGTAATCATGGTCATAGCTGTTTCCTG
